# Two decades of continuous progresses and breakthroughs in the field of bioactive ceramics and glasses driven by CICECO-hub scientists

**DOI:** 10.1016/j.bioactmat.2024.05.041

**Published:** 2024-06-08

**Authors:** H.R. Fernandes, S. Kannan, M. Alam, G.E. Stan, A.C. Popa, R. Buczyński, P. Gołębiewski, J.M.F. Ferreira

**Affiliations:** a Department of Materials and Ceramic Engineering, CICECO-Aveiro Institute of Materials, University of Aveiro, Santiago University Campus, 3810-193, Aveiro, Portugal; b Centre for Nanoscience and Technology, Pondicherry University, 605014, Puducherry, India; c National Institute of Materials Physics, 077125, Magurele, Romania; d Łukasiewicz Research Network – Institute of Microelectronics and Photonics, Al. Lotników 32/46, 02–668, Warsaw, Poland

**Keywords:** CaP bioceramics, Alkali-free bioactive glasses, Bio-functional doping/substitution, Osseointegration, Antimicrobial efficiency

## Abstract

Over the past two decades, the CICECO-hub scientists have devoted substantial efforts to advancing bioactive inorganic materials based on calcium phosphates and alkali-free bioactive glasses. A key focus has been the deliberate incorporation of therapeutic ions like Mg, Sr, Zn, Mn, or Ga to enhance osteointegration and vascularization, confer antioxidant properties, and impart antimicrobial effects, marking significant contributions to the field of biomaterials and bone tissue engineering. Such an approach is expected to circumvent the uncertainties posed by methods relying on growth factors, such as bone morphogenetic proteins, parathyroid hormone, and platelet-rich plasma, along with their associated high costs and potential adverse side effects. This comprehensive overview of CICECO-hub's significant contributions to the forefront inorganic biomaterials across all research aspects and dimensionalities (powders, granules, thin films, bulk materials, and porous structures), follows a unified approach rooted in a cohesive conceptual framework, including synthesis, characterization, and testing protocols. Tangible outcomes [injectable cements, durable implant coatings, and bone graft substitutes (scaffolds) featuring customized porous architectures for implant fixation, osteointegration, accelerated bone regeneration in critical-sized bone defects] were achieved. The manuscript showcases specific biofunctional examples of successful biomedical applications and effective translations to the market of bone grafts for advanced therapies.

## Highlights

• Two decades of CICECO-hub advancements in the field of bioactive materials.
• Smart doping with metallic ions – a powerful tool for enhancing bio-functionality.
• Swift quality solutions to the increasingly frequent bone healthcare challenges.

## Graphical abstract

**Figure:**
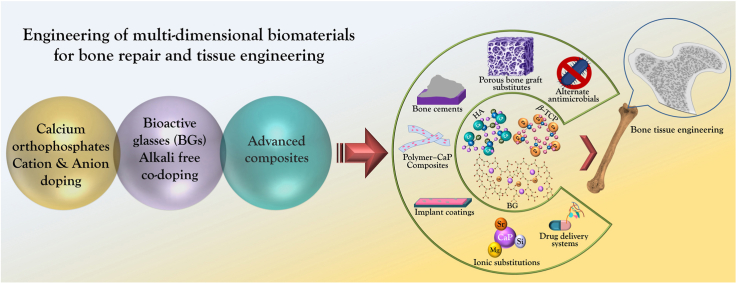

## 1 Introduction

**Fig. 1:**
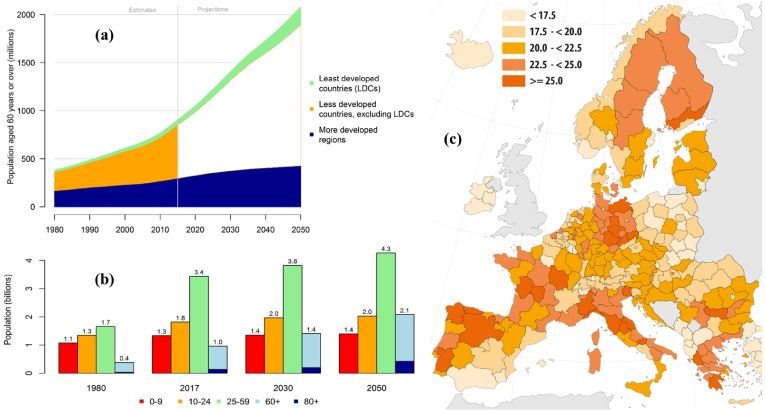
Number of persons aged 60 years or over by development group, from 1980 to 2050 (a), global population by broad age group (b) and, % of total population aged 65 or over in Europe (January 2020, Nomenclature of territorial units for statistics (NUTS2)) (c) [1].

The world's population is ageing and the growth in the number of older persons is a global phenomenon. It is expected that up to 2050, nearly every country in the world will experience a considerable increase of population aged 60 years or over (Fig. 1), with strong repercussions in all segments of society, including health and quality of life [1]. This worldwide increase of life expectancy is intrinsically associated with an increasing incidence of skeletal diseases and has generated higher healthcare demands [2,3]. Specifically, and most frequently, medical remedy solutions for bone diseases (*e.g.*, osteoporosis, osteoarthritis, osteosarcoma) and traumatic accidents that entail bone repairment are frantically searched for. For such prevalent problems, the biomedical community is looking for adequate bone grafting materials, scaffold designs and surgical procedures [[4], [5], [6]]. Healthcare entities around the world are working to promote healthy ageing and to prevent and treat chronic conditions [1]. Therefore, increasing research efforts are focused in developing new implantable materials as well as improving the manufacturing techniques to fabricate implant devices aiming at regenerating and repairing living tissues damaged by disease or trauma [7].

Currently, the gold standard in bone repair is the autogenous bone graft (autograft), which can foster the best osteogenic potential and lacks antigenic effects [5,6,[8], [9], [10]]. However, autografts also carry several important drawbacks, such as morbidity derived from bone harvesting, additional pain, more complex surgical procedures, limited bone material, or on-site infections [5,6,[8], [9], [10]]. The next best option are the allografts (based on processed bone obtained from human donors). Still, they bear both advantages and limitations, being recognized to pose higher risk of infection and lower structural strength [5,6,8,9,11] with respect to autografts. Xenografts (based on processed bone harvested from various animal species) cannot be considered a superior alternative to autografts or allografts since their immunogenicity risks are significantly higher [9,12]. Recently, the prospect of large-scale fabrication of bone graft substitutes based on synthetic biomaterials generated new hope for this specific application segment of the biomedical field [[13], [14], [15], [16], [17], [18]]. A rapid advancement concomitant with a fabrication cost-reduction, and thereby an increasing accessibility of the 3D printing techniques created much anticipation and generated the premises of fast-forwarding the bench-to-bedside pathway of such macro-porous grafting constructs (bone scaffolds) [14,15,17]. Two families of synthetic biomaterials emerged as prominent candidate materials for bone scaffolding: the calcium phosphates (markedly bi-phasic calcium phosphates) and the silica-based bioactive glasses [[19], [20], [21]]. Lately tremendous progress in the development of synthetic bone grafts by the virtue of functional coatings, which involves calcium phosphates (CaPs) especially with cationic substitutions (Cu^2+^, Sr^2+^, Ag^+^) on both metallic and non-metallic implants was witnessed, prompting osteoconductivity and antibacterial efficacy [[22], [23], [24]]. Additionally, composites involving calcium phosphates and inert ceramics have demonstrated improvement in the mechanical aspirations of bone substitutes and emerged as a pivotal candidate for bone tissue engineering [[25], [26], [27]]. Recently, the incorporation of bio-inorganic agents (particularly, cations such as magnesium, silver, zinc, strontium, copper, manganese or gallium) were found to boost the functionality of these inorganic compounds, leading not only to improved bio-responses (*e.g.*, cell adhesion, osteogenesis, angiogenesis, anti-oxidative properties and/or antibacterial efficacy), but also to enhanced mechanical and textural features and to adapted processability (for instance, in terms of matching the coefficient of thermal expansion of metallic implants or of tailoring the glass transition temperature to foster an enlarged manufacturability window) [[28], [29], [30], [31], [32]]. Over the last two decades, the CICECO-Aveiro Institute of Materials was a pivotal dynamic actor in the fields of (*i*) synthesis of advanced calcium phosphates (CaPs) and design of innovative bioactive glass (BGs) formulations with superior biological performances and (*ii*) implementation of delineated materials into 3D porous constructs for bone repair and tissue engineering applications; (*iii*) pushing some bioactive materials toward the market. Diligently engaged at the forefront of scientific and technological development and knowledge in this realm of designing osteoconductive (CaPs) and osteoproductive (BGs) synthetic materials and refining/instituting cost-efficient additive manufacturing fabrication protocols of bone scaffolds, the researchers at the University of Aveiro (UA) got involved in several symbiotic national and international collaborations and published over the period 2002–2023 a number of 447 scientific articles, which gathered over 14100 citations (*h*-index = 64) – a testimony of their work relevancy and impact in the biomaterials community. Of these, the Advanced Materials Processing group, led by Prof. José M.F. Ferreira, co-authored more than 148 Clarivate-Web of Science™-indexed articles (thus a share of ∼33 % of the UA total), attracting over 6100 citations (thus a share of ∼43 % of the UA total) (*h*-index = 50) (Database: Clarivate—Web of Science™ Core Collection. Search keywords: “Universidade de Aveiro” AND “bioactive glass” OR “bioglass” OR “calcium phosphate” or “hydroxyapatite”).

This overview, tackling both (*I*) bioceramics and (*II*) silica-based bioactive glasses, aims to present a synopsis of selected topical advances achieved over the course of the years, hinging the CICECO-Aveiro Institute of Materials researchers with their valuable national and international collaborators in the field of synthetic inorganic multi-dimensional bioactive materials for bone repair and tissue engineering. This review is justified by two main reasons: (*i*) The previous publications, including reviews, rarely fine-scrutinize the influence of the main factors affecting the overall output in terms of performance of biomaterials based on CaPs and SBGs. Most of them do not go much beyond gathering together somewhat disconnected data published in research works from various worldwide groups, thus lacking control over the uniformity in applied synthesis and characterization methods, and consequently, leading to dependencies on material properties induced by such procedural heterogeneities. Such randomness aspects contrast with our comprehensive overview of CICECO-hub's significant contributions to the forefront inorganic biomaterials across all relevant research aspects and dimensionalities (powders, granules, thin films, bulk materials, and porous structures), following a unified approach rooted in a cohesive conceptual framework; *ii*) In the particular case of SBGs, it is worth highlighting that the majority of research articles and reviews on SBGs have predominantly focused on high alkali-containing compositions, often overlooking the frequently better-performing melt-quenched alkali-free counterparts until very recently [33]. In their 2024 review, Shearer et al. [33] briefly underscored the emerging benefits of alkali-free bioactive glasses (AFBGs), highlighting their improved cytocompatibility, reduced tendency for crystallization, and optimized dissolution rate. Indeed, as will be comprehensively demonstrated throughout this review, AFBGs exhibit remarkable durability while retaining their innate ability to bond to bone and promote bone growth, while also facilitating the integration of additional biofunctionalities like anti-oxidant properties and anti-microbial efficacy. However, numerous researchers tend to valuate more “fashion” in detriment of “performance”, struggling to escape unfavourable comparisons in terms of properties and overall performances. Their stubbornness contrasts with the clear response of artificial intelligence (AI) to a recent open question about the hot topics in glass research. It should be recognized that AI instruments, while not yet fully matured, hold the potential to provide a more objective and impartial assessment of scientific progress compared to traditional reviews, which may be susceptible to biases and habitual inclinations. Among the foremost examples pointed out by ChatGPT AI (one of the most popular virtual assistants, developed by OpenAI), AFBGs for healthcare applications, such as bone repair, wound healing, and tissue engineering, were highly ranked, following research glasses for energy applications (*e.g.*, Li-ion conducting glasses for use in lithium-ion batteries) and the long-term sustainability of glass fabrication. However, the multitude of favourable properties of AFBGs justifies heightened efforts to disseminate their virtues, reaching beyond materials researchers to encompass biologists and health professionals in dentistry, orthopaedics, and maxillofacial surgery for both humans and veterinary applications.

## 2 Bioceramics advances

Bioceramics in bone remodelling applications have been the subject of major interest for the past five decades. A wide range of bioceramics categorized into bioactive and bioinert have been employed for such purpose that mainly depends on the requirement of biocompatibility and mechanical compatibility. Hydroxyapatite [Ca_10_(PO_4_)_6_(OH)_2_, HA], *β*-tricalcium phosphate [*β*-Ca_3_(PO_4_)_2_, *β*-TCP], and biphasic calcium phosphates, comprising of HA and *β*-TCP blends [BCP] are the key bioactive ceramics widely considered in bone remodelling applications due to their salient features of chemical similarity with the major inorganic component of bone mineral, biocompatibility, and their capacity to ensure rapid new bone formation at the defective site. While, partially stabilized tetragonal zirconia [*t*-ZrO_2_], zirconia toughened alumina [ZTA], and *α*-alumina [*α*-Al_2_O_3_] fall into the category of bioinert ceramics. Exceptional mechanical strength and high corrosion resistance are considered the favourable features of these bioinert ceramics.

### 2.1 Crystal structure of bioceramics

**Fig. 2:**
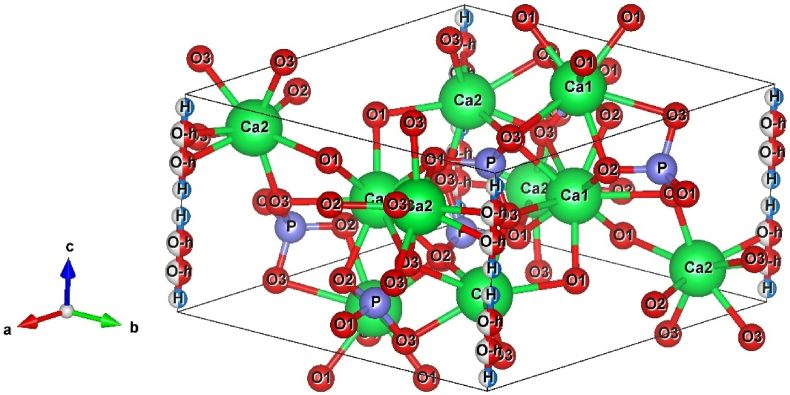
Crystallographic projection of HA.

HA exhibits hexagonal crystal structure with *P*6_3_/*m(176)* space group and lattice parameters of *a* = *b*- 9.432 Å, *c* = 5 6.881 Å as reported by Kay et al. [34]. HA (Fig. 2) comprises a hexagonal stack of isolated PO_4_^3−^ tetrahedrons that create two kinds of channels parallel to the *c*-axis. The first channel consists of Ca^2+^(1) ions form CaO_9_-polyhedra, while the second one is lined by oxygen and other Ca^2+^ ions alongside the presence of OH^−^ anions. The Ca^2+^(2) ions at z = ¼ and z = 3/4 form triangles with each Ca^2+^ ion at the corner of the triangle bonded to the central OH^−^ anion. The diameter of such channels (3 Å in HA) gives apatite ion exchanger properties, but only at high temperatures, and it can also act as a host to small molecules.

Tricalcium phosphate usually exists in three different polymorphs namely *β-*, *α*-*, α*′-Ca_3_(PO_4_)_2_, which are temperature dependent. The maximum thermal stability of pure form of *β-*Ca_3_(PO_4_)_2_ polymorph can stretch up to 1180 °C. *α*-Ca_3_(PO_4_)_2_ retains stability until 1470 °C, whilst *α*′-Ca_3_(PO_4_)_2_ is stabilized beyond 1470 °C. The allotropic *β-*Ca_3_(PO_4_)_2_ → *α*-Ca_3_(PO_4_)_2_ transition beyond 1180 °C is delayed by the addition of impurities at the crystal lattice of Ca_3_(PO_4_)_2_ [[35], [36], [37]]. *β-*TCP polymorph is well-established in biomedical field, alongside HA, and it is known to endow superior dissolution characteristics in comparison to HA [38]. The preparation of pure *β-*TCP is accomplished by various methods like solid state reaction, co-precipitation, hydrothermal, solvothermal, and sol-gel.

**Fig. 3:**
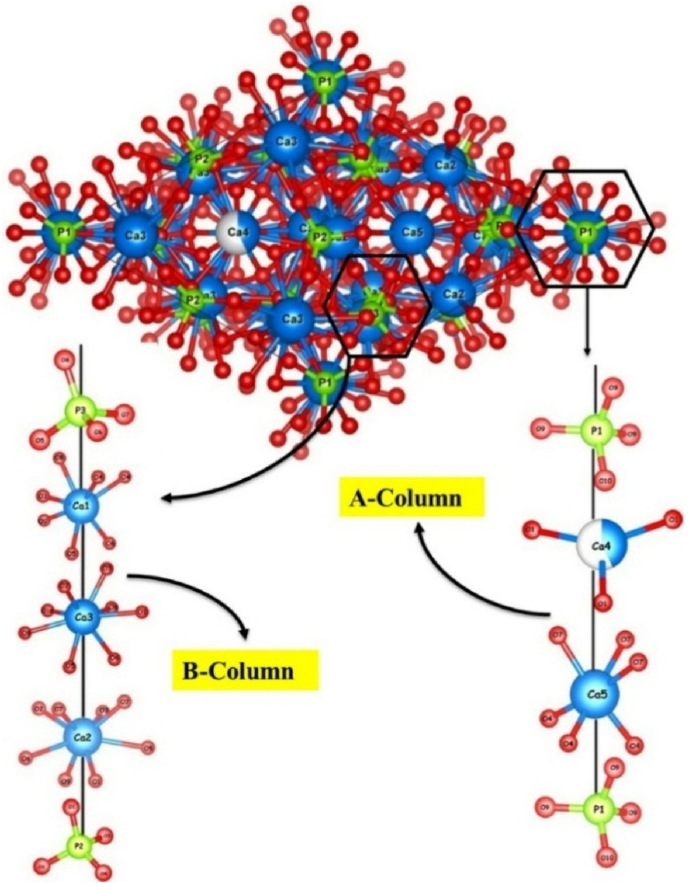
Crystallographic projection of β-TCP comprising two different columns.

*β-*TCP (Fig. 3) exhibits rhombohedral crystal structure (space group = *R-3c (167)*) with unit cell parameters *a* = *b*-axis of 10.439 Å and *c*-axis of 37.375 Å, as reported by Yashima et al. [39]. Generally, *β-*TCP structure is categorized into A and B columns with the former representing less dense Ca^2+^(4), Ca^2+^(5) and P (1) sites, while the latter is presented with a relatively denser environment with Ca^2+^(1), Ca^2+^(2), Ca^2+^(3), P (2) and P (3) sites. A total of 18 individual atomic positions are noticed in the unit cell of *β-*TCP: namely, 5 different Ca^2+^ sites (three in site *18b* and two in site *6a* at one-half occupancy), 3 P^5+^ positions (two in site *18b* and one in site *6a*), and 10 O^2−^ positions (nine in site *18b* and one in site *6a*). All Ca^2+^ and P^5+^ ions display variable coordination with oxygen. Ca^2+^(1), Ca^2+^(2), Ca^2+^(3), Ca^2+^(4) and Ca^2+^(5) exhibit seven-fold, six or eight-fold, eight-fold, three-fold and six-fold coordination with oxygen atoms, respectively. Ca^2+^(4) is the defective among the five different Ca^2+^sites in *β-*TCP, a special feature that provides structural flexibility to accommodate a wide range of substitutions in its lattice.

*T*-ZrO_2_ is the most investigated in the zirconia family due to its highly-relevant features, such as excellent wear resistance, chemical stability, and better fracture toughness and hardness [40]. The reversible nature of *t*-ZrO_2_→ *m*-ZrO_2_ phase transition makes it rather difficult to retain *t*-ZrO_2_ polymorph at room-temperature (RT). The *t*-ZrO_2_→ *m*-ZrO_2_ phase transition during cooling process is accompanied by a steady volume surge alongside crack development which lead to the failure of ZrO_2_ ceramics [41,42]. Due to this drawback, many reinforcing oxide systems (*e.g.*, CaO, MgO, Y_2_O_3_, La_2_O_3_ and CeO_2_ [[43], [44], [45], [46]]) have been used to retain *t*-ZrO_2_ polymorph at RT. The incorporation of divalent (Ca^2+^, Mg^2+^), trivalent ions (Y^3+^, La^3+^) or tetravalent (Zr^4+^) in the ZrO_2_ system develops oxygen vacancies that induce significant lattice deformation and consequently stabilize *t*-ZrO_2_.

In biomedical applications, *α*-Al_2_O_3_ is employed in load bearing orthopaedics due to its high strength as well as its excellent corrosion and wear resistance [47,48]. Al_2_O_3_ exhibits two different metastable polymorphs: namely, hexagonal close-packed (HCP) and face-centred cubic (FCC) structures that depend on the positioning of oxygen anions. *α*-Al_2_O_3_ exhibits trigonal symmetry with rhombohedral setting [space group *R-3c* (167)]. The crystal structure of *α*-Al_2_O_3_ is deliberated as a hexagonal closed packing with the coordination of oxygen anion at 2/3 of octahedral sites of Al^3+^ cation.

### 2.2 Formation mechanism of bioceramics

Chemical precipitation technique is the most common route to synthesize HA. The as-prepared powders display poor crystallinity at RT, being generally coined as amorphous calcium apatite (ACP). This ACP transforms into crystalline HA beyond 800 °C in accordance with the mechanism depicted (Eq. (1)) below:

(Eq. 1) $$10\;\text{Ca}{\left(\text{OH}\right)}_{2}+6{\left(NH_{4}\right)}_{2}\text{HP}O_{4}\to Ca_{10}{\left(PO_{4}\right)}_{6}{\left(\text{OH}\right)}_{2}+6H_{2}O+12NH_{4}\text{OH}$$

Similar to HA, wet-precipitation is also a commonly adopted method to synthetize *β-*TCP. The formation mechanism (Eqs. (eq. 2), (eq. 3), (eq. 4))) of *β-*TCP is illustrated below [49,50].

(eq. 2) $$Ca_{9}\left(\text{HP}O_{4}\right){\left(PO_{4}\right)}_{5}\left(\text{OH}\right)\overset{\;}{\to }3Ca_{3}(P{O_{4})}_{2}+H_{2}O$$

(eq. 3) $$2\mathrm{HP}O_{4}^{2-}\overset{\;\Delta 600{}^{\circ}C\;}{\to }P_{2}O_{7}+H_{2}O$$

(eq. 4) $$P_{2}O_{7}^{4-}+2OH^{-}\overset{\;\Delta 700-800{}^{\circ}C\;}{\to }2{\mathrm{PO}}_{4}^{3-}+H_{2}O$$

The above reaction mechanisms indicate the formation of calcium deficient apatite [Ca_9_(HPO_4_) (PO_4_)_5_(OH), CDA] in the as-precipitated condition, followed by its transformation into *β-*TCP, that is influenced by the heat-treatment conditions. Eqs. (eq. 3), (eq. 4)) illustrate the transformation of hydrogen phosphate (HPO_4_^2−^) into phosphate (PO_4_^3−^) ions.

The mixtures of HA and *β-*TCP, termed as BCPs, possess the advantages of stable HA and bioresorbable *β-*TCP, which are reported to exhibit improved osteogenic properties. BCP is attained by either mechanical mixing of individual HA and *β-*TCP in definite proportions or through transforming the as-synthesized calcium deficient apatite (molar Ca/P ratio <1.67) to yield HA and *β-*TCP mixtures beyond 750 °C (Eq. (5)) [51,52].

(eq. 5) $$Ca_{10-x}(\text{HP}{O_{4})}_{x}(P{O_{4})}_{6}-x{\left(\text{OH}\right)}_{2-x}\to \left(1-x\right)Ca_{10}{\left(PO_{4}\right)}_{6}{\left(\text{OH}\right)}_{2}+3\text{xC}a_{3}{\left(PO_{4}\right)}_{2}+xH_{2}O$$

### 2.3 Biological significance of ionic substitutions in HA and β-TCP

Despite the crystallographic and biological significance of synthetic HA and *β-*TCP, the biological apatites are usually non-stoichiometric with structural deficiencies that are mainly influenced by the presence of many trace elements in the crystal structure [[53], [54], [55]]. Table 1 lists the elemental composition and structural parameters of biological apatite [53,56]. Notwithstanding, these trace elements are reported to play significant roles on the biological process upon their implantation at the defective site [[57], [58], [59], [60], [61], [62], [63], [64], [65]]. The biological effects of ions incorporated into calcium phosphates, with the Table of Elements being exploited in an anticipated, fortuitous, or sometimes, surprizing manner, have been recently comprehensively overviewed [29,66]. Here, the salient features of only few of the most prominent trace elements will be highlighted and explained. Sodium, which is subsequent to calcium and phosphorus in terms of their biological presence is reported to exhibit a significant role on osteoporosis and bone metabolism [60,63]. Magnesium influences bone fragility, calcification procedures and also plays an important role in bone metabolism [57,59,65]. Further, Mg deficiency is reported as a probable risk aspect for osteoporosis in humans. The active role of potassium in biochemical and mineralization processes is well-documented [64]. The ability of fluorine to stabilize apatite structure and their preventive role on dental caries is reported [58,62], while chlorine creates a local acidic environment in the bone matrix, which consequently stimulates osteoclasts during the bone resorption process [61]. The impact of these trace elements in the biological process has stimulated numerous studies over the last two decades targeting the doping of HA and *β-*TCP either with single elements [[67], [68], [69], [70], [71]] or with coupled elemental substitutions [29,[72], [73], [74], [75], [76]].

**Table 1:** Elemental composition and structural parameters of biological apatite [53,56].

Composition (wt.%)	Enamel	Dentin	Bone	HA
Calcium	36.5	35.1	34.8	39.6
Phosphorous	17.7	16.9	15.2	18.5
Ca/P molar ratio	1.63	1.61	1.71	1.67
Sodium	0.5	0.6	0.9	–
Magnesium	0.44	1.25	0.72	–
Potassium	0.08	0.05	0.03	–
Carbonate	3.5	5.6	7.4	–
Fluoride	0.01	0.06	0.03	–
Chloride	0.30	0.01	0.13	–
**Crystallographic parameters (± 0.003 Å)**				
*a*-axis (Å)	9.441	9.421	9.41	9.430
*c*-axis (Å)	6.880	6.887	6.89	6.891
Ignition products (800 °C)	*β*-TCP + HA	*β*-TCP + HA	HA + CaO	HA

Other than the applications of calcium phosphate in bone tissue engineering, CaP-based probes are used in bioimaging as they resolve the issues of stability, hydrophilicity/hydrophobicity, size control, biocompatibility, binding of the target molecules that are experienced by their counterparts [77]. CaP-based nanoparticles are often used for targeted and localized drug delivery, enabling the minimum systemic dosage for a maximum therapeutic efficiency at the target site. Moreover, calcium and phosphate are their main degradation products, which are inherent constituents of the body fluids [78]. The ability of lanthanides to mimic the functions of Ca^2+^ by playing a significant role in the treatment of osteoporosis was reported in previous studies [79]. Ln^3+^ can functionally impersonate Ca^2+^ (1.00 Å) in CaPs due to (*i*) its similar ionic size ranging from 0.84 to 1.06 Å; and (*ii*) bonding behaviour of Ln^3+^, which is essentially ionic with a slight covalent contribution from *s*-orbitals [79].

### 2.4 Influence of dopants on the crystal lattice of CaPs

Ionic substitutions in the crystal lattice of CaPs have been documented as being mainly dependent on the local environment, nature of the dopant, size and concentration. *β-*TCP displays more structural flexibility to accommodate wide range of ions than HA. This is mainly due to the two different Ca^2+^ sites offered by HA, while the *β-*TCP exhibits five different Ca^2+^ sites. Further, the defective and half-filled Ca^2+^ (4) site of *β-*TCP shows more flexibility to accommodate a wide range of ions in its structure. Among the ions, cations are rather easily accommodated in *β-*TCP, whereas anionic substitutions namely chlorine (Cl^−^), fluorine (F^−^) and carbonates (CO_3_^2−^) can be easily incorporated into HA's lattice. Both partial and complete Cl^−^ and F^−^ accommodations (Eqs. (eq. 6), (eq. 7))) are favoured at the OH^−^ site of HA, whereas CO_3_^2−^ can substitute at both OH^−^ and PO_4_^3−^ sites.

(eq. 6) $$10\mathrm{Ca}{\left({\mathrm{NO}}_{3}\right)}_{2}+6{\left({\mathrm{NH}}_{4}\right)}_{2}{\mathrm{HPO}}_{4}+{\mathrm{xNH}}_{4}F+{\mathrm{yNH}}_{4}\mathrm{Cl}+\left[8-\left(x+y\right)\right]{\mathrm{NH}}_{4}\mathrm{OH}\;\to {\mathrm{Ca}}_{10}{\left({\mathrm{PO}}_{4}\right)}_{6}\left[\left(\mathrm{OH}\right)2‐\left(x+y\right){\left(F\right)}_{x}{\left(\mathrm{Cl}\right)}_{y}\right]+20{\mathrm{NH}}_{4}{\mathrm{NO}}_{3}+6H_{2}O$$

(eq. 7) $$10\mathrm{Ca}{\left({\mathrm{NO}}_{3}\right)}_{2}+6{\left({\mathrm{NH}}_{4}\right)}_{2}{\mathrm{HPO}}_{4}{+\mathrm{NH}}_{4}{F+\mathrm{NH}}_{4}\mathrm{Cl}+6{\mathrm{NH}}_{4}{\mathrm{OH}\;\to \mathrm{Ca}}_{10}{\left({\mathrm{PO}}_{4}\right)}_{6}\left(F\right)\left(\mathrm{Cl}\right)+20{\mathrm{NH}}_{4}{\mathrm{NO}}_{3}+6H_{2}O$$

There are accepted three types of CO_3_^2−^ substitutions in HA, *i.e.*, (*i*) incorporation at OH^−^ sites termed as A-type; (*ii*) accommodation at PO_4_^3−^ sites described as B-type; and (*iii*) the mixed AB-type that is facilitated by their simultaneous occupancy at OH^−^ and PO_4_^3−^ sites. A-type substitution is easily achieved by annealing HA in a CO_2_ environment [80,81] that is enabled by the net charge balance compensated through the occupancy of one CO_3_^2−^ group for two OH^−^ ions in accordance with the mechanism given below (Eq. (8)). Nevertheless, the bone mineral is reported to be a mixed AB-type substitution [82,83]. The mixed AB-type can be accomplished by the simultaneous substitution of Cl^−^, F^−^ and CO_3_^2−^ in the HA lattice [84].

(eq. 8) $${\mathrm{Ca}}_{10}{\left({\mathrm{PO}}_{4}\right)}_{6}{\left(\mathrm{OH}\right)}_{2}{+\;\mathrm{xCO}}_{2}{\to \mathrm{Ca}}_{10}{\left({\mathrm{PO}}_{4}\right)}_{6}{\left(\mathrm{OH}\right)}_{2‐2x}{\left({\mathrm{CO}}_{3}\right)}_{x}{+\;\mathrm{xH}}_{2}O$$

The attempt to substitute cations in HA structure yielded BCP mixtures owned to the non-stoichiometric nature displayed by HA. The stoichiometry of HA is displayed by the exact Ca/P molar ratio of 1.67, while the non-stoichiometric HA is ensured either by increasing or decreasing the Ca/P ratio. The attainment of this non-stoichiometry is dependent on synthesis conditions such as precipitation, hydrolysis, pH, and temperature. Calcium deficiency is deliberately created during the synthesis either by reducing the Ca or enhancing the P content of the precursor that results in the formation of BCP mixtures [85,86].

A handful number of investigations on single or coupled ionic substitutions in calcium phosphates have shown to lead to the formation of biphasic mixtures. The attempt to substitute divalent Mg^2+^ in CaPs with variable Ca/P ratios yielded BCP mixtures. The HA and *β-*TCP phase contents of the resultant mixtures were dependent upon the initial Ca/P ratio considered for the study [70,87]. The more accentuated calcium deficiency or non-stoichiometry of the starting powders, the greater was the *β-*TCP content of the BCP mixtures. Further, the lower sized Mg^2+^ preferred to occupy the Ca^2+^(5) lattice site of *β-*TCP, which is mainly due to the minimum bond length of Ca^2+^(5)−O favouring its occupancy [70,87]. The favourable substitution of Mg^2+^ in the *β-*TCP structure of the biphasic mixtures is illustrated in accordance to the mechanism (Eq. 9) given below.

(eq. 9) $${\text{Ca}}_{10‐x}{\left({\text{MgHPO}}_{4}\right)}_{x}{\left(PO_{4}\right)}_{6‐x}{\left(\text{OH}\right)}_{2‐x}\to {\text{Ca}}_{10‐x}{\left({\text{Mg}}_{2}P_{2}O_{7}\right)}_{x/2}{\left({\text{PO}}_{4}\right)}_{6‐x}{\left(\text{OH}\right)}_{2‐x}+x/2H_{2}{\text{OCa}}_{10‐x}{\left({\text{Mg}}_{2}P_{2}O_{7}\right)}_{x/2}{\left({\text{PO}}_{4}\right)}_{6‐x}{\left(\text{OH}\right)}_{2‐x}+x/2H_{2}O\to \left(1-x\right){\text{Ca}}_{10}{\left({\text{PO}}_{4}\right)}_{6}{\left(\text{OH}\right)}_{2\;}{{+3x(\text{CaMg}}_{2x/9})}_{3}{\left({\text{PO}}_{4}\right)}_{2}{+\text{xH}}_{2}O$$

The individual substitution of monovalent elements like K^+^ (1.38 Å) and Na^+^ (1.02 Å) for the Ca^2+^ (1.00 Å) favours the Ca^2+^ sites of HA lattice in BCP mixtures [88,89]. Nevertheless, their occupancy is limited in HA lattice, while their excess addition facilitated accommodation in the *β-*TCP. Other than single substitutions, the coupled and multiple cationic substitutions in calcium phosphates favoured the formation of BCP mixtures, with their preferential occupancy at the *β-*TCP lattice sites [55,76,84,[89], [90], [91]]. Further, the mixed substitutions involving cations and anions favoured the accommodation of the former in *β-*TCP lattice, with the latter showing an occupancy preference for HA structure [69].

The above-mentioned facts indicated the difficulty in attaining unique solid solution of HA through the incorporation of dopants. The formation of single phase *β-*TCP solid solution through the occupancy of dopants is relatively easier as the five different Ca^2+^ environments in its structure offer flexibility to attain this task. The research investigations on a wide variety of elements ranging from monovalent, divalent and trivalent confirm the attainment of *β-*TCP solid solution. Depending on the size and valence, the intended element prefers to occupy the five different Ca^2+^ sites of *β-*TCP. Investigations on single Zn^2+^ (0.745 Å) or Mg^2+^ (0.650 Å) substitutions in *β-*Ca_3_(PO_4_)_2_ revealed their preferred occupancy at the Ca (5) site six-fold coordinated with O. The replacement of the larger Ca^2+^ (1.00 Å) with smaller ions resulted in contractions of unit cell parameters [[92], [93], [94]]. The preference of the smaller cations Zn^2+^ and Mg^2+^ for the Ca (5) site is explained by its shortest mean distances (2.238 Å and 2.287 Å) determined for the Ca (5)–O bonds in the structure, favouring their easy accommodation. The ability of the monovalent Na^+^ to enter the *β-*TCP structure has also been reported [95]. Table 2 illustrates the effects of the various ionic substitutions at the crystal lattices of HA and *β*-TCP. Further, the capacity of *β-*TCP structure to accommodate coupled ionic substitutions has also been investigated; however, the preferential occupancy of the ions depends on their size and valence. The coupled substitutions of Sr^2+^/Mg^2+^, Sr^2+^/Zn^2+^, and Sr^2+^/Cu^2+^ revealed the preferred occupancy of lower sized Mg^2+^, Zn^2+^, and Cu^2+^ at the Ca (5) site, while the larger Sr^2+^ (1.38 Å) was favourably accommodated at the Ca^2+^(1), Ca^2+^(2), Ca^2+^(3) sites [22,84,96].

**Table 2:** Effects of ionic substitutions at the crystal lattices of HA and β-TCP.

No	Ion	Size (Å)	Occupancy in HA		Refs.	Occupancy in *β*-TCP		Refs.
			Site Occupancy	Lattice Strain		Site Occupancy	Lattice Strain	
1.	Sr^2+^	1.38	Ca^2+^(1) and Ca^2+^(2) sites	Expansion	[97]	Ca^2+^(4) site	Expansion	[96]
2.	Mg^2+^	0.645	Least preferred	Contraction	[98]	Ca^2+^(5) site	Contraction	[93]
3.	Zn^2+^	0.742	Ca^2+^(2) site	Contraction	[99]	Ca^2+^(5) site	Contraction	[94]
4.	Na^+^	1.02	Ca^2+^(1) site	Expansion	[100]	Ca^2+^(4) site	*a*-axis expansion and *c*-axis contraction	[95]
5.	K^+^	1.31	Ca^2+^(1) site	Expansion	[100]	Ca^2+^(4) site	*a*-axis expansion and *c*-axis contraction	[101]
6.	Ag^+^	1.28	Ca^2+^(1) site	Expansion	[102]	Ca^2+^(4) site	*a*-axis expansion and *c*-axis contraction	[102]
7.	Cu^2+^	0.72	Ca^2+^(2) site	Contraction	[103]	Ca^2+^(5) site	Contraction	[22]
8.	Gd^3+^	0.938	Ca^2+^(2) site	Contraction	[104]	Ca^2+^(1), Ca^2+^(2) and Ca^2+^(3) sites	*a*-axis expansion and *c*-axis contraction	[104]
9.	Dy^3+^	0.912	Ca^2+^(2) site	Contraction	[105]	Ca^2+^(1), Ca^2+^(2) and Ca^2+^(3) sites	*a*-axis expansion and *c*-axis contraction	[105]
10.	CO_3_^2–^	–	Either A type (substitution at OH^−^ site), B type (substitution at PO_4_^3−^ site) or Mixed AB type.	Negligible	[66,106]	Negligible	Negligible	[66,106]
11.	F^−^	1.30	OH^−^ site	Negligible		Negligible	Negligible	
12.	Cl^−^	1.80	OH^−^ site	Negligible		Negligible	Negligible	

Other than monovalent and divalent ionic substitutions, the single, double and triple substitution of trivalent lanthanides (Gd, Dy, Yb) have been also reported [104,105,[107], [108], [109]]. It has been shown that the critical limit to retain *β-*TCP solid solution is established as 4.35 mol%, 2.2 mol %, and 1.25 mol% for the single, coupled, and triple elemental substitutions, respectively. Beyond the substitution limit, the excess cations crystallise as additional lanthanide phosphate or oxide phases. Gd^3+^ (1.05 Å) and Dy^3+^ (1.02 Å) preferred their occupancy at the Ca^2+^(1), Ca^2+^(2), Ca^2+^(3) sites, while the lower sized Yb^3+^ (0.87 Å) enters at the Ca^2+^(5) site of the *β-*TCP structure.

### 2.5 Composites based on CaPs

Composites comprising bioactive CaPs and bioinert ceramics have been extensively investigated for hard tissue replacements [97,98]. HA/Al_2_O_3_ and HA/ZrO_2_ are intensely explored, since they could harmoniously intertwine the bioactivity of HA with the mechanical strength of Al_2_O_3_/ZrO_2_ components. Nonetheless, the shortcomings of these composites are significant, mainly stemming from the almost ubiquitous formation of undesirable secondary phases during sintering. In HA/Al_2_O_3_ composites, the free CaO from HA easily interacts with Al_2_O_3_ to yield CaAl_2_O_4_ and consequently the structural stability of the resultant composite is not maintained [99,100]. In a similar manner, CaO readily reacts with ZrO_2_ component of HA/ZrO_2_ composites to yield calcium zirconate [[101], [102], [103]]. The decomposition mechanisms of HA/Al_2_O_3_ and HA/ZrO_2_ composites are explained below (Eq. (eq. 10), (eq. 11), (eq. 12), (eq. 13))).

(eq. 10) Ca5PO43OH→Ca5PO43OH1−yOy2+y2H2O

(eq. 11) 2Ca5PO43OH1−yOy2+Al2O3→3Ca3PO42+CaAl2O4+1−yH2O

(eq. 12) $${\mathrm{Ca}}_{10}{\left({\mathrm{PO}}_{4}\right)}_{6}{\left(\mathrm{OH}\right)}_{2}\;\to \;{\mathrm{Ca}}_{10}{\left({\mathrm{PO}}_{4}\right)}_{6}{\left(\mathrm{OH}\right)}_{2-2x}O_{x}+{\mathrm{xH}}_{2}O$$

(eq. 13) $${\mathrm{Ca}}_{10}{\left({\mathrm{PO}}_{4}\right)}_{6}{\left(\mathrm{OH}\right)}_{2-2x}O_{x}+{\mathrm{yZrO}}_{2}\left(\mathrm{tetragonal}\right)\to \;{\mathrm{Ca}}_{3}{\left({\mathrm{PO}}_{4}\right)}_{2}+\mathrm{CaO}{\left({\mathrm{ZrO}}_{2}\left(\mathrm{cubic}\right)\right)}_{y}+\left(1-x\right)H_{2}O$$

The attempt to develop alternative composites based on HA/TiO_2_ and *β*-TCP/TiO_2_ combinations [106,110] also led to the thermal decomposition in accordance to the below illustrated mechanisms (Eqs. (eq. 14), (eq. 15))).

(eq. 14) $${\mathrm{Ca}}_{10}{\left({\mathrm{PO}}_{4}\right)}_{6}{\left(\mathrm{OH}\right)}_{2}+{\mathrm{TiO}}_{2}\to 3{\mathrm{Ca}}_{3}{\left({\mathrm{PO}}_{4}\right)}_{2}+{\mathrm{CaTiO}}_{3}+H_{2}O↑$$

(eq. 15) $${\mathrm{Ca}}_{3}{\left({\mathrm{PO}}_{4}\right)}_{2}\;{+\mathrm{TiO}}_{2}\;\to {\mathrm{CaTiO}}_{3}+{\mathrm{\alpha Ca}}_{2}P_{2}O_{7}$$

The major drawback in the development of composites based on HA is the presence of the structural hydroxyl component that triggers dehydroxylation and, consequently, results in failure. As an alternative, the selection of *β-*TCP ensures the structural stability of composites at elevated temperatures. The reason being the occupancy of dopants at the crystal lattice of *β-*TCP until their saturation limit beyond which these dopants oxidize at elevated temperature to yield composites. This strategy has been used to form *β-*TCP-based composites with different metal oxides (*i.e.*, *c*-CeO_2_, *α*-Al_2_O_3_, *α*-Fe_2_O_3_, *r*-TiO_2_, *t*-ZrO_2_, and ZnO [[111], [112], [113], [114], [115], [116], [117], [118]]). A minor adjustment in the precursor concentrations enables the formation of assorted levels of individual component ratios in the composites. *β-*TCP/*c*-CeO_2_ and *β-*TCP/*α*-Al_2_O_3_ maintain their structural stability until 1400 °C, while *β-*TCP/*α*-Fe_2_O_3_, *β-*TCP/*r*-TiO_2_ and *β-*TCP/ZnO composites retain their stability until 1300 °C. Moreover, the occupancy of Ce^3+^, Ti^4+^, Zn^2+^, Al^3+^, and Fe^3+^ at the *β-*TCP lattice sites delays its conversion at a higher temperature than the regular one (*i.e.*, 1180 °C) of stoichiometric *β-*TCP. The investigated composites have also revealed better mechanical properties than the stoichiometric *β-*TCP and the natural bone.

### 2.6 Fabrication of CaP based scaffolds

**Fig. 4:**
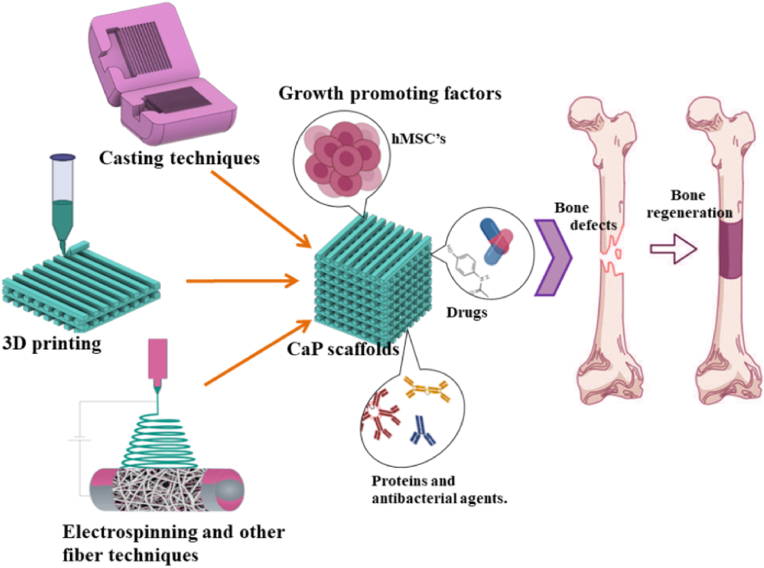
Fabrication techniques involved in the development of scaffolds for BTE.

The superior biocompatibility, bioactivity, and biodegradability of CaP ceramics are highly valued especially in the design of scaffolds for bone tissue engineering (BTE) [119]. Generally, the scaffold design for BTE applications demands suitable porous structures, which enable the effective transport of nutrients throughout the scaffold alongside the elimination of waste (Fig. 4). Scaffold fabrication for BTE can be accomplished through a variety of methods; however, the optimisation of scaffold manufacturing techniques turns out to be crucial, preventing the creation of fragments during their *in vitro* or *in vivo* evaluation [120]. Slip-casting technique demonstrated the potential to manufacture HA scaffolds with porosity beyond 50 % alongside pore sizes up to 750 μm; nevertheless, the impact of sintering temperature has laid a significant impact on the pore size distribution of the resultant scaffold [121]. A porous scaffold achieved by employing polymer replica method on silicocarnotite-tricalcium phosphate envisaged eutectoid microstructures during their immersion in phosphate buffer saline (PBS) that caused the dissolution of silicocarnotite phase to establish an interconnected microporous apatite structure *via* the pseudo-morphic transformation of the *α*-TCP phase [122]. Calcium silicate/HA nanofiber scaffolds realized through electrospinning and freeze drying exhibited rapid degradation alongside the tuneable release rate of Si^4+^ and PO_4_^3−^, which consequently aided in the bone regeneration and also countered the drawback of slow degradation ability of HA [123]. ACP and polylactide nano-fibrous scaffolds developed through electrospinning endorsed high degradation rate and enhanced osteogenesis through sustained release of Ca^2+^ and PO_4_^3−^ [124]. Patterned scaffolds comprising *β*-TCP and calcium pyrophosphate constructed through chemical etching technique displayed ridges and grooves in the range of 900 nm–1.5 μm. Further, the ceramics volume fraction and the etching time had influenced the role of topographical modification in enhancing the competency of osteogenesis [125]. Scaffolds obtained from natural cuttlefish comprising HA and *β*-TCP in various ratios displayed more than 90 % porosity together with enhanced compressive strength of ∼2.4 MPa that complemented the trabecular bone [126]. The methods adopted through uniaxial pressing, slip-casting, and starch consolidation to yield HA scaffolds, exhibited better mechanical strength but lower flexibility in tailoring the porous structure of the scaffolds [121].

The shortcomings of the negligence to attain desired and uniform porosity throughout the scaffold architecture, consumption of excess time and laborious work force by the conventional techniques has led to the shifting trend towards additive manufacturing (AM) techniques, which displayed the ease to overcome the limitations of the former. AM techniques in BTE enable defect free scaffolds, printed with high precision, consumption of minimum time and low production cost. AM also aids in tuning the number of factors, which thus yield a scaffold with perfect architecture. Nevertheless, depending on the requirement to yield a unique scaffold with desired features for a patient specific demand, it becomes imperative to figure out the best among the various available AM strategies [127]. For the readers’ benefit, the most significant approaches implemented to achieve 3D scaffolds with improved performance are summarized in Table 3.

**Table 3:** Fabrication of CaP-based 3D scaffolds by additive manufacturing.

Source material/type	CaP role	Main functionality/Advancements	3D printing technology	Refs.
HA	Coating on 3D printed FE scaffold	● Enhanced alkaline phosphatase activity; ● Improved osteogenic differentiation.	Direct ink writing	[128]
BCP	Doped with Sr and Ag	● Better inter-connected pores in the range of 50–70 %	Direct Ink Writing	[74]
CaP–Se composite	Coating on 3D printed PCL scaffold	● Antibacterial efficacy; ● Improved tissue regeneration.	Screw-extrusion Printing	[26]
Mg–Sr–Cu doped β-TCP	Scaffold	● Reduction in micro-cracks; ● Enhanced compressive strength.	Direct Ink Writing	[129]
Sr and Sr/Mg BCP	Scaffold	● Mg played a destabilizer role on HA; ● Mg and Sr acted as stabilizer for β-TCP.	Robocasting	[76]
CaP	Reinforced CaP scaffolds using *Luffa* fibres	● Interconnected channels mimicking the bone architecture.	Sacrificial Template Method	[130]
CaP slurry	CaP scaffolds	● Reduction in viscosity through addition of camphor; ● 50 % solid loading was achieved and elevated compressive strengths.	Direct Light Processing	[131]
BCP/chitosan	3D scaffolds	● Sintering-free scaffolds; ● High solid loading without binders; ● Drug loading capability.	Robocasting	[132]
CaP	Scaffolds crosslinked *via* sodium alginate	● Anti-bacterial efficacy; ● Bone regrowth functions.	Stereolithography	[133]
CaP	Coatings on 3D printed Ti6Al4V scaffolds	● High surface roughness; ● Uniform bone formation.	Selective Laser Melting	[134]
Sr-doped CaP	Coatings on 3D printed Ti6Al4V scaffolds	● Hastened bone healing.	Selective Laser Melting	[135]
*β*-TCP/*α*-Al_2_O_3_ composite	Scaffold with PLA as matrix	● Enhanced tensile, compressive and flexural strength.	Fused Deposition Modelling	[136]
CaP	Collagen as binder for CaP scaffold	● Augmented flexural strength and cell viability.	Ink Jet Printing	[137]

### 2.7 Injectable bone cements based on CaPs

CaP-based bioceramics for bone defects repair are usually implanted in the form of granules or precisely-sized blocks. Nevertheless, the basic knowledge on the nature and size of the specific bone defect to be repaired is essential in terms of devising an appropriate methodology to manufacture a determined sized scaffold to precisely fit into the defective site [138,139]. In this context, injectable calcium phosphate bone cements (CPC) are preferred to rectify a bone defect. The intrinsic characteristics of CPC, namely their injectability and capability of setting in *in vivo* environment, offers the likelihood of mild invasive operative techniques that restrain the need for open surgery, reduce patient distress, minimizing infection, scar formation and finally reduces the treatment cost. Further, CPCs also provide the option of appropriate defect filling, implant fixation and ease of handling [138]. Despite these advantages, CPCs also possess a series of shortcomings such as their possible degradation in physiological conditions, lack of appropriate porosity and weak mechanical compatibility [[139], [140], [141]]. Notwithstanding these shortcomings, the positive physical and chemical characteristics, namely the bioactivity and good absorbing capacity of biomolecules, facilitate their combination with growth factors, drugs and polymers [[142], [143], [144], [145], [146]]. All these features have showcased CPCs as effective bone graft options. The new injectable CaP formulations comprised of mixtures of tetra-calcium phosphate (TTCP), dicalcium phosphate dihydrate (CaHPO_4_ · 2H_2_O, DCPD, brushite) and anhydrous dicalcium phosphate (CaHPO_4_, DCPA, monetite) were proposed [147]. These cements are reported to exhibit good injectability, excellent self-setting ability, enhanced reactivity and high feasibility that enabled the design of new drug delivery systems.

CPC integrated with different polymers, namely, polylactic-glycolic acid (PLGA), gelatine and polytrimethylene carbonate (PTMC), injected into the defective femoral head of a rabbit model, ensured better response and offered good degradation behaviour of CPC/PLGA cement among the investigated counterparts [148]. The *in vivo* performance of biphasic synthetic bone graft material, constituted of CaSO_4_ and *β*-TCP, and injected into a defective sheep bone model, revealed new bone formation with simultaneous material resorption in 8 weeks of implantation, as reported by Yang et al. [149]. Verron et al. reported the implantation of CaP cement mixed with an anti-osteoporotic drug ensuring better support to the trabecular bone of a sheep [150].

The last two decades have witnessed the combination of CPC with polymers and additives like citric acid in the attempt to improve the consistency and injectability of the bone cement [151,152]. Synthetic and natural polymers have been utilized in an aqueous form to accomplish good injectability and better cohesion of the CPC without compromising the setting time and mechanical strength [119,146]. Chitosan, a natural polymer is utilized in liquid form with an aim to tailor the physical characteristics of CPC such as injectability, setting time, and rheological parameters for ensuring a superior *in vivo* performance [153]. Solutions of collagen [154,155], gelatine [156,157], hyaluronic acid [155,158], sodium alginate [159,160], and cellulose derivatives [147,155,161,162] have been also used as liquid media to facilitate the flowability of the pastes and enhance the functional properties of CPC. The use of biopolymers has ensured the cohesiveness and injectability of CPC together with improved mechanical features.

**Fig. 5:**
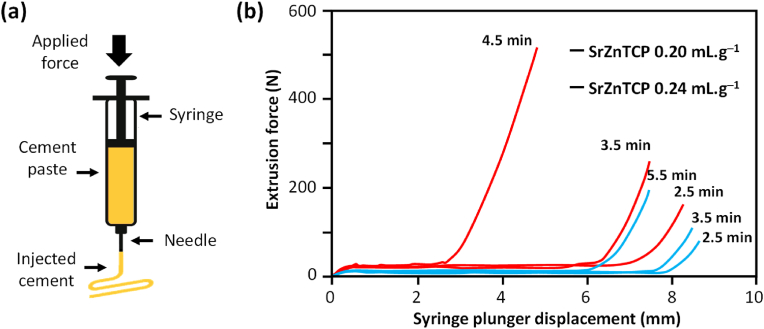
(a) Schematics of the syringe under the extrusion process; (b) Extrusion force versus syringe plunger displacement curves registered at different time points after cement paste preparation. Results subsidiary to research published within Refs. [166,167].

Alongside the use of polymer and additive combinations, attempts were also made to develop ion-substituted CPCs aiming to improve the overall properties of the cements. Sr-substituted *α*-TCP cement triggered a strong lattice strain to the CaP structure, which consequently improved the milling behaviour and enhanced the reactivity of the resultant cement [163]. The use of citric acid solution as a setting liquid in *α*-TCP and Mg-substituted *α*-TCP has been found effective in terms of achieving high reactivity of the cement powders. Nevertheless, the maximum effectiveness of citric acid revealed to be dependent on its optimal concentration (15 wt%), beyond which the reactivity of the powder showed a decreasing trend [164]. A study on the effects of particle size, particle size distribution, morphology, and state of aggregation of the Sr-substituted CaP-based cements on their injectability has been performed by Torres et al. [165]. The significance and interdependent roles of the various powder features and ionic strength in the liquid media, which consequently determine the flow and injectability behaviour, were emphasized. Zn- and Sr-substituted brushite cements could be easily injected using liquid to powder ratio (LPR) values less than half in comparison to a commercial carbonated apatite cement (Norian SRS®, LPR = 0.5 mL g^−1^) used as control, according to the extrusion force versus syringe plunger displacement curves displayed in Fig. 5b, registered at different time points after cement paste preparation. These Zn- and Sr-substituted brushite cements revealed their apatite transformation under physiological conditions, and were reported to be non-cytotoxic to human osteosarcoma derived MG-63 cells [166].

**Fig. 6:**
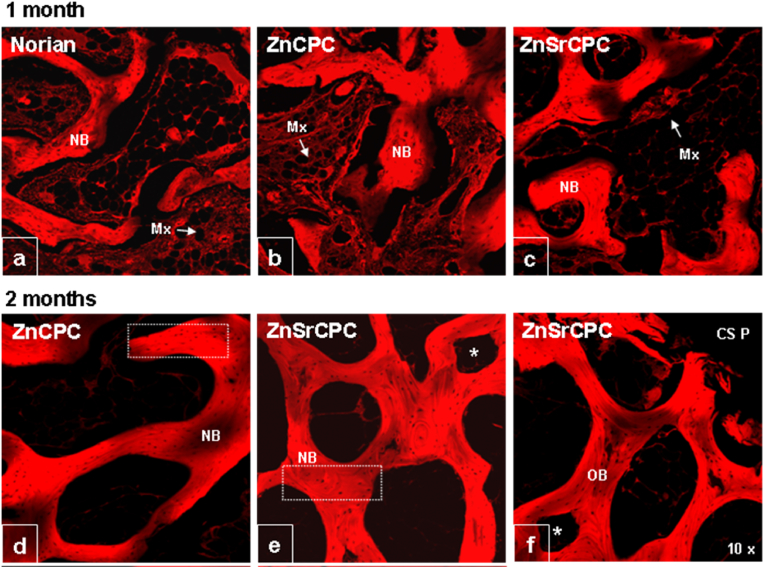
Confocal fluorescent micrographs of histological H&E stained sections, showing the tissue osteogenic response to (a) Norian SRS®, (b,d,f) ZnCPC and (c,e,g) ZnSrCPC cements after (a–c) one and (d–g) two months of implantation. NB: new bone; Mx: protein matrix. Adapted with permission from Ref. [167].

Moreover, both the ZnCPC and the ZnSrCPC presented better *in vivo* performance in comparison to the control carbonated apatite cement (Norian SRS®) [167] as shown in Fig. 6. The presence of Sr enhanced the rate of cement resorption and new bone formation. The same cements were also *in vivo* tested in rat model by our collaborators from the Faculty of Dentary Medicine of University of Porto, Portugal, in the frame of their post-graduation Master theses, with similar outputs.

Furthermore, amazing effects on setting time, injectability, and cement-MG63 osteoblast cells interactions were well documented when comparing different cement samples prepared from powders of pure β-TCP (Ref-TCP); 5 mol% Sr-doped β-TCP; β-TCP co-doped with 5 mol% Sr and with 0.5–1 mol% Mn (Mn0·5Sr5CPC), and (Mn1Sr5CPC) [168].

The combined effects of ion doping with different setting liquids were also investigated, using a 15 wt% citric acid solution + 10 wt% poly (ethylene glycol-PEG) + 0.5 wt% hydroxyl propyl methylcellulose (HPMC), (Liquid A, absence of sugars), with added 10 wt% sucrose (Liquid AS), or 10 wt% fructose (Liquid AF) as setting retardants for cement pastes with LPR = 0.28 mL g^−1^, extruded 2.5 min after mixing the powders with the setting liquids.

**Fig. 7:**
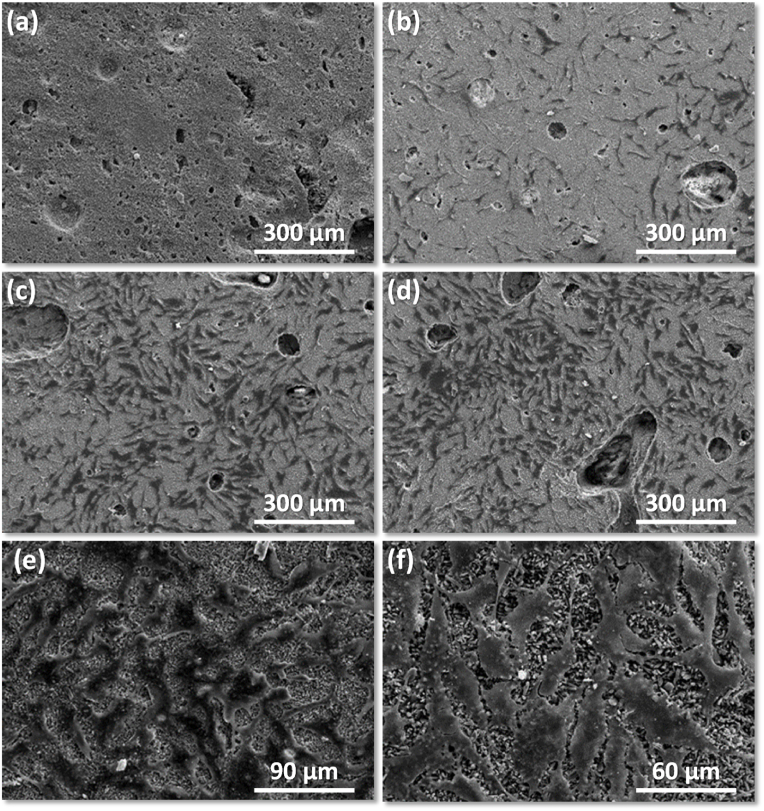
Representative aspects of the interactions between the MG63 cells cultured for 7 days on the following cement disc substrates: (a) Ref CPC-A; (b) Sr5CPC-A; (c) Mn0·5Sr5CPC-A; (d) Mn0·5Sr5CPC-AF; (e–f) Mn0·5Sr5CPC-AS. Results subsidiary to research published within Ref. [168].

It was shown that under those conditions, Ref-CPC did not extrude; the presence of sugars considerably facilitated the injectability of 5 mol% Sr-doped β-TCP, while further co-doping with small amounts of Mn dramatically enhanced injectability. Further, dramatic effects of co-doping and adding sugar retardants were observed on cells adhesion behaviours, as illustrated in the SEM images displayed in Fig. 7.

Seemingly, cells did not adhere to the Ref CPC-A substrate. Low surface cell density could be observed on the Sr5CPC-A disk. Further, co-doping with a very small amount of Mn considerably increased the affinity of cells toward the Mn0·5Sr5CPC-A substrate. The combination of co-doping with the addition of sugars further synergistically enhanced the cells adhesion as observed for the Mn0.5Sr5CPC-AF and Mn0.5Sr5CPC-AS samples.

Aiming at developing long shelf-life and ready to use injectable CPC formulations, and shorten the chirurgical intervention times, additional attempts have been made by replacing the reactive aqueous solutions by non-reactive liquids such as glycerol (G) [169], polyethylene glycol (PEG) [170], and foaming agents to create porosity in the hardened cements [171]. The hydraulic cure would occur after injecting the paste into bone defects when placed in contact with physiological fluid. Besides CaP-powders, the dispersed inorganic components used to prepare flowable pastes could also include strontium carbonate [172], and β-dicalcium silicate to adjust the physico-chemical and biological properties of CPCs [173]. These investigations proved to be promising, especially, and improved ability for adjusting the rheological behaviour and the injectability, in comparison to aqueous based ones, enabling the delineation of CPC formulations more adequate for specific targeted orthopaedic clinical applications.

## 3 Advances in bioactive glasses

### 3.1 Glasses: their nature, structure and chemistry

Glasses are a unique class of materials used by mankind for thousands of years in numerous forms and applications. Their uses range from simple common materials (*e.g.*, windows, bottles, containers, light bulbs, *etc*) to high-tech applications in diverse fields ranging from computer screens, spectacles and telescope lenses, spectrometer prisms, laboratory ware, optical fibres, electronic, military and medical applications, *etc* [[174], [175], [176]].

The glasses are generally prepared by two primary methods, namely by the melt-quenching and the sol-gel processes. In the melt-quenching process, the batch comprising the precursors is heated at relatively high temperatures and then quenched to freeze the disordered atomic structure of the melt and get amorphous materials. The quenching can occur by pouring the melt in a metallic or graphite mould to prepare shaped bulk glass (*e.g.*, rod or cylinder), after which in most cases the glass is heat-treated at lower temperatures in order to relax the thermo-mechanical stresses in the structure induced by rapid cooling, a procedure known as annealing. If the melt is rapidly cooled down (*e.g.*, by pouring it directly into water) it will be possible to produce a glass frit that can be dried and milled to get glass powders with specific particle size distributions. On the other hand, the sol-gel process uses inorganic/organic precursors subjected to different processes of hydrolysis and condensation, followed by drying and thermal stabilisation heat-treatments. By such a process, the final glass product can be shaped into nanoparticles, coarser powders, fibres, coatings, *etc*.

Among other contrasts, glasses differ from crystalline materials by exhibiting a glass transition temperature (*T*_*g*_). More correctly, *T*_*g*_ is not a specific temperature but an interval of temperatures that depends significantly on the composition and thermal history of the material (*e.g.*, melting temperature, cooling rate, and the subsequent heat-treatment schedules). In this interval of temperatures, the solid glass becomes a viscous liquid glass [175].

One of the great advantages of glasses in comparison to crystalline materials resides in their compositional flexibility. In opposition to crystalline phases, which are highly dependent on specific and constant stoichiometries, glasses can be synthesized from practically unlimited variety of compositions that can be designed to adjust to a specific necessity [177], and their properties can be engineered by adding designed amounts of other oxides to the composition. This is particularly important in the compositional design of bioactive glasses (BGs) since it can allow the incorporation and the release of potential therapeutic actions (*e.g.*, osteoinduction, neuroprotection, antimicrobial effects) upon their *in vitro* and *in vivo* degradation [178].

Contrarily to crystalline solids, glasses do not show any long-range order or any significant symmetry in their atomic arrangement [175]. Instead, the atoms constituting glasses are organised in a short-range order that depends intimately on their composition. Glass seems to be a liquid, but its behaviour is similar to a solid when using human time scales [175]. However, for much larger time scales it is relaxing towards the supercooled liquid state. This means that the glass structure is unstable with respect to the supercooled liquid state, and the supercooled liquid is metastable with respect to the equilibrium crystal [179,180].

The structure of glasses has a direct effect on many important properties, such as glass stability, density, coefficient of thermal expansion (CTE), solubility, and ion release in aqueous environments. The eventual occurrence of surface modifications upon reacting with natural or synthetic physiological fluids is regarded as an indication for the glass mineralization capacity.

Glasses are commonly formed by different oxides, which play specific roles in the glass processing, structure, and properties. Depending on the type of structural function, the components in oxide glasses are divided into three main groups (1) *Network formers* (*e.g.*, SiO_2_, B_2_O_3_, and P_2_O_5_) – components that build the glass network by forming oxygen tetrahedra and oxygen triangles, which are also called network units or structural units. The network formers are the essential components of glasses, being able to form 3D structures; (2) *Network modifiers* (*e.g.*, Li_2_O, Na_2_O, K_2_O, CaO) – components that break down the glass network by creating terminal oxygens; and (3) *Intermediate oxides* (*e.g.*, Al_2_O_3_, MgO) – components that assume either the role of network formers or network modifiers, depending on the specific glass composition. It is worth to note that Al_2_O_3_ is often not desirable in the composition of BGs in contents greater than 1.5 wt% because it tends to turn the glasses bioinert, inhibiting bone bonding ability [181].

The glass structural units are linked to each other by corner-shared oxygen bridges (BO). The presence of network modifiers, which act as fluxing agents, decrease considerably the melting temperatures of the glasses, reducing the production costs. This is due to their effect on promoting the disruption of the glass network, cleavage of BOs, and formation of non-bridging oxygens (NBO).

Glasses can be classified according to their compositional systems, usually taking into consideration the network former oxide(s) that are present in the composition. Therefore, glasses can contain just one network former oxide (such as silicate, phosphate, and borate glasses) or consist of more complex compositions with mixed glass network former oxides (such as borosilicate, phosphosilicate, borophosphate glasses, *etc*) [182]. For example, common window glass is usually based on the soda–lime–silica (Na_2_O–CaO–SiO_2_) system [183], whilst in traditional BGs, the base components are SiO_2_, Na_2_O, CaO, and P_2_O_5_ [184].

### 3.2 Glasses as bioactive materials

The need to find novel materials that could form a strong bond with living tissues and overcome the body's rejection of inert metal and plastic implants used in soldiers injured during the Vietnam War was the driving force for the discovery of BGs by Larry Hench in the late 1960s [185]. The bonding ability of a glass to bone and muscle after six weeks post-implantation in rats was firstly observed for the composition 45SiO_2_–24.5Na_2_O–24.5CaO–6P_2_O_5_ (wt%), which was later named as 45S5 Bioglass® [186,187]. When glasses in the Na_2_O–CaO–P_2_O_5_–SiO_2_ system are immersed in biological fluids, a layer of hydroxyl carbonated apatite (HCA) is formed on their surface. The bone-bonding ability is conferred by this HCA layer, which is chemically and structurally similar to the mineral apatite phase found in bone tissue.

BGs commonly exhibit higher rates of HCA formation, bone-bonding ability and higher osteogenic capacity than bioactive ceramics [181]. Also, BGs undergo degradation over time when in contact with body fluids, stimulating tissue regeneration and being gradually replaced by new bone formation. The scientific community was thrilled after discovering that BGs were the first artificial materials with demonstrated ability to form an integrated bond with not only bone, but also soft tissues [187]. Consequently, these materials have gained great interest amongst the biomedical community.

However, 45S5 Bioglass® composition features several drawbacks, most of them related to its high alkali content, such as: (*i*) relatively fast dissolution and resorption rates that negatively affect the balance of natural bone remodelling [188], and lead to gap formation between the tissue and the implant material [189]; (*ii)* narrow sintering window (Δ*T* = *T*_*c*_−*T*_*g*_), which hinders the densification, resulting in weak mechanical strength and early crystallization [[190], [191], [192], [193]]. This represents a serious limitation when envisaging the manufacture of macro-porous scaffolds; (*iii*) high coefficient of thermal expansion, which makes difficult its application as adherent coating material for metallic, polymeric, or ceramic implants [[194], [195], [196], [197]]; and (*iv*) high pH generated by the high doses of leached sodium, causing cytotoxic effects [198,199]. An increased concentration of Na_2_O at the expense of MgO induced cytotoxicity towards the mouse-derived pre-osteoblastic MC3T3-E1 cell line and a delayed formation of HCA surface layer in the simulated body fluid (SBF) solution [199]. Also, a slight depolymerization trend in the silicate glass network was observed, accompanied by an enhanced affinity of alkali cations towards phosphate [199].

Since the discovery of 45S5 Bioglass® and its proved bone-bonding ability potential, many efforts have been focused to develop new glass compositions that could hinder or reduce the cytotoxicity response. For instance, the cytotoxicity response of polycaprolactone (PCL)-45S5 Bioglass® composite scaffolds was mitigated after a prolonged immersion in water, which suppressed the development of HCA *in vitro*, with calcite being preferentially formed [200]. Fabbri et al. [201] also prepared polycaprolactone (PCL)-45S5 Bioglass® composites with BG weight contents varying in the range 0–50 %. The ability of the composites to induce the precipitation of HCA increased with the 45S5 Bioglass® content. The non-optimal *in vitro* performances in terms of cytotoxicity and osteoblast proliferation were attributed to the poor wettability of the composites, but the effects of sodium leaching could not be discarded.

Meng et al. [202] developed 45S5 Bioglass®-based scaffolds loaded with tetracycline microspheres for drug delivery in bone tissue engineering. They concluded that the constructs exerted limited cytotoxicity in mouse fibroblast cells. Ball et al. [203] performed cytotoxicity assessments using mouse osteoblasts cultured directly on porous scaffolds made of ceria and of 45S5 Bioglass® for 72 h, and determined that the cytotoxicity of ceria was rather lower in comparison to that exerted by 45S5 Bioglass®. Zhang et al. [204] compared the cytocompatibility of an apatite–wollastonite (A-W) bioactive glass-ceramic prepared by the sol-gel method with that of a 45S5 Bioglass® prepared by the melt-quenching method and evaluated the viability of bone marrow stromal cells (BMSCs) cultivated in presence of the extracts of the two kinds of biomaterials. The A-W material exhibited superior cell proliferation in comparison to 45S5 Bioglass®.

Piezoelectric ceramics based on barium titanate (BT) are currently explored in orthopaedic research for the electrical stimulation of bone-forming cells. The *in vitro* biocompatibility of porous scaffolds made of BT and of 45S5 Bioglass® was comparatively tested for 72 h in mouse osteoblast (7F2) cell cultures [205]. The cytotoxicity of 45S5 Bioglass® was found higher with respect to that of BT. This inferior *in vitro* performance of 45S5 Bioglass® is highly concerning, considering the barium muscle poison effect that is expected from the dissolution of non-stoichiometric BT [206]. Besides an increase in pH due to the ionic exchange owned to BT dissolution, the leached Ba^2+^ could cause added toxic effects, since they can directly stimulate all types of muscles, including cardiac muscle, and cause a profound reduction in serum potassium together with an increase in intracellular potassium [207].

Aiming at reducing the cytotoxicity effects of 45S5 Bioglass®, Pryce and Hench [208] tested a pre-conditioning step in melt-quenched derived 45S5 Bioglass® and sol-gel synthesized 58S compositions to reduce the fast ion exchange reaction and the associated pH rise in the culture medium that causes cytotoxicity. The two glasses were immersed in SBF for periods ranging from 30 min to 48 h and the effects on glass dissolution and subsequent HCA formation were evaluated. The results showed that the sodium release was diminished and no significant changes in the HCA layer formation rate were observed, concluding that the pre-conditioning step does not adversely influence the bioactivity. However, this pre-conditioning step cannot be viewed as being feasible in the surgery room.

The cytotoxicity effects are also common to other 45S5 Bioglass®-derived glass compositions [209]. Besides the cytotoxicity effects, the 45S5 Bioglass®-based porous scaffolds also feature poor overall mechanical performance, which would make them unsuitable for complex clinical applications [193,[210], [211], [212], [213], [214]]. Improving the mechanical properties of the scaffolds without radically altering their bioactivity, degradability, surface energy or wettability, while enabling drug-release capabilities, are much desired and anticipated in the near future.

### 3.3 Necessity of alkali-free bioactive glasses (AFBGs)

Since the discovery of BGs, most of the explored compositions include high alkali contents [215], aiming at decreasing the melting temperatures [216] and increasing the degradation of the silicate network over time [217,218]. However, the leaching of high alkali contents induces *in vitro* cytotoxicity effects in cell culture media and in the living tissues around the implant due to the high local pH environment [188,198,199,219,220]. Such high pH environment favours the formation of HCA, but is likely to give false positive mineralization (apatite-forming ability) results in SBF, while being unfavourable for homeostasis [198]. Excessive changes in the medium pH can inhibit osteoblast activity and cause cell necrosis or apoptosis [221].

The high alkali content BGs are usually also hygroscopic [222], which is a serious disadvantage for applications in BG/polymer composites, affecting the stability, degradation, and mechanical performance of the composite materials, while the presence of [OH^−^] ions on the surface of the glass powders promotes their crystallization.

The ideal BG should feature some important *in vitro* and *in vivo* performances, adequate thermal and physico-chemical properties, as well as processing ability [223]. The relevant features include absence of cytotoxic effects, biocompatibility, fast biomineralization rate *in vitro* with the formation of an HCA layer, osteogenic capacity, lack of genotoxic effects, good feasibility for scaffold fabrication by additive manufacturing techniques, ability to release therapeutic and anti-infection ions, and good matching of the coefficients of thermal expansion with metallic substrates for implant coating applications. The synthesis of BG materials comprising properties that satisfy all these requirements is a complex task, which demands innovative and smart approaches, motivating great interest on this subject and leading to important and continuous research activities in this field [30,218,[224], [225], [226], [227], [228], [229], [230], [231], [232], [233], [234], [235], [236], [237], [238]]. However, only a few review papers briefly referring to alkali-free BG compositions prepared by melt-quenching were published so far [[239], [240], [241]].

#### 3.3.1 AFBG systems

In recent years, several works developed and published by Ferreira and collaborators [195,196,199,219,220,223,[242], [243], [244], [245], [246], [247], [248], [249], [250], [251], [252], [253], [254], [255], [256], [257], [258], [259], [260], [261], [262], [263], [264]] have demonstrated that the most relevant features required for BGs can be obtained by reducing and even excluding the alkalis from glass compositions by using appropriate combinations of all the remaining important glass-forming components. The authors prepared several alkali-free bioactive glasses based on specific compositions of minerals that are biocompatible and bioactive (*e.g.*, tricalcium phosphate, fluorapatite, diopside and wollastonite) in different combinations and proportions. This concept and approach to the design of BG compositions is completely different. Furthermore, highly relevant ions, from therapeutic standpoint, were incorporated into the structure of the most promising delineated alkali-free glass compositions, aiming to boost their bio-functionality and, in the same time, preserve or even improve their processability. A synopsis of the type and concentration range of such employed therapeutic ions along with the main structural, thermal, and biological effects, is given in Table 4, and further discussed in detail in the following subsections.

**Table 4:** Type and concentration range of therapeutic ions incorporated into alkali-free bioactive glasses and their effects on structure, thermal behaviour, and biological performance.

Therapeutic ion	Oxide type	Substituted oxide	Doping range (mol%)	Structural role	Thermal behaviour influence	Biological performance	Refs.
Sr^2+^	SrO	CaO	2–10	● Network modifier; ● Increase of Sr^2+/^Ca^2+^ ratio has not affected the glass structure.	● CTE of glasses had not elicited a linear variation with SrO content; ● T_g_ values remained practically constant.	● SrO incorporation retarded the apatite-forming ability of glasses in SBF; ● Chemical degradation of glasses in Tris–HCl decreased ∼ seven-fold by SrO substitution. ● Cytocompatibility in *human mesenchymal stem cell cultures*; **→**antibacterial efficacy against the *S. aureus* and *E. coli* strains.	[256,261]
Zn^2+^	ZnO	MgO	2–10	● Network intermediate. ● Increase of the Zn^2+/^Mg^2+^ ratio has not induced significant change of glass network connectivity.		● ZnO incorporation improved the chemical durability of glasses in Tris–HCl; ● Suppressed formation of carbonated-HA in SBF for high Zn^2+^ dosages; ● Cytocompatibility in *human mesenchymal stem cell cultures*; ● Antibacterial efficacy against the *S. aureus* and *E. coli* strains.	[256,262]
co-doping: Sr^2+^; Zn^2+^	SrO; ZnO	CaO; MgO	2–10; 2–10	● Sr^2+^ & Zn^2+^ co-doping expanded glass structure and weakened glass glass network.	● Sr^2+^& Zn^2+^ co-substitution allowed for CTE tailoring, matching it well to that of Ti-based alloys.	● Increase of the Zn^2+/^Mg^2+^ and Sr^2+/^Ca^2+^ ratios had not induced significant modification in the Q^n^ speciation and glass network connectivity; ● Chemical degradation in Tris–HCl was reduced, but without a negative influence on the apatite-forming ability in SBF; ● Antioxidant potential of glasses was revealed to be Zn- and Sr-dose dependent; ● Cytocompatibility in *MG-63 human osteosarcoma cells cell cultures*; ● Cytocompatibility in *human mesenchymal stem cell cultures*; ● Antibacterial effects against the *S. aureus* and *E. coli* strains were potentiated by Sr^2+^ & Zn^2+^ co-doping.	[250,251,256]
Cu^2+^ & Cu^1+^	CuO & Cu_2_O	CaO	1–5	● Network modifier. ● No remarkable glass structure changes were induced.	● Cu produced a decline of the T_g_.	● CuO incorporation enhanced the glass dissolution; ● Slightly decreased apatite-forming ability in SBF; ● *MG-63 human osteosarcoma cell viability* was reduced compared to the biological control; ● Cytotoxicity in *NIH/3T3 mouse fibroblast cell cultures* at doses of 50 mg/mL; ● Cytocompatibility in *NIH/3T3 mouse fibroblast cell cultures* at doses of 5 mg/mL. ● Antibacterial effect against the *S. aureus* strain at doses of 5 mg/mL.	[259,260]
Ga^3+^	Ga_2_O_3_	MgO	5	● Network former.	● Ga induced an increase of the T_g_.	● Cytocompatibility in *NIH/3T3 mouse fibroblast cell cultures* at doses of 5 and 50 mg/mL; ● Antibacterial effect against the *S. aureus* strain at doses of 5 mg/mL.	[259]
co-doping: Cu^2+^& Cu^1+^; Ga_2_O_3_	CuO & Cu_2_O; Ga_2_O_3_	CaO; MgO	2–3	● Cu played the role of network modifier; ● Ga played the role of network former.	● Cu and Ga had opposite influences, decreasing and increasing the T_g_, respectively, permitting to modify accordingly the glass processability.	● Cytotoxicity in *NIH/3T3 mouse fibroblast cell cultures* at doses of 50 mg/mL, owned to high Cu concentration release; ● Cytocompatibility in *NIH/3T3 mouse fibroblast cell cultures* at doses of 5 mg/mL; ● Antibacterial effects against the *S. aureus* strain were potentiated by Cu^2+^ & Ga^3+^ co-doping, when using glasses in doses of 5 mg/mL.	[259]
Co^3+^	Co_2_O_3_	CaO	1–5	● No remarkable glass structural changes were induced.	Not yet performed	● Co_2_O_3_ incorporation reduced apatite-forming ability in SBF; ● Doping levels of 3 and 5 mol% Co_2_O_3_ alleviated the oxidative damage mediated by H_2_O_2_; ● Cell viability in *MG-63 human osteosarcoma cell culture*s comparable to control only at the low doping levels (1 mol%).	[260]
Mn^2+^	MnO	CaO	1–5	● No remarkable glass structural changes were induced.	Not yet performed	● Good apatite-forming ability in SBF; ● Significant antioxidant ability at low dosages (1 mol%); this benefit was gradually lost and tended to be cancelled at the highest doping concentration (5 mol%). ● Cell viability in *MG-63 human osteosarcoma cell culture*s comparable to control only at the low doping levels (1 mol%).	[260]
Fe^3+^	Fe_2_O_3_	CaO	1–5	● Fe^3+^ doping increased glass network connectivity.	Not yet performed	● Fe_2_O_3_ incorporation improved chemical durability, but decreased apatite-forming ability in SBF; ● Cell viability in *MG-63 human osteosarcoma cell culture*s comparable to control only at the low doping levels (1 mol%); it was slightly reduced at higher dosages (3 and 5 mol%).	[260]

##### 3.3.1.1 Fluorapatite–diopside system

**Fig. 8:**
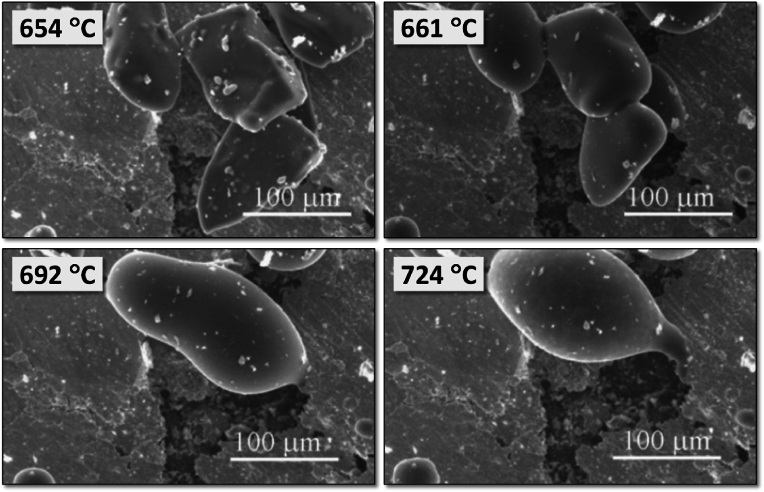
High-temperature environmental scanning electron microscopy (HT-ESEM) images of glass powder with mean particle size 220 μm obtained *in situ* during heat treatment of glass powder in the temperature range of 400–800 °C. Adapted with permission from Ref. [243].

Using this concept, a series of BG compositions within the fluorapatite [FA; Ca_5_(PO_4_)_3_F]–diopside (Di; CaMgSi_2_O_6_) system with varying FA/Di ratios [264,265] were synthesized by the melt-quenching, but amorphous glasses were obtained only for compositions up to 40 wt% of FA. All the investigated glasses featured Si predominantly present as *Q*^*2*^ (Si) species, while P was found in an orthophosphate *(Q*^*0*^*)*-type environment. The physico-chemical degradation tests by immersion in citric acid buffer revealed that all glasses exhibited weight gains instead of weight losses. Good cell viability and the considerable stimulation of osteoblastic differentiation suggested their possible use for bone regeneration [264]. Another important result was the significantly enhanced sintering ability and the apatite-forming capability of glasses/glass-ceramics with FA contents within 10–25 wt%. In particular, the BG composition 80Di–20FA, tested with different particle size distributions and mean particle sizes varying between 14 and 220 μm, exhibited good sintering ability irrespective of the mean particle size [243]. For the coarser particles, Fig. 8 shows that neck formation and other morphological changes were initially driven by surface diffusion at temperatures apparently below *T*_*g*_ but without evident macroscopic shrinkage.

With temperatures increasing above *T*_*g*_, the particles formed individual spherical droplets that merged into larger liquid droplets, which were obvious signs of their excellent sintering ability. Further increasing the temperature up to about 850 °C led to incipient formation of Di and FA crystals embedded into a high (∼90 wt%) residual glassy phase matrix, whose content tended to increase as the mean particle size increased.

##### 3.3.1.2 Diopside–fluorapatite–wollastonite ternary system

Based on the most interesting results gathered in the frame of previous works [243,264,265], another series of bioactive glass compositions were designed within the diopside–fluorapatite–wollastonite (W; CaSiO_3_) ternary system (80−*x*)[CaMgSi_2_O_6_]–20 [Ca_5_(PO_4_)_3_F]–*x* [CaSiO_3_], with *x* = 10–80 wt%, starting from the W-free parent glass composition, 80Di–20FA [219]. The aim of adding wollastonite was to further improve the sintering ability that was investigated by differential thermal analysis (DTA). The wider sintering window (∼145 °C) was observed within the range of W-10 to W-30, followed by a gradual narrowing trend with further increasing wollastonite contents. It was concluded that varying the CaO/MgO ratio of BGs did not exert any significant effect on their structure, with Si predominantly present in *Q*^*2*^ (Si) units and P found in an orthophosphate environment. Heat-treating glass powder compacts at 850 °C for 1 h resulted in well-sintered glass-ceramics with diopside, fluorapatite, wollastonite, and pseudo-wollastonite as the crystalline phases. Increasing the CaO/MgO ratio in these BGs reduced their sintering behaviour resulting in different amorphous/crystalline ratios in the obtained glass-ceramics. The glass-ceramics with compositions between W-10 and W-30 exhibited the higher amounts of residual glassy phase that favoured a strong apatite-forming ability.

##### 3.3.1.3 Diopside–fluorapatite–tricalcium phosphate system

**Fig. 9:**
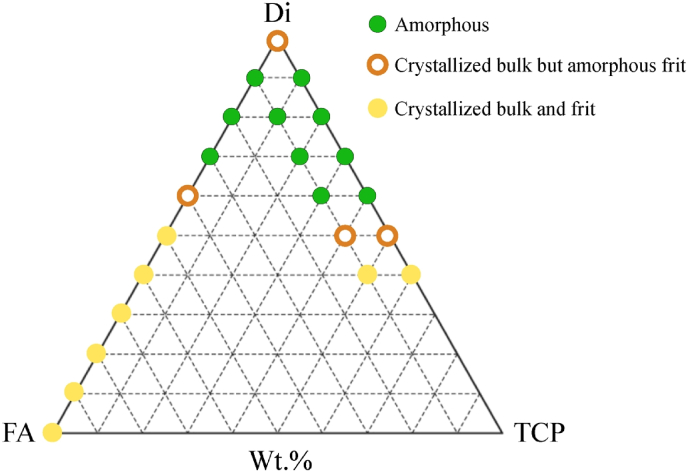
Graphical representation of the alkali-free BG compositions investigated [94,219,242,253,264,266].

Based on the same concept, new series of alkali-free BGs compositions were designed in the diopside (Di; CaMgSi_2_O_6_), fluorapatite (FA; Ca_5_(PO_4_)_3_F), and tricalcium phosphate (TCP; 3CaO·P_2_O_5_) system, combined in different proportions [94,219,242,253,264,266], and also in the binary Di-TCP and Di-FA systems. Fig. 9 shows some investigated compositions in the ternary Di–FA–TCP diagram system [243,264,265].

The glass-forming ability and stability of these glasses strongly depended on their specific compositions and on the cooling rate. FA-richer compositions were less prone to glass formation and tend to readily crystallise, even upon quenching the melts in cold water to obtain the glass frits. Some diopside-richer compositions allowed obtaining amorphous frits, but the bulk glasses tended to partially crystallise when cast on metal plates, especially in the parts further away from the metal plates that cooled more slowly, while others enabled obtaining amorphous materials. These last ones were the most interesting compositions from the processing viewpoint. The silicate network consisted predominantly of *Q*^*2*^ (Si) units, while phosphorus remained in an orthophosphate environment *(Q*^*0*^*)*, which is a common feature to all the designed alkali-free formulations. The effects of non-isothermal heating of on the structural changes, sintering ability, crystallization behaviour, and three-point bending strength of BGs in the binary system diopside (CaO·MgO·2SiO_2_)–tricalcium phosphate (3CaO·P_2_O_5_) with varying Di–TCP proportions were assessed [254]. Amorphous glasses could only be obtained from compositions with Di ≥ 50 wt%. Glasses with Di ≥ 60 wt% exhibit sintering temperature windows that are wide enough for the fabrication of scaffolds. Diopside and HA were the major phases present at 900 °C and 1000 °C with calcium silicate and *β*-TCP (whitlockite) as the minor phases. At 1200 °C, diopside and whitlockite were the major phases present, with no other minor phase detected.

Some of the investigated glasses exhibited HCA formation on their surface after immersion in SBF solution within the interval 1–12 h [219]. The composition 70Di–10FA–20TCP (*i.e.*, TCP-20) induced a particularly fast biomineralization capability with the formation of a crystalline surface HCA layer after immersion in SBF solution for 1 h. Since the bonding to living tissues after implantation is mediated by this HCA layer, a fast-bonding capacity is expected from these BG compositions, and especially from TCP-20, which was registered under the FastOs®BG trademark. The alkaline phosphatase activity and osteogenic differentiation using rat bone marrow mesenchymal stem cells seeded on sintered glass powder compacts revealed that the tested compositions are ideal potential candidates for applications in bone tissue engineering. FastOs®BG glass has demonstrated osteogenic activity, inducing the differentiation of human mesenchymal stem cells into bone-forming cells, even in the absence of osteogenic medium, and the osteoinduction effect was significantly superior in comparison to that of 45S5 Bioglass® [253]. When tested in animal model (sheep), the post-implantation histological and scanning electron microscopy assessments of retrieved subcutaneous and bone samples demonstrated that FastOs®BG is more slowly resorbed, more biocompatible and osteoconductive, and more easily osteointegrated in comparison to 45S5 Bioglass® [257]. These results have demonstrated that FastOs®BG has greater potential as bone graft material for large bone defects and will be further detailed in section 5 “Impact on clinical practice”.

#### 3.3.2 The enhanced processing ability of AFBGs

**Fig. 10:**
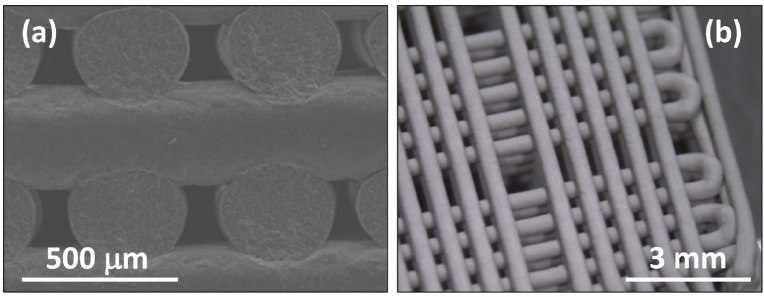
A FastOs®BG bone graft substitute fabricated by robocasting: overall top-view (a); scanning electron microscopy image presenting the transverse and cross-sectional shape and contact of constituting intersecting glass filaments (b). Adapted with permission from Ref. [258].

The sintering ability of FastOs®BG, as accessed by DTA and HSM analyses, revealed that fully dense amorphous materials could be obtained by heat-treatment at relatively low temperatures (800 °C). These features are important for the production of mechanically strong bioactive glass scaffolds for bone regeneration. 3D porous scaffolds with pore sizes of 200 μm, 300 μm, and 500 μm could be easily fabricated from FastOs®BG by additive manufacturing [258] from printable inks containing 47 vol% solids with rheological properties set to meet the strict requirements of robocasting technique (Fig. 10). The fully densified filaments obtained upon sintering conferred to the scaffolds compressive strength values that were higher in comparison to cancellous bone. A similar BioExtrusion technique was used to produce polycaprolactone (PCL)–BG composites scaffolds containing 20 %, 30 %, and 35 % FastOs®BG [267]. The addition of BG was found to decrease the elastic gradient and yield stress if two scaffolds of the same density are compared.

**Fig. 11:**
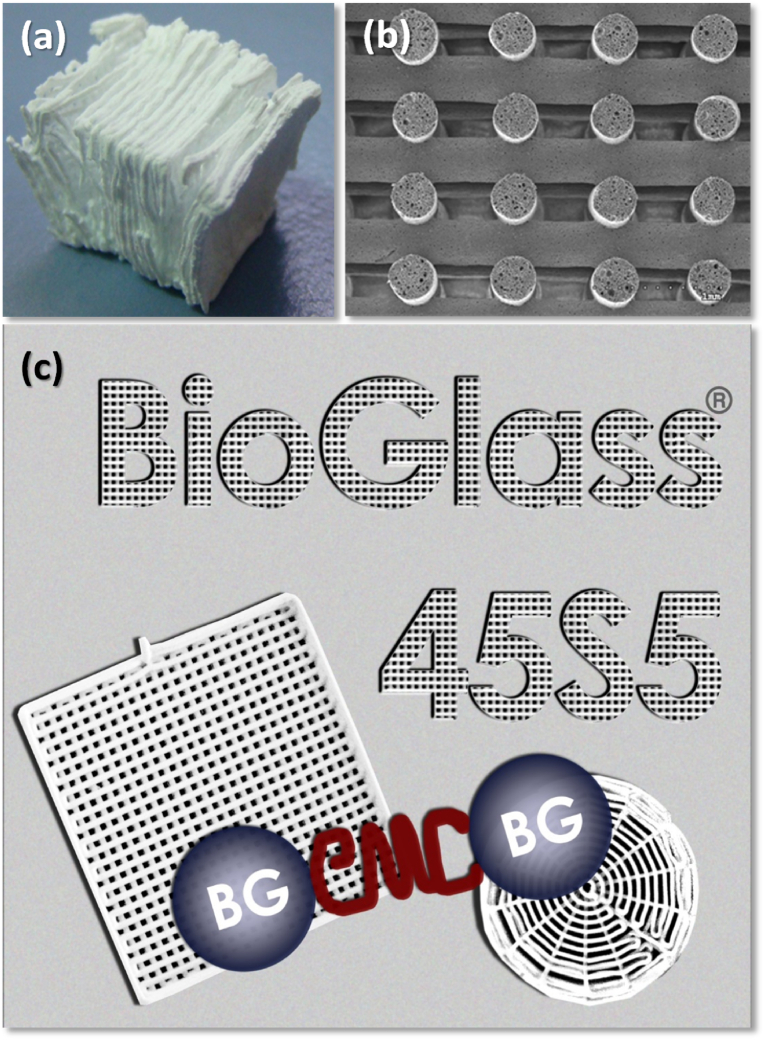
Porous scaffolds fabricated for the first time from 45S5 Bioglass® by robocasting: (a) The cuboidal construct exfoliated upon the first sintering attempts due to the internal stresses between the successive deposited layers and the weak mutual bonding by the limited diffusion occurring within the narrow sintering window (T_c_-T_g_) leading to extensive crystallization, and consequently poor mechanical strength; (b) Scaffold with internal porosity sintered under a slight load to prevent exfoliation; (c) Schematic demonstration of this achievement.

In contrast to FastOs®BG, the first successful deposition of 3D porous scaffolds from 45S5 Bioglass® [268,269] was accomplished by our group in collaboration with other Spanish researchers from University of Extremadura, as illustrated in Fig. 11.

They had been unsuccessfully attempting to overcome the typical processing difficulties of this material, for about one and half years, by following the traditional approach for preparing inks for robocasting. It is worth mentioning that our main motivation was satisfying a special request from Prof. Pedro Miranda. The team from University of Extremadura, Spain, intended using 45S5 Bioglass® as fashionable starting material for the fabrication of porous scaffolds for biomedical applications.

The traditional approach commonly includes adding anionic dispersants and other processing additives to maximize the solid loading, followed by the addition of a cationic coagulant to drastically change the viscoelastic properties of the inks and allow shape retention capability to the extruded filaments. Unfortunately, this approach completely fails in the case of 45S5 Bioglass®. Indeed, the partial dissolution and the release of sodium ions to the aqueous media confer a negative surface charge to the dispersed 45S5 Bioglass® particles, with a weak affinity toward adsorbing anionic dispersant species, while conferring them a thick electrical double layer and an effective apparent size much greater than their hard size, thus limiting the solid loading of the suspensions [268,269]. On the other hand, the aqueous suspensions of 45S5 Bioglass® exhibit high pH values (pH ∼10), at which the cationic agent is in its non-dissociated form, being therefore hindered from acting as an effective coagulant. These processing difficulties and their poor understanding might have discouraged or impeded other researchers to get there. Overcoming these processing obstacles required a new approach using a single multifunctional processing additive carboxymethylcellulose (CMC) that acts as dispersant, binder, and gelation agent, permitting to obtain aqueous suspensions with 45 vol% solids (about 50 % higher than when using several other anionic and cationic dispersing agents in preliminary assessment experiments). Therefore, it is not surprising that even without a firm motivation other than removing the stones from their way, we have achieved the goal for the first time by thinking out of the box.

#### 3.3.3 Compositional refinements in AFBGs

According to the previous results, FastOs®BG was selected as the parent glass composition for another study aiming at investigating the influence of partially replacing CaO by SrO regarding the structure, apatite-forming ability, physico-chemical degradation, and sintering behaviour of a new BG series with the composition (mol %): (36.07−*x*)CaO–*x*SrO–19.24MgO–5.61P_2_O_5_–38.49SiO_2_–0.59CaF_2_ (with *x* = 0–10) [261]. The results revealed that the Sr^2+^/Ca^2+^ ratio did not significantly affect the glass structure, but the apatite-forming ability of glass powders decreased considerably after soaking in SBF for different periods of time varying between 1 h and seven days. Moreover, the addition of Sr led to a seven-fold decrease in chemical degradation of glasses in Tris–HCl and citric acid buffer. Fully dense GCs with a residual glassy phase between 31 and 47 wt% were obtained after sintering glass powder compacts for 1 h at 850 °C, resulting in the crystallization of diopside as the dominant phase, while FA was also formed as the secondary phase. Flexural strength values between 98 and 131 MPa were obtained. The large amounts of residual glassy phase, along with the good flexural strength, proved the potential of the developed GCs for the scaffold fabrication in bone tissue engineering.

Important studies on Sr-containing bioactive glasses were published in 1995 by Galliano et al. [270,271], but the interest in Sr-doped bioactive glasses has increased only after the patents of Sr-containing bioactive glass compositions by Hill and Stevens [272] and Jallot et al. [273], and from the boom in the number of scientific publications [[274], [275], [276], [277]]. These studies demonstrated the benefits of Sr-doping bioactive glass for their *in vitro* performance. Hill and Stevens [272] patented a series of strontium-containing bioactive glass compositions, among which a glass with (wt%) 44.08SiO_2_–24Na_2_O–21.60CaO–4.43SrO–5.88P_2_O_5_ is being commercialized under the Stron-Bone™ trademark by RepRegen Ltd (London, UK).

A similar study was also carried out starting from FastOs®BG as the parent glass composition and partially replacing MgO by ZnO in the composition (mol%): 36.07CaO–(19.24−*x*)–MgO–*x*ZnO–5.61P_2_O_5_–38.49SiO_2_–0.59CaF_2_ (with *x* = 0–10) [252,262]. The aim was to investigate the effects of the Zn^2+^/Mg^2+^ ratio on the structure by molecular dynamics simulations and nuclear magnetic resonance spectroscopy. The network connectivity of these glasses is lower than that reported for 45S5 Bioglass® [278]. An increase in the Zn^2+^/Mg^2+^ ratio did not induce any significant change in the *Q*^*n*^ speciation and network connectivity, but the chemical durability of the glasses was improved, tending to suppress the HCA forming ability in SBF [262]. Other parallel work aimed at investigating the influence of the partial replacement of MgO by ZnO on the structure, sintering ability, crystallization behaviour, and bioactivity [252]. The ZnO content was revealed to play an essential role in the *in vitro* bioactivity. The proliferation of mesenchymal stem cells (MSCs) and their ALP activity on GCs was revealed to be Zn-dose dependent with the highest performance being observed for 4 mol% ZnO, followed by a clear decreasing trend with further increasing ZnO contents.

The influence of SrO and ZnO co-doping on thermomechanical behaviour of alkali-free bioactive glass-ceramics in the system: (mol%) (36.07−*x*)CaO–*x*SrO–(19.24−*y*)MgO–*y*ZnO–5.61P_2_O_5_–38.49SiO_2_–0.59CaF_2_ (*x* = 2–10, *y* = 2–10) was investigated in another work [250]. Thermal analysis (HSM and DTA) revealed that the densification of all the glass powders occurred before the onset of crystallization, resulting in fully densified and mechanically strong glasses/glass-ceramics after heat treating for 1 h at 800 °C, 850 °C, and 900 °C. The crystalline phase assemblage for sintering temperatures >800 °C included diopside and fluorapatite. The results obtained revealed that a better balance of properties was achieved for the samples sintered at 850 °C, providing insightful criteria for selecting the material and experimental conditions for the development of three-dimensional porous scaffolds for bone tissue engineering.

#### 3.3.4 Osteogenic and antioxidant properties of AFBGs

**Fig. 12:**
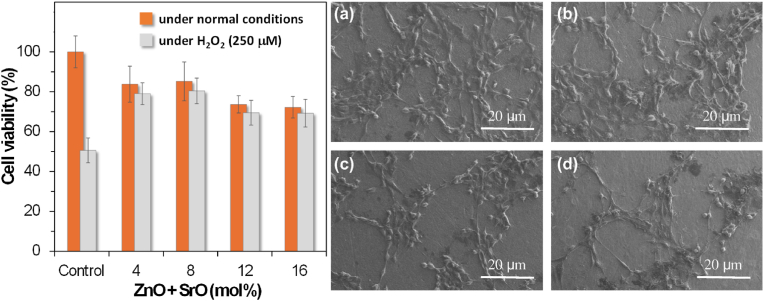
On the left-hand side: cell viability showing the cell growth kinetics of the glasses (ZS-2, ZS-4, ZS-6 and ZS-8): (a) during culture after 4 days under normal conditions and (b) under H_2_O_2_ (250 μM) induced oxidative stress in MG63 cell culture after 3 days. On the right-hand side: SEM morphologies of cells (MG63 cell line) at the semi-confluent stage on the surface of glass samples during culture for 3 days: (a) ZS-2, (b) ZS-4, (c) ZS-6, and (d) ZS-8. Adapted with permission from Ref. [251].

The same above referred compositions were further investigated for the structure–property relationships in order to shed light on the structural role of co-doping ions (Sr^2+^ and Zn^2+^) on the chemical dissolution behaviour of glasses and its impact on their *in vitro* bioactivity [251]. The relevant structural properties were well-correlated to the degradation behaviour, *in vitro* bioactivity, osteoblast proliferation, and alleviation of the oxidative stress levels experimentally induced on the human osteosarcoma MG-63 cell line (using a hydrogen peroxide procedure). A dose-dependent cytoprotective effect of glasses with respect to the concentrations of Zn and Sr released was observed, enhancing the cell viability and negating the effect of oxidative stress induced by the addition of H_2_O_2_ to the cell culture medium, as illustrated in Fig. 12, side by side with the SEM morphologies of cells at the semi-confluent stage on the surface of glass samples after 3 days of culturing. This cytoprotective effect of co-doped glasses contrasts with the cytotoxicity effect exerted by 45S5 Bioglass® [198,199,203,204,251]. Moreover, relatively high cell densities can be observed onto the surface of the ZS2 and ZS4 samples, denoting excellent cells-materials interactions. The affinity of the cells toward the surface of the samples apparently tends to decrease with increasing added amounts of ZnO and SrO. Remarkably, the co-doping of FastOs®BG with Zn and Sr also allowed to tailor the CTE of glasses (particularly for the composition with ZnO and SrO in 6 and 4–6 mol%, respectively), matching it to the CTE of titanium and its super-alloys (the current materials of choice for the fabrication of endo-osseous implants) [256].

The most promising BG compositions (Di-60 to Di-90) were further *in vitro* investigated in human mesenchymal stem cell (hMSCs) cultures, together with other well performing BG compositions reported above, namely, undoped FastOs®BG (TCP 20), and co-doped with equimolar amounts (2–4 mol%, ZS2, and ZS4) of ZnO and SrO, replacing MgO and CaO, respectively. The investigated compositions are summarized in Table 5 [220].

**Table 5:** Alkali-free bioactive glasses compositions (mol%) *vs.* 45S5 Bioglass®.

Type of glass	SiO_2_	P_2_O_5_	CaO	Na_2_O	MgO	ZnO	SrO	CaF_2_
FastOs®BG	38.49	5.61	36.07	–	19.24	–	–	0.59
ZS-2	38.49	5.61	36.07	–	17.24	2.00	2.00	0.59
ZS-4	38.49	5.61	36.07	–	15.24	4.00	4.00	0.59
Di-70	38.48	5.76	36.52	–	19.24	–	–	–
Di-80	42.57	3.71	32.44	–	21.28	–	–	–
45S5 Bioglass®	46.10	2.60	26.90	24.4	–	–	–	–

**Fig. 13:**
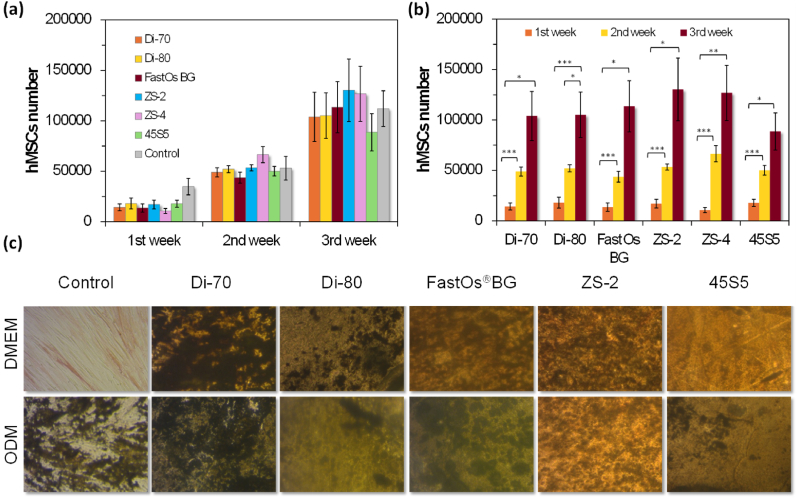
Analysis of hMSCs proliferation when incubated on bioactive glasses studied for 1, 2 and 3 weeks. The results in (a) and (b) represent the mean and standard error of three independent experiments performed in triplicate. (c) Effects of BGs on osteoblast differentiation assessed by ALP activity analysis and von Kossa assay. For all conditions studied hMSCs were cultured in DMEM and osteogenesis differentiation medium. The images are representative of three independent experiments performed in duplicate on three different days. Images were obtained at a magnification of 10 × . Adapted with permission from Ref. [220].

An assessment of biological performances of these selected alkali-free BGs with respect to 45S5 Bioglass® used as control was made [220], aiming at evaluating and comparing their abilities to stimulate hMSCs differentiation into osteoblasts. The results are summarized in Fig. 13. The comparison of data obtained for different incubation times (Fig. 13a) did not reveal any statistically significant differences among the studied BGs concerning their effects on hMSCs proliferation. An increase in the hMSCs proliferation with increasing the incubation time is clearly observed for all conditions studied. In contrast, there were almost always statistically significant differences among the number of cells in the first week of incubation and following weeks identified with * for p < 0.05, ** for p < 0.01 and *** for p < 0.001 (Fig. 13b). The results of von Kossa assay (Fig. 13c) demonstrated that all the studied BGs were able to induce the appearance of calcium deposits, thus, demonstrating their ability to foster the hMSCs differentiation. It was also observed that in both of the cell culture media tested (Dulbecco's modified Eagle's medium and the osteogenesis differentiation medium), the alkali-free BGs clearly induced the appearance of more calcium deposits than 45S5 Bioglass®, indicating their improved performance with respect to cell differentiation. Significant statistical increases in the metabolic activity of hMSCs when compared to the control were observed for Di-60 and Di-70 glasses under both basal and osteogenic conditions [253].

All of the investigated glasses experienced considerably lower weight losses in Tris–HCl in comparison to that of 45S5 Bioglass®, but showed improved *in vitro* mineralization capacity expressed by the formation of an HCA surface layer after 7 days of soaking in SBF. These results clearly demonstrated that alkali-free BGs are safe and superior alternative materials for dental, orthopaedic, and maxillofacial surgery applications in comparison to 45S5 Bioglass® [220].

In another recent work, Kapoor et al. [260] investigated the biological effects (*i.e.*, the *in* *vitro* apatite-forming ability, cytocompatibility, and cytoprotective potential) of Cu^2+^, Co^3+^, Mn^2+^ and Fe^3+^ (transition metal ions known to act as essential micronutrients in bone metabolism) incorporated in a newly developed Zn and Sr co-doped alkali-free bioactive glass. Even if only marginal effects were inflicted at a structural level, the bio-mineralization capacity was slightly affected by Cu^2+^, Co^3+^ and Fe^3+^ doping. This was not the case of MnO incorporation, for which no remarkable structural changes were observed, and furthermore, it delivered good apatite-forming ability. The cytocompatibility tests (in MG-63 human osteosarcoma cell cultures) suggested that, for safety, the transition metal oxide substitutional levels should be kept in the range of 1 mol%. MnO (1 mol%) and Co_2_O_3_ (3 and 5 mol%) substituted alkali-free glasses exhibited significant cytoprotective responses against the H_2_O_2_ mediated oxidative cell damage. This was linked to Mn capability to efficiently quench the peroxyl radicals according to the reaction: R–OO·+Mn(II)→H^+^ + ROOH +Mn(III) [279]. Such an anti-apoptotic trait could benefit bone regeneration and healing applications.

### 3.4 Antibacterial efficacy of bioactive glasses

In the last decade the World Health Organization (WHO) has declared that the antimicrobial resistance (AMR) is one of the top 10 global public health threats [280]. In order to counteract the spread of AMR a new body was created, namely the Global Antimicrobial Surveillance System (GLASS), that, in 2022, published a report based on data collected from 127-member countries, territories and areas with both low and high income [281]. The report revealed rapidly increasing cases of high-level AMR to a number of serious bacterial infections, suggesting an acceleration toward the *post-antibiotic era* prognosed by WHO, a time when common infections and minor injuries could become lethal [282]. Thus, AMR is a world-wide health and development threat, requiring urgent multisectoral action. The GLASS reported that, in some areas of the world, more than half of the infections is caused by one major category of bacteria — Gram-negative, which includes *Escherichia coli* and *Klebsiella pneumoniae* — now resistant to carbapenem-drugs [283].

In order to cause a disease, a bacterium has to survive, multiply and alter the tissue structure or function. As such, any substance or combination of substances that is able to diminish the survival, multiplication or virulence of the bacteria could prove useful for counteracting infection of the surgical site. Nowadays, expectations are rather low for the near future since in the last 30 years only two new classes of antimicrobials were clinically introduced, and only 15 candidate drugs with potential antimicrobial effect are under study, mostly in second tier pharmaceutical enterprises [284,285]. Nevertheless, new hopes recently stemmed from the promising antimicrobial efficacy of various metallic ions [286,287].

Bone presents a poor accessibility to circulating antimicrobials, this being a main reason for probing and developing alternative approaches, the most salient one relying on the use of biomaterials for the local drug delivery of designed concentrations of therapeutic agents at the implantation site [288]. BGs are predicted to be employed as therapeutic ion reservoirs [289], aimed to hinder development of bacteria and create an unhospitable microenvironment for pathogens. The *in vitro* experiments generally showed a two-fold up to an order of magnitude decrease in bacterial proliferation in the presence of conventional BGs [290]. Yet, such low magnitude effects are deemed insufficient and new avenues to bolster the antimicrobial efficiency of BGs need to be searched for.

#### 3.4.1 Single ion-doped antibacterial bioactive glasses

Great historical experience exists with respect to the Ag and Cu effects on bacterial growth, some medical devices being used to efficiently treat deep ulcerated skin lesions caused by poor blood flow that are prone to infection with various pathogens. Thus, a newfound interest emerged regarding the prevention and treatment of infections at the orthopaedic/dental intervention site. First Ag-doped BGs arisen, and their encouraging antibacterial performances, stimulated and expanded researches worldwide, with a plethora of studies now tackling the single doping in BGs [32].

Although not yet fully understood, the antimicrobial mechanism of Ag is suggested to rely on Ag ions reaction with the bacterial cell membrane, disrupting its function, coupling and inactivation of several enzymes and important proteins by interaction with thiol groups, and also bacterial DNA binding [291]. No microbial resistance to Ag ions was documented so far, this fact being attributed to the large number of affected cellular systems [292]. Most studies indicate a silver dose-dependent bacteriostatic or bactericidal effect against numerous bacteria strains such as *Staphylococcus aureus*, *Staphylococcus epidermidis*, *E. coli*, *Streptococcus mutants*, *Enterococcus faecalis*, *Pseudomonas aeruginosa*, and *Listeria monocytogenes* [[293], [294], [295], [296], [297]]. Gholipourmalekabadi et al. [298] studied the effects of CaF_2_/Ag-doped BGs on multi-resistant antibiotic bacteria isolated from patients with burns. They found an efficacy comparable to antibiotics for 1 wt% Ag-doped BG samples, whilst for the CaF_2_-containing BGs a positive, but insufficient response was recorded. However, currently there are serious concerns with respect to Ag potential cytotoxicity, linked mostly to its DNA binding capacity, which subsided in some respect this research direction and determined a shift of focus towards other antimicrobial-potent metallic ion candidates [299,300].

The roadmap to finding alternate highly-antibacterial effective doping agents can rely on the identification of enzymes that need specific metallic ions which can be replaced by targeted therapeutic ions with close enough properties, but with potential inactivating effect. In this respect, BGs incorporating promising cations such as copper, zinc, strontium or gallium were recently advanced and their antimicrobial effect, in parallel with bone tissue dynamic (accounting for the destruction/production balance and mineralization equilibrium), angiogenesis and/or anti-oxidant capacity, was surveyed [31,251,289,301,302].

Cu is the second most-studied antimicrobial agents, being effective against both Gram-negative and Gram-positive bacterial strains. Having a low toxicity for humans and an important role in bone healing, Cu is a suitable choice for BG doping [[303], [304], [305]]. So far, the antimicrobial efficacy of BGs doped with Cu concentrations of 1–10 wt% have been studied and provided satisfactory results against *E. coli*, *S. epidermidis*, and frequent oral pathogens such as *S. mutants* and *Streptococcus sanguis* [[304], [305], [306], [307]]. Generally, a dose-dependent antibacterial effect was revealed, having a lower magnitude than that induced by the Ag ions, and depending on type of bacterial strain: *i.e.*, the minimal bactericidal concentration for *E. coli* was found to be 30-times higher for Cu ions than in the case of Ag ions, whilst for *S. epidermidis* the bactericidal concentration of Cu was 8-times higher than for Ag [[304], [305], [306], [307]].

Another candidate for BG doping is Zn, whose antimicrobial mechanism is linked to oxidative stress, alteration of cell membrane functions, and interaction with intracellular proteins [308,309]. As in the case of Ag or Cu, Zn elicits a dose-dependent antimicrobial action [310,311]. However, since ZnO is a network intermediate oxide in silica-based glasses, the concentration of leached Zn depends on the other oxide constituents of the glass system, making hard a *a priori* projection of the dissolution susceptibility for Zn-substituted glasses [312]. Zn containing BGs present satisfactory or strong antimicrobial activity against many bacterial strains: *P. aeruginosa*, *E. coli*, *S. mutants*, *S. aureus*, *S. epidermidis*, *K. pneumoniae*, *Bacillus subtilis*, and *Actinomyce*s *viscosus* [310,311,[313], [314], [315], [316]].

Amongst, the candidate antimicrobial cations for BG doping is Sr, an alkali-earth metal currently used in the treatment of osteoporosis as Sr ranelate, being able to promote a fast bone formation and mineralization [317]. Hence, a series of Sr-containing inorganic biomaterials have been produced over the last decades in a quest to enhance bone healing and remodelling [276,318,319]. The Sr antibacterial effect is hypothesized to be linked to the interference with the growth and proliferation, cell metabolism, chromosome replication, as well as with the normal function of the bacterial cell wall [[320], [321], [322]]. The magnitude of the antibacterial effect of Sr depends on both its concentration and type of bacterial strain; a lower effect was observed for *S. aureus* than for *E. coli* [322]. Sr-substituted BGs show effectiveness against subgingival oral pathogens, such as *Aggregatibacter actinomycetemcomitans* and *Porphyromonas gingivalis* [321].

The antimicrobial effect of Ga^3+^ ions is related to its similarity to Fe^3+^ ions [301,323,324]. Substitution of Fe^3+^ with Ga^3+^ ions in different enzymes leads to their inactivation, because gallium ions cannot assume a 2^+^ state such as Fe^2+^, a transition which is necessary for many cell's processes [325]. Since most bacteria have important iron containing proteins and enzymes, Ga can induce a marked antibacterial activity by hijacking the bacterial iron metabolism [325,326]. No antibacterial resistance mechanism against Ga has been reported to the best of our knowledge. Ga ions were found to provide a bactericidal effect against many bacterium species – *S. aureus*, Methicillin resistant *S. aureus* (MRSA), *S. epidermidis*, *P. aeruginosa*, or *E. coli*, as well as against oral pathogens such as *P. gingivalis, Streptococcus gordonii*, and *S. mutants* [301,324,[327], [328], [329]]. A concentration of 1 mol% Ga_2_O_3_ was found sufficiently high to endow strong antimicrobial effect to the glass [301].

Despite significant efforts, identifying the optimal composition and therapeutic ion dosages for incorporating various oxides as antimicrobial carriers in silica-based glasses remains intangible. This challenge stems from diverse experimental approaches, including SBG synthesis methods (MQ – melt-quenching or SG – sol-gel), antibacterial tests (dissimilar typology of assay, with variations in BG mass/testing medium ratio or bacterial concentration), and forms (powder, bulk, fibres, coatings). Additionally, factors such as particle size (for MQ and SG glasses) and textural parameters – surface area, average pore diameter, pore volume (for SG glasses) are further complicated matters. Furthermore, the lack of completeness in experimental design, such as the omission of pH and ion-release assessments in relevant testing media concerning antibacterial testing, often diminishes dependability. Table 6 provides an overview of the types, substitutional ranges, leached concentrations in testing media, and effects of antibacterial ions singly incorporated into bioactive silica-based glasses. This table is indented to filter information based on criteria such as the exclusive use of silica-based glasses, relevant to the efforts of CICECO-hub scientists (under scrutiny), adoption of experimental designs incorporating ion release tests, and the prevalent utilization of SBGs in powder form (mostly). Its purpose is to provide a glimpse into the possibilities rather than exhaustively cover the available literature.

**Table 6:** Types, substitutional ranges, leached concentrations in testing media, and effects of antibacterial ions singly incorporated into bioactive silica-based glasses.

Therapeutic ion/Type of oxide	SBG system (mol%)	Synthesis method	Substituted oxide	Doping range (mol%)	Therapeutic ion release	pH	Type of antibacterial test	SBG mass/testing medium volume ratio (mg/mL)	Testing medium	Bacterial strains	Main effect	Refs.
**S-BLOCK ELEMENTS**												
Li/Li_2_O	70SiO_2_–30CaO	Sol-gel	CaO	5; 10; 20	Not determined	The pH after 24 h of immersion remained at ∼6 in artificial saliva, ∼8.5 in HBSS, and between 7.6 and 7.8 in SBF.	● Broth microdilution method	100 mg/mL	Lysogeny broth (LB) containing ∼10^7^ CFU/mL	● *E. coli;* ● *S. aureus.*	Overall, non-doped and lower Li-doped SBGs exhibited the most pronounced antibacterial effects after 24 and 48 h.	[330]
Mg/MgO	58S: 60SiO_2_–36CaO–4P_2_O_5_	Sol-gel	CaO	1; 3; 5; 8; 10	∼35–38 mg/L after 24 h in SBF	The pH of ∼7.5 after 24 h in SBF.	● Suspension growth inhibition	10 mg/mL	LB containing 0.5–2 × 10^8^ CFU/mL	● *MRSA*	At 24 h, there was a dose-dependent bacterial survival rate of ∼75 %, 63 %, 60 %, 55 %, 65 %, and 70 % for MgO contents of 0, 1, 3, 5, 8, and 10 mol%, respectively.	[331]
Sr/SrO	42SiO_2_–39CaO–4P_2_O_5_–15Na_2_O	Melt-quenching	CaO	1.95; 19.50; 39	∼10–320 mg/L after 24 h in α-MEM	Not determined	● alamarBlue™	0.002 g/mL and 0.02 g/mL	Brain heart infusion broth, containing 10^6^–10^7^ CFU/mL	● *A. actinomycetemcomitans;* ● *P. gingivalis.*	Sr showed a dose-dependent effect against *P. gingivalis*, while its impact on *A. actinomycetemcomitans* only slightly increased with concentration, over a period of 2–6 h.	[321]
Sr/SrO	58S: 60SiO_2_–36CaO–4P_2_O_5_	Sol-gel	CaO	5; 10	∼18; ∼28 mg/L after 24 h in SBF	∼7.45 after 24 h in SBF	● Suspension growth inhibition	10 mg/mL	LB containing 0.5–2 × 10^8^ CFU/mL	● *MRSA*	Sr-doped SBG exhibited higher bactericidal activity compared to the undoped counterpart.	[332]
**P-BLOCK ELEMENTS**												
B/B_2_O_3_	S53P4: 53.85SiO_2_–21.77CaO–1.72P_2_O_5_–22.65Na_2_O	Melt-quenching	SiO_2_	13.46; 26.92; 40.38; 53.85	Dose dependent release of ∼10–15 mg/L after 24 h in SBF.	The pH rose from 7.69 to 8.19 and from 7.71 to 8.35 after 7 and 14 days of incubation in SBF, respectively, with progressive B_2_O_3_ substitution.	● Broth microdilution method	100 mg/mL	Mueller-Hinton (MH) broth	● *E. coli* – ATCC® 25922™	B_2_O_3_-substituted glasses induced bactericidal effects, whereas undoped S53P4 only inhibited bacterial growth.	[333]
Ga/Ga_2_O_3_	80SiO_2_–15CaO–5P_2_O_5_	Sol-gel	SiO_2_	1; 2; 3	Dose dependent release of ∼0.08–0.3 mg/L after 12 h in Tris-HCl.	Not given	● Killing time assays	Not mentioned	TSB containing 4 × 10^8^ CFU/mL	● *E. coli;* ● *S. aureus.*	Ga-SBGs demonstrated a superior antibacterial effect against *S. aureus* compared to *E. coli*.	[334]
Ga/Ga_2_O_3_	60SiO_2_–40CaO	Sol-gel	CaO	1; 3; 5	max. 1 mg/L after 24 in PBS	∼7.4–8 after 24 in PBS	● Broth microdilution	10 mg/mL	LB containing 10^7^ CFU/mL	● *E. coli;* ● *S. aureus.*	After 24 h, the 5 mol% Ga-doped SBG showed the highest effectiveness against *S. aureus*, whereas the 1 mol% Ga-doped SBG was more effective against *E. coli*.	[302]
Bi/Bi_2_O_3_	S53P4: 53.85SiO_2_–21.77CaO–1.72P_2_O_5_–22.65Na_2_O	Melt-quenching	Equal wt.% equal parts of SiO_2_ & Na_2_O	0.13; 0.27; 0.54; 1.13	Dose dependent: ∼4–30 μg L^−1^ (after 7 days in SBF) ∼3–12 μg L^−1^ (after 14 days in SBF)	pH decreased dose dependently: 7.98 to 7.74 (7 days in SBF); 8.05 to 7.75 (14 days in SBF)	● Broth microdilution method	100 mg/mL	MH broth	● *E. coli* – ATCC® 25922™	After 24 h, the 0.13 mol% B_i2_O_3_-doped glass exhibited superior cell proliferation rates as well as bactericidal effects.	[335]
**D-BLOCK ELEMENTS**												
Mn/MnO	50SiO_2_– 40CaO– 10P_2_O_5_	Sol-gel	CaO	3; 5; 7	No Mn release was observed in DMEM after 72 h.	Not given	● Spectroscopy measurements	pellets	LB	● *B. subtilis*– 168*;* ● *E. coli* – C600*;* ● *P. aeruginosa* – PAO1*;* ● *S. aureus* – DSM20231.	Both undoped and 5 mol% Mn-doped SBG decreased growth across all bacterial strains.	[336]
Cu/CuO	85SiO_2_– 15CaO	Sol-gel	CaO	2; 5	∼10 and 5 mg/L for the 2 and 5 mol% Cu-doped SBGs, respectively, after 24 h in SBF.	The pH of SBF remained within the physiological range throughout testing.	● Broth microdilution method; ● MTT test.	0.031, 0.063, 0.125, 0.25, 0.5, 1 and, 2 mg/mL	LB containing ∼10^10^ CFU/mL	● *E. coli* – RB*;* ● *S. aureus* – 8325–4*;* ● *S. epidermis* – RP62A.	Cu-doped SBGs exhibited high effectiveness against all tested bacteria after 24 h. The 2 mol% Cu-doped SBG suspension, at a concentration of 2 mg/mL, successfully reduced and disrupted the S. epidermis biofilm matrix.	[337]
Zn/ZnO	70SiO_2_– 26CaO– 4P_2_O_5_	Sol-gel	CaO	3; 5	Not detected at short incubation times (in SBF)	The SBG solubilization induced an increase of the LB medium pH to 8–8.2.	● Broth microdilution method	0.6 mg/mL	LB containing ∼2 × 10^8^ CFU/mL	● *B. subtilis;* ● *P. aeruginosa.*	The 5 mol% SBG exhibited a 91.3 % inhibition against *B. subtilis* and 89.4 % inhibition against *P. aeruginosa* after only 2 h of incubation.	[311]
Zn/ZnO	70SiO_2_– 30CaO	Sol-gel	CaO	2; 4	Dose dependent: ∼0.08 & ∼0.65 mg/L after 24 h in distilled water. Dose dependent: ∼0.03 & ∼0.08 mg/L after 24 h in distilled SBF.	pH increased to 7.8 and to 8.2 for melt-derived glasses and gel-derived glasses, respectively.	● Broth microdilution method	10 mg/mL	LB	● *E. coli;* ● *S. aureus.*	The 2 mol% Zn-doped melt-quenched, 4 mol% Zn-doped sol-gel, and undoped sol-gel SBGs demonstrated a significant antibacterial effect against both types of bacteria.	[338]
		Melt-quenching			Dose dependent: ∼0.3 & ∼0.8 mg/L after 24 h in distilled water. ∼0.08 & ∼0.14 mg/L for Zn2 and Zn4, after 24 h in distilled SBF.							
Zr/ZrO_2_	58S: 60SiO_2_–36CaO–4P_2_O_5_	Sol-gel	CaO	5; 10	Dose dependent: 0.22 & 0.26 mg/L after 24 h in SBF.	∼7.48 after 24 h in SBF	● Suspension growth inhibition	10 mg/mL	LB containing 0.5–2 × 10^8^ CFU/mL	● *MRSA*	The 5 mol% Zr-doped SBG demonstrated the highest antibacterial efficacy (∼70 %) after 24 h.	[339]
Ag metallic	80SiO_2_–15CaO–5P_2_O_5_	Sol-gel	n.a.	1; 5; and 10 (Ag/(Ag + SBG)	5.5–5.8 mg L^−1^ in 10 % TSB	Not given	● Disc diffusion; ● Time-kill curves; ● Colony-forming assay.	1.3, 2.5, 5, 10, 20, and 40 mg/mL	TSB containing 5 × 10^5^ CFU/mL	● *MRSA* – ATCC® 33592™	Minimal inhibitory concentrations (MIC) of 10 mg/mL were observed after 24 h, regardless of Ag doping.	[340]
Ag/Ag_2_O	25.43SiO_2_–32.68CaO–10.89P_2_O_5_–31.00MgO	Melt-quenching	CaO, MgO	1; 2	32.08–40 mg L^−1^ m^−2^ (after 16 h in SBF, 2.6 mg SBG/mL)	5.72–6.17 (in distilled water)	● ASTM E2149 (suspension growth inhibition)	0.025 and 0.1 mg/mL	LB containing 10^9^–10^10^ CFU/mL	● *E. coli* – ATCC® *25922™*	The low-Ag derived glass-ceramic exhibited both non-cytotoxicity and antibacterial activity after 24 h.	[341]
Ag/Ag_2_O	64SiO_2_– 26CaO– 5P_2_O_5_–5Ag_2_O	Sol-gel	n.a.	5	Ag concentration in solution was markedly lower in the presence than in the absence of bacteria (0.2 vs. 0.03 mM at 24 h), suggesting bounding to bacterial cells.		● Suspension growth inhibition	0.05–20 mg/mL	Peptone medium with 0.2 % glucose containing 2 × 10^8^ CFU/mL	● *E. coli* – MG1655	Potent antimicrobial effects (MIC of 0.2–1 mg/mL), after 24 h.	[342]
**F-BLOCK ELEMENTS**												
Ce/Ce_2_O_3_	60SiO_2_–40CaO	Sol-gel	CaO	1; 3; 5	Bellow detection limit	∼7.4–7.8 after 24 in PBS	● Broth microdilution	10 mg/mL	LB containing 10^7^ CFU/mL	● *E. coli;* ● *S. aureus.*	After 24 h, the 3 mol% Ce-doped SBG showed the highest effectiveness against *S. aureus*, whereas the 1 mol% Ce-doped SBG was more effective against *E. coli*.	[302]
Ce/Ce_2_O_3_	13-93: 54SiO_2_–22CaO–2P_2_O_5_–6Na_2_O–8MgO–8K_2_O	Melt-quenching	CaO	1; 3; 5; 8	∼0.07 mg/L in deionized water after 24.	Not given	● Broth microdilution	10 mg/mL	Broth containing 7 × 10^7^ CFU/mL	● *E. coli* – ATCC® 25922™; ● *S. aureus* – ATCC® 25923™.	The 5 mol.% Ce-doped SBG exhibited 91.2 % and 83.54 % antimicrobial activity against *E. coli* and *S. aureus*, respectively.	[343]

When analysing the total content of dopants and their influence, contradictory levels of biofunctionality often arise, highlighting the complexity of the issue and the need for a unified approach. This is unsurprising, given that the release rate of cations is influenced by numerous factors – both internal, such as network connectivity, specimen form (*e.g.*, bulk, particle, or fibres), morphology, and porosity, and external, such as the testing medium (distilled water, simulated body fluid, cell culture medium, nutrient broth). An interconnected understanding necessitates insightful studies that compare the effects of multiple cations within a study framework and focus on actual ionic release rates, rather than solely on the doses of SBG powder or the nominal composition of oxides of interest. It's worth noting that internal factors are significantly influenced by differences in the chosen synthesis methods and technological protocols, while external factors depend on researchers' readiness to move away from simplistic testing protocols (such as conducting ion release experiments in unsuitable media like distilled water and simulated body fluid), instead of adopting a unified approach by conducting joint testing of biofunctional properties (cytocompatibility, antimicrobial effects, ion-release temporal profiles) using consistent assays and testing media.

Researchers are urged to supplement their bio-functional assays with the determination of temporal ion-release profiles, obtained through ICP techniques with ppm/ppb sensitivity. These systematic studies could consolidate multiple and congruent demonstrations of the promise of a given cation and its optimal action dose, enabling trustworthy conclusions and facilitating the reliable and safe transition of therapeutic ions-substituted BGs from research benchwork to commercial and/or clinical applications, thereby offering significant health and societal benefits.

It is worth mentioning that a significant amount of research is still necessary to enable understanding of the mechanisms underlying the antimicrobial efficacy of bioactive glasses. There are often instances where undoped or lightly doped glasses exhibit greater effectiveness against pathogens than heavily doped ones, as evidenced in Table 6. Since 2002, Larry Hench, regarded as the father of BGs, has questioned whether the observed antimicrobial effects are related to compositional features of BG or to the significant changes imposed on the incubation medium, such as pH, ionic strength, or osmotic pressure [294]. It is essential to investigate this, as only through a thorough understanding of the influencing factors can smartly designed SBGs with predictable biofunctional behaviour be achieved. The local increase in pH, often considered the main determinant of antimicrobial activity of SBGs, should not be readily accepted. Such an increase could potentially lead to protein ionization, alteration of their functions, and induction of cell necrosis.

#### 3.4.2 Complementary antibacterial effects of co-substituted AFBGs

The good and satisfactory results obtained by single-oxide substitutions in BGs very recently led to the combinatorial ion-doping concept, aimed to maximize the antimicrobial effect (and extend its range against a wider array of pathogens), while preserving good cytocompatibility along with other highly relevant bio-functional features (*e.g.*, osteogenic and angiogenic capacities). However, in contrast to single-doped BGs, whose properties generally follow an intuitive curve, a more complex structural and dissolution behaviour should be expected for multi-substituted BGs, since the incorporated dopants can elicit different structural roles, function of concentration [[250], [251], [252],^259^,^301^,^344^]. Furthermore, when employing additives that can alter the network connectivity in an intricate manner (*e.g.*, network intermediate oxides), compositional series need be envisioned and explored such as to enable higher chances of disclosing the complex responses of BGs and forge a path towards functional predictability. The combinatorial doping becomes even more attractive since the leached ions can act synergistically (and not necessarily additively) blocking different pathways and processes with a lower chance of adaptation for the bacterial cells, and thus leading to an increased effect and lack of resistance in time [256,259]. This specific field of BG research is still in its infancy, with only few studies published to date.

In 2019, at the CICECO-Aveiro Institute of Materials were synthetized by melt-quenching compositional series of SrO and/or ZnO doped glasses (partially substituting CaO and MgO, respectively) using as parent system the alkali-free FastOs®BG formulation [256]. While cytocompatible in human mesenchymal stem cells cultures, all doped glasses exhibited antibacterial effects against *S. aureus* and *E. coli* (more marked for the former). The best antibacterial effects were achieved for the doubly-substituted glasses (ZnO = 4–6 mol%; Sr = 4–6 mol%), which, after 24 h, led to 2-log and 1-log CFU reductions with respect to seeded bacterial CFUs of *S. aureus* and *E. coli*, respectively. After 48 h, 4-log and 3-log reductions were recorded against *S. aureus* and *E. coli*, respectively. The results pointed towards the synergic action of complementary Zn and Sr ions.

In the period 2020–2022, Ferreira and collaborators, explored the coupled structural and antibacterial effects of CuO and/or Ga_2_O_3_ FastOs®BG-doped melt-quenched glasses (partially substituting CaO and MgO, respectively), in both powder and thin film (fabricated from a selected Cu-Ga-FastOs®BG composition by magnetron sputtering) [248,259]. All Cu and/or Ga-doped BGs (powders and films) were found cytocompatible in NIH/3T3 mouse fibroblast cell cultures. The double-doped CuO (2–3 mol%) and Ga_2_O_3_ (2–3 mol%) melt-quenched glasses inhibited the bacterial development of *S. aureus* with 1–2 orders of magnitude with respect to the single Cu (5 mol%) or Ga (5 mol%) substituted glasses. The best antimicrobial efficacy against *S. aureus* (at 24 h) was obtained for the melt-quenched glass doped with 2 mol% CuO and 3 mol% Ga_2_O_3_, which induced a 2-log reduction with respect to the seeded CFUs. This glass composition was deposited on Ti substrates in form of highly-adherent and wear-resistant ∼600 nm-thick films, which delivered a comparable antibacterial efficiency against *S. aureus* (*i.e.*, a ∼2-log reduction). The excellent mechanical, cytocompatible and antimicrobial functional responses of the Cu-Ga-FastOs®BG-derived films advocated for their potential and supports their implementation in demonstrators to be *in vivo* tested on animal model in a future development stage.

In 2021, Lung et al. [345] incorporated three dopants (*i.e.*, Ag – 2 mol%, Co – 2 mol%, and Ti – 2 mol%) – substituting SiO_2_ – in 58S-based glass (mol%: SiO_2_ – 58, CaO – 37, P_2_O_5_ – 5) sol-gel coatings. The triple-doped system provided good cytocompatibility in MC3T3-E1 osteoblast-like mouse cell cultures and satisfactory antimicrobial properties against *P. gingivalis*.

In 2022, Correia et al. [346] manufactured by sol-gel technology BGs in the alkali-free SiO_2_–CaO–MgO–P_2_O_5_ system triply-substituted with SrO (10–15 mol%), ZnO (6–10 mol%), and CuO (2–3 mol%). Overall, the glasses exhibited antimicrobial efficacy against two frequently encountered microorganisms in endodontic pathology – *E. faecalis* and *Candida albicans*. Namely, the BGs were bacteriostatic against *E. faecalis* at a dose of 15 mg/mL and killed the *C. albicans* cells with 2–3 orders of magnitude at the same concentration of 15 mg/mL, and proved to hinder their proliferation at a concentration of 5 mg/mL. These BGs hold promise for dentistry applications.

Taking in account the already known antimicrobial elements, the possibilities for obtaining new biocompatible and antibacterial multi-doped BGs are tremendous, limited only by funding, manpower, and will to carry-out extensive and insightful testing.

## 4 Overcoming challenges, achieving breakthroughs, and expanding the innovation of materials beyond bone regeneration applications

### 4.1 Tackling challenges through ongoing innovation and adaptation: A series of case studies

Materials innovation frequently encounters obstacles like high costs, restricted scalability, regulatory hurdles, and technological constraints. Surmounting these challenges demands interdisciplinary collaboration, inspiration, substantial investment in research and development, and a readiness to explore unconventional approaches. Below, we briefly review 10 examples from the extensive experience of the CICECO-hub scientists.

#### 4.1.1 Advancing the development and refinement of Direct Consolidation Techniques

The breakthroughs by Prof. Ferreira started even before the formation of CICECO. Aiming at maximizing the green density of silicon carbide, SiC powders with different particles sizes were mixed, enabling achieving relative green densities superior to 76 %, measured in cylindrical rods (d = 8 mm, length = 120 mm) consolidated by slip casting, using the top and bottom portions of each rod. Under certain conditions, the cross section of the rods exhibited a clear peripheric outer layer enriched in fine particles, while the coarser ones preferentially concentrated along the central part. There was no satisfactory explanation in literature for the observed particles’ segregation. This phenomenon is driven by the liquid removal from suspension under certain processing conditions, and when the particle sizes of the powders dispersed are significant different. The effects of all relevant experimental variables and their values windows were then investigated. The results, and their clear interpretation, were disclosed in his PhD thesis in 1993, and later in an article [347]. Although the expression “Clogging Effect” had been already coined, the concept was ill-defined and misleading, namely, when referring to particle segregation occurring in the tape casting process, which can only be attributed to gravity (and other improperly selected and misunderstood processing conditions). The clear interpretation of this particle segregation mechanism illustrated the importance of Direct Consolidation Techniques (DCT) that enable transforming flowable suspensions into rigid green bodies without liquid removal, such as Gel Casting (GC) [348], Direct Coagulation Casting (GCC) [349], and Hydrolysis-assisted solidification (HAS) [350]. In this regard, Starch Consolidation (SC) was another great breakthrough steaming from the team of Prof. Ferreira [351], as mentioned in the *Supplemental Information (SI)* and shown in Fig. SI–1 and Fig. SI–2.

#### 4.1.2 Preparation of Extremely high concentrated suspensions of different ceramic powders

This represents another notable achievement by the Prof. Ferreira's team in addressing a recurrent technical challenge. For alumina, solid volume fractions ≥70 vol% enabled to consolidate green bodies just by pouring the suspensions in impervious moulds, followed by drying [352]. For HA, solid loadings up to 60 vol% were reported for the first time [353]. Moreover, the initial shear-thickening behaviour of the suspensions could be suitably changed to an almost Newtonian or slightly shear-thinning flow behaviour, favouring wet processing, and preventing the clogging of the fine deposition nozzles in extrusion-based 3D-printing techniques. The know-how about dispersion and processing porous ceramics by the polymeric sponge method was plagiarized, as detailed in the SI, and used to accomplish within a few months, a Master Thesis entitled “*Manufacture of Hydroxyapatite Foam for Medical Applications*”. In November 5, 2003 (World Materials Day), the author was awarded the prize from the European Federation of Materials Societies. Based on this never duly acknowledged know-how, in 2008, she created her own company, Medbone® – Medical Devices Ltd, for developing medical devices for orthopaedic and dentistry implant surgeries, for the national and international markets.

#### 4.1.3 Introducing a new generation of durable, yet highly bioactive silica-based glasses

AFBGs for the most demanding applications in healthcare, bone regeneration and tissue engineering are changing the paradigm in the field due to their most salient features: (*i*) Absence of cytotoxic effects (no harmful dissolution products and the resulting pH); (*ii*) Non-genotoxic – no damage to genes within a cell or DNA mutations; (*iii*) Biocompatible – absence of any foreign body reaction; (*iv*) Osteoconductive – bone readily grows on its surface; (*v*) Osteoinductive – recruits immature cells and stimulates them to develop into pre-osteoblasts, essential in any bone healing process; and (*vi*) Osseointegration – stable anchorage of an implant achieved by direct bone-to-implant contact. They have the ability to bond to living tissue, promote bone growth, and protect against microbial infections. Patented at the national level, there was a limited time window (18 months) to extend the national patent of this great breakthrough to other countries/regions to grant its commercial exploitation. But there was no institutional support to cover the internationalization costs. Prof. Ferreira struggled to find out potential entrepreneurs interested in launching a start-up (Reg4Life) to promote the internationalization (United States Patent No.: US 9.238,044 B^2^ [266]) and the translation of the results from bench to market. But the bureaucratic approval of bone implant materials is too cumbersome and requires specific competences that no team member had, and crossing the Death Valley from the safety and efficacy assessing of new devices, the CE Marking in the European Union/FDA-approval was not accomplished within the predicted time. The start-up was later transacted, giving rise to the current “OssMed® Regeneration Technology, S.A.“, belonging to the Institute of Implantology https://www.institutodeimplantologia.pt/. Bone graft products based on Alkali-free bioactive glasses are actually being commercialized by OssMed® (https://www.ossmed.eu/products) under the Ceragraft® trade mark. This commercial designation seems to be an attempt escape to the general poor reputation of “bioactive glasses” among the health professionals, who tend to associate glass-based bone graft implants to the poor performance of 45S5 Bioglass®.

#### 4.1.4 Novel bioactive glass microfibers and scaffolds for real-time monitoring of bone regeneration

Based on the pioneering research on AFBGs by CICECO-hub scientists and on the expertise on fibres drawing existing at the Łukasiewicz Research Network – 10.13039/501100001481Institute of Microelectronics and Photonics, 02–668 Warsaw, Poland, an exploratory bilateral collaboration project was approved by the funding institutions of both countries aiming at the fabrication of AFBGs fibres for biomedical applications. When BG particles are used to fill bone defects, the resulting packing densities are relatively high, leaving little intergranular room to be occupied by the newly formed bone tissue. In such cases, the small specific surface area exposed by the particles tends to prolong the resorption times for a given BG composition.

The primary concept was to develop a kind of cotton-like BG fibres, offering flexibility to fill and conform to the shape of bone defects requiring repair. In this way, the doctors will be able to adjust the fibres according to the desired density, creating a versatile scaffold material with a more open structure to expedite the repair of small bone defects.

**Fig. 14:**
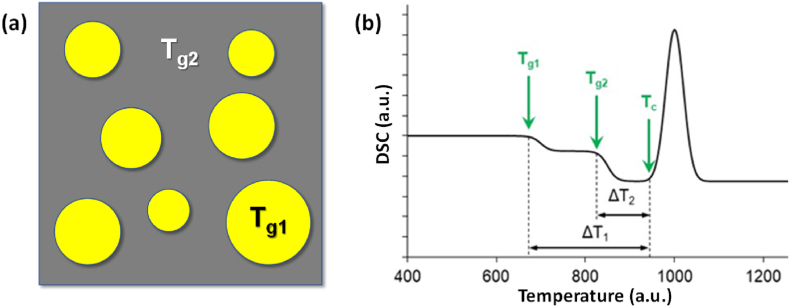
Schematics of: (a) A LLPS glass structure with glass droplets of Phase 1 formed inside the matrix of Phase 2; (b) The sequence of thermal events taking place during the LLPS process.

For this purpose, the FastOs®BG was used as parent glass to design a new series of AFBGs modified by partially replacing silicon dioxide network-former with boron trioxide network-former, utilizing calcium oxide as a charge compensator. This new family of BG compositions preserves the overall exciting properties above referred for AFBGs, while also being suitable to be drawn into fibres, albeit with a few hundred microns in diameter [354]. The parent AFBGs belongs to the CaO–MgO–P_2_O_5_–SiO_2_ system [219], being inherently prone to exhibit stable/metastable liquid-liquid phase separation (LLPS) [355]. This feature facilitates the sintering of the glass powders (see Fig. 8, Fig. 10), as the first glass transition temperature (T_g_) is significantly lower than the crystallization onset temperature (T_c_), providing a wide temperature window for controlling liquid state sintering, as schematized in Fig. 14. Thus, these glasses are well suited for developing biomedical devices that depend on powder processing techniques [356].

Despite the attractive features of boron-containing AFBGs (increased bioactivity, biocompatibility, and antibacterial efficacy) [357], relevant information regarding the chemical nature of the borate species and its structural integration in the BG network was only recently disclosed by H. Eckert, a renowned expert in solid-state nuclear magnetic resonance (NMR) spectroscopy [358].

Considering that the spatial distribution of the phosphate and the fluoride ions in BGs was not enough understood, particularly in systems dominated by LLPS, a fruitful collaboration with the team of Prof. Eckert, was undertaken to assess the structural features of our BGs used for fibers drawing. They were addressed by using a series of advanced solid-state NMR spectroscopy techniques to establish the local environment of boron and its connectivity (B–O–B, B–O–P, and B–O–Si). The spatial distributions of small amounts of P, Na, B, and F in a macroscopically phase separated glass were also addressed [359]. Moreover, the spatial distributions of phosphate, fluoride were determined using static ^31^P and ^19^F Spin Echo Decay (SED) NMR experiments, and interpreted in the context of Monte-Carlo simulations of spatial distribution scenarios. A comprehensive model for the structural organization of this phase-separated glass system was proposed, which can be summarized as follows.

• Heteronuclear dipolar coupling between ^11^B and both, the ^31^P and the ^29^Si nuclei, confirming that B and P occur within the same LLPS phase(s) consisting of Si-richer and Si-poorer regions;
• Minority components F, P, and Na, all occur within a common phase;
• Phosphate species form clusters of sizes 1–4 nm, randomly embedded in an environment more dilute in P;
• The _19_F SED → fluoride ions do not form clusters and are close to randomly distributed, dipolar recoupling with ^31^P;
• ^19^F{^31^P} REDOR experiments, suggest a local environment resembling that of fluorapatite.

Besides the relevant structural elucidations, the last feature helps to explain why fluorapatite [Ca_5_(PO_4_)_3_F–FA] is one of the first crystalline phases formed upon heat treating for 1 h under non-isothermal conditions at 800, 850 and 900 °C of the SrO and ZnO co-doped AFBGs [250]. It also sheds further light on the already reported fast biomineralization process of AFBGs in SBF [219,251]. It is hypothesized that the local fluorapatite environments become soon exposed upon immersion of AFBGs in SBF. Their close resemblance to the amorphous CaP layer formed in the sequence of biomineralization reactions proposed by Larry Hench [215], (Stage 4), would facilitate the subsequent epitaxial-growth of HA crystals due to the close matching between FA (a = b = 9.455 Å, c = 6.888 Å) [360], and HA (a = b = 9.432 Å, c = 6.881 Å) [361] lattices. This would correspond to a leap from Stage 1 to Stage 4 in the 5-stage interfacial reaction kinetics scheme proposed by Larry Hench [215], thus explaining the appearance of a crystalline HA layer after just 1 h of immersion in SBF.

**Fig. 15:**
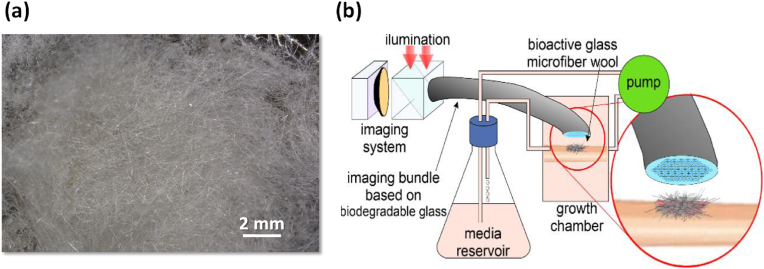
(a) Representative image of cotton-like BG fibres; (b) Schematics of the system for the *in vitro* monitoring of the bone regeneration process.

On the other hand, LLPS negatively affects the fabrication of devices involving glass shaping from the melt, such as fiber drawing and 3D printing. This is because the second glass transition temperature is close to that of the onset of crystallization, thus leaving only a narrow temperature window for glass shaping [354] (see Fig. 14 above). Accordingly, the drawn fibres' diameters varied within the range from about 50 to 220 μm. They may be considered as promising materials for reinforcing constituent of biopolymer matrix composites, but the compositions are not yet satisfactory for producing cotton-like BGs. Hence, to delve deeper into the aforementioned primary concept of creating cotton-like BG fibres for bone repair, a POLONEZ. BIS project was submitted and subsequently approved based on competition, with Prof. Ferreira as Principal Investigator, and Łukasiewicz Research Network – Institute of Microelectronics and Photonics, Poland, as Host Institution. The team effectively combined the experience of Prof. Ferreira with the expertise of the Host Institution in fibres’ drawing and imaging glass fibre bundles. Sharing the experience with the closest team members and other researchers within the Host Institution, training young researchers, and setting collaborative networks are other inspiring concerns to face the societal challenges, while using new creative and science-based approaches. Engaging with industry is another important target, which has been undertaken within the frame of the intersectoral secondment at the Sygnis SA, Poland, that intends expanding the catalogue of 3D printing machines to molten BGs. The project aims not only to produce cotton-like BG fibres (Fig. 15a), but also to engineer an *in vitro* monitoring system of the bone regeneration process, as schematized in Fig. 15b.

Moreover, identifying potential institutional and industrial collaborators willing to establish partnerships, particularly focused in the main research areas of the project, with special emphasis on synthetic bone repair materials and tissue engineering applications, is a key objective, aiming to facilitate the translation of the knowledge from the laboratory to the market.

#### 4.1.5 Development of an innovative surface treatment to protect water-sensitive powders from hydrolysis

This enabled the processing of advanced ceramic materials such as aluminium nitride (AlN) in aqueous media, and its pressure less sintering at moderate temperatures (1750 °C). The sintered bodies exhibited thermal conductivities as high as those so far reported for AlN ceramics processed in non-aqueous media (∼200 W/(m·K)) [[362], [363], [364], [365]].

#### 4.1.6 Facile aqueous processing of barium titanate (BT) powders

BT is one of the most technologically important ferroelectric materials. As mentioned in section 3.2, it has been naively incorporated in biomaterials aiming at exploring its piezoelectric properties to electrically stimulating bone-forming cells. However, extracting practical benefits is challenging and uncertain because, in the physiological fluid, it undergoes hydrolysis and releases poisoning Ba^2+^ ions [206]. Further, the Ba^2+^ ions are exchanged by hydrogen ions, generating very high and cytotoxic pH values [207]. The water sensitivity of BT turns problematic its processing in aqueous media. Renowned experts in this specific field used to systematically attribute such difficulties to the presence of barium carbonate (BC), unreacted during the BT synthesis, or formed under the contact with atmospheric CO_2_. This assertion was not convincing at all, as Prof. Ferreira's team could easily prepare high concentrated aqueous suspensions of BC (up to 60 vol%) with long term colloidal stability. The opportunity to prove that BC was not responsible for the poor aqueous processing of BT arose when a collaborative project in the area of functional materials was approved. It was clearly shown that, contrarily to what has been wrongly and uncritically propagated in specialist literature, the addition of (2 & 5 wt% BC) facilitated the dispersion of BT + BC powder mixtures. The effects of the dispersion conditions on the: (*i*) dissolution extent; (*ii*) formation of Ba–dispersant complexes; and (*iii*) zeta potential and rheology of suspensions, enable to provide persuading insights about the factors controlling the aqueous processing of BT powders in aqueous media [206]. Moreover, it was also shown that the same surface treatment given to AlN powders was effective in preventing hydrolysis of BT and other water sensitive powders such as barium strontium titanate (BST) [[366], [367], [368]], complex lead-free functional ceramics [369,370], and aluminium magnesium spinel [371].

#### 4.1.7 Further understanding and control the surface chemistry in water sensitive powders

**Fig. 16:**
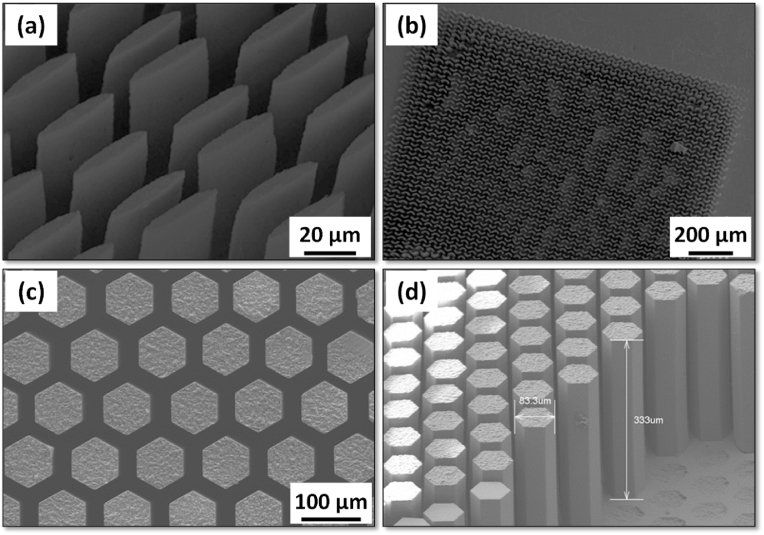
SEM micrographs of green pillar arrays fabricated by EGC: (a,b) PZT (adapted with permission from Ref. [372]) and (c,d) BST (adapted with permission from Ref. [367]).

These advances in the understanding and controlling the surface chemistry of water sensitive powders, and the need to fabricate micro transducers from aqueous suspensions, came together with another breakthrough: the development of Epoxy Gel Casting (EGC), a new and singular DCT. Fig. 16 shows examples of pillar arrays fabricated by EGC from PZT and BST ceramics.

#### 4.1.8 Robocasting printing of macro-porous piezoceramic bone scaffolds

**Fig. 17:**
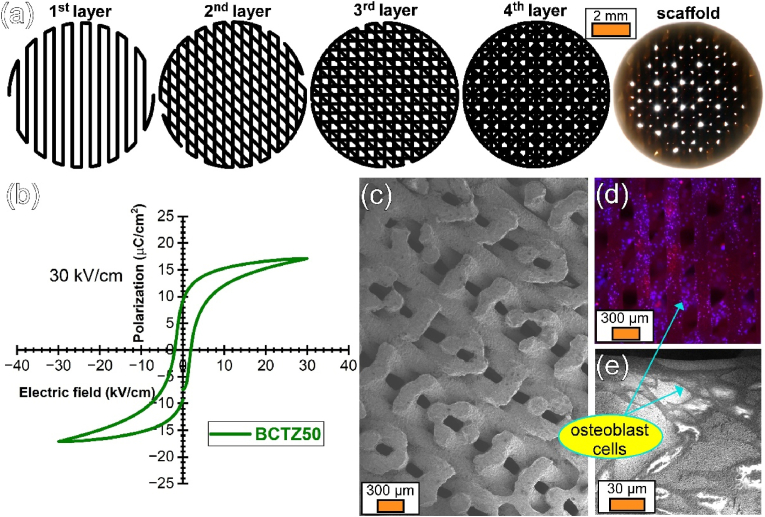
(a) Schematic representation of the printing patterns utilized for the fabrication of the BCTZ50 scaffolds, employing a 45-degrees rotation design. Adapted with permission from Ref. [76]. (b) Graph illustrating the total remnant polarization vs. electric field of BCTZ50. (c) Overview of the filament arrangement and size, as observed by a low magnification FE-SEM analysis of a BCTZ50 scaffold fractured along its height. (d) Successful colonization (after 14 days) of BCTZ50 scaffolds with hFOB 1.19 cells, demonstrated by epifluorescence microscopy focused on the first three printed layers of the BCTZ50 scaffolds. The actin cytoskeleton is stained red (Alexa Fluor™ 546 phalloidin), whilst the cell nuclei are counterstained blue (DAPI). (e) Morphology of hFOB 1.19 cultured for 14 days on the surface of the filaments of the BCTZ50 scaffolds, revealed through FE-SEM. Adapted with permission from Ref. [374].

The skills gained in synthesizing and aqueous processing of environmentally friendly lead-free functional ceramics enabled for successful robocasting printing of macro-porous ceramic bodies based on solid solution within the (Ba_0·85_Ca_0.15_) (Ti_0·9_Zr_0.1_)O_3_ (BCTZ50) binary system [373]. Moreover, within the framework of a comprehensive multi-parametric evaluation encompassing morphological, structural, electrical, mechanical, and *in vitro* biological aspects, BCTZ50 exhibited a range of promising features. These include a dielectric constant 1.5 times higher than the next best material (undoped BT), piezoelectric properties, ability to dissipate plastic deformation energy during mechanical loading, and *in vitro* performance assessed in terms of both cell proliferation and cytotoxicity. These characteristics where highlighted when compared with a selection of lead-free piezoceramics such as KNbO_3_, LiNbO_3_, LiTaO_3_, BaTiO_3_, and Zr-doped BaTiO_3_ [374]. Thereby, BCTZ50 was chosen for the fabrication of bone graft scaffolds by robocasting employing a distinctive 45°-rotated sequential printing pattern (Fig. 17a). The objective was to clearly identify a candidate biocompatible piezoelectric material capable of stimulating bone-forming cells and facilitating the reconstruction of bone defects, paving the way for the next generation of synthetic bone graft substitutes, incorporating both chemical and piezoelectric stimuli Fig. 17b). The BCTZ scaffolds sintered at 1500 °C, with a macro-porosity of ∼50 %, and pore sizes of ∼160 μm, had compressive strength values of ∼20.2 MPa, superior to trabecular bone. Such a macro-porous scaffold design with complex tortuosity (Fig. 17c), mimicking the convoluted arrangement of the bone trabeculae, facilitated uniform cell colonization throughout the entire volume of the ceramic body (Fig. 17d and e) [76,374]. Their cytocompatible response, evaluated by cell proliferation, death, and morphology analyses, was remarkably similar to that of the biological control [374]. This breakthrough marks the successful materialization of the concept explored with BT ceramics, and introduces new research directions.

#### 4.1.9 Deposition of free-standing metallic and ceramic pillar arrays by robocasting

**Fig. 18:**
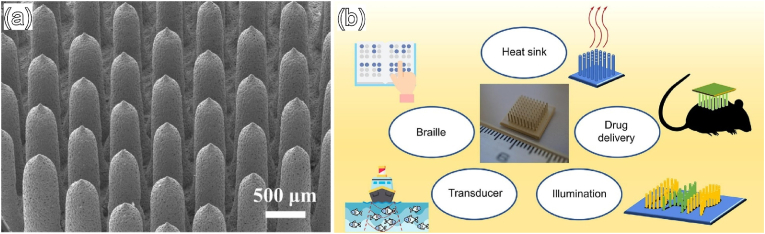
(a) Robocasting printed BCTZ50 pillar arrays as visualized under SEM. (b) Potential applications for the free-standing pillar arrays 3D printed in the vertical direction. Adapted with permission from Ref. [375].

Furthermore, the deposition of free-standing pillar arrays of different materials by 3D printing in the vertical direction was another remarkable breakthrough accomplished (Fig. 18a), for the first time, by the Prof. Ferreira's team [375]. This required a precise control to start, cease and restart the flow. The pillar arrays made of various materials are applicable in many advanced areas, as suggested in Fig. 18b. Versatile degrees of freedom can inspire tissues and organs printing.

#### 4.1.10 Advancing bioactive coatings for endosseous implants through alternative approaches

Advancing bioactive coatings for endosseous implants has long been a focal point aimed at offering alternatives to cemented medical devices made of bioinert materials such as stainless steel, Co–Cr alloys, Ti alloys, alumina, and zirconia [[376], [377], [378]]. The goal is to prolong their lifespans, which are currently restricted to 15–25 years [379,380], rendering them unsuitable for patients under 50 years old. Such surface-active and/or ion-releasing coatings should strive to improve biocompatibility, promote osseointegration, enhance corrosion resistance of the underlying metallic implant, and offer robust alternative antimicrobial defences compared to conventional, increasingly ineffective treatments.

Currently, the predominant approach remains the use of commercially available endosseous implants, which have been in use for close to 30 years and are coated with thick layers (>50 μm) of hydroxyapatite using plasma spraying [[381], [382], [383]]. These types of implants are sanctioned by the U.S. Food and Drug Administration (FDA) [384]. Despite demonstrating suitable biological performance, their substantial thickness makes them susceptible to improper adherence to the substrate [381]. Additionally, their structural and compositional inhomogeneities may lead to uncontrolled and excessively rapid resorption of the coating, increasing the likelihood of micromovement occurrence, particulate-induced osteolysis around the implants, and aseptic loosening [381,385]. Achieving precise control over the structural (percentage and volume distribution of the amorphous component), compositional (content and distribution of secondary/impurity phases throughout the coating), morphological (presence of microstructural defects such as micro-cracks and porosity), and mechanical features may not be feasible with plasma spray techniques.

The biomaterials community promptly began exploring alternative deposition technologies to achieve better control over the coating thickness, morphology, composition, structure, mechanical performance, and biological response. In this regard, radio-frequency magnetron sputtering (RF-MS), a prominent example of physical vapor deposition technologies, emerged as a promising manufacturing method due to several notable advantages: (*i*) virtually no limitations in sputtering any inorganic materials; (*ii*) excellent control over film thickness ranging from a few nanometres to a few thousand nanometres; (*iii*) high purity of films; (*iv*) films with density close to bulk; (*v*) strong film-substrate adherence; (*vi*) exceptional uniformity; (*vii*) low roughness, down to nanometres or even sub-nanometres; (*vii*) low-temperature of operation, if required; (*ix*) easy manipulation of film properties through numerous available process variables such as power density, working gas type and pressure, working gas composition, target-to-substrate separation distance, substrate biasing, and substrate temperature; (*x*) process reproducibility and automation; and (*xi*) scalability and industrial readiness [378,[386], [387], [388]].

**Fig. 19:**
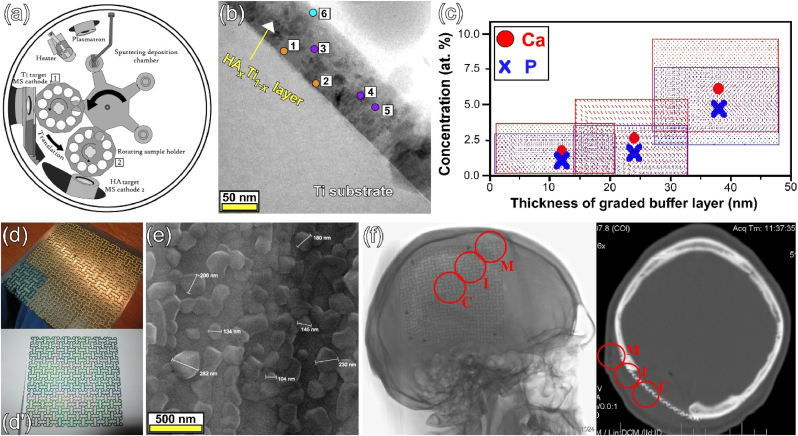
(a) A schematic representation of the experimental design used to produce the compositionally graded HA_x_Ti_1–x_ (x = 0–1) buffer layer by magnetron co-sputtering – reproduced and adapted with permission from Ref. [398]. (b) Morphology of the Ti_1–x_HA_x_ buffer layer evidenced by cross-sectional transmission electron microscopy. (c) Achievement of the Ti → HA compositional gradient as demonstrated by elemental compositional analyses using energy dispersive X-ray spectroscopy, showing a progressive increase in Ca and P values in the layer with distance from the substrate. The concentration values of Ca and P should not be considered absolute due to the strong influence of the Ti substrate on quantification. (d,d') Partially functionalized (d) and fully CHA functionalized (d') cranial Ti mesh. (e) SEM image of the surface of CHA/CHA_x_Ti_1–x_/Ti (x = 0–1) coating assembly deposited onto a cranial Ti mesh. (f) Areas for post-operative imagistic monitoring of osteoconduction and osteointegration – Reproduced from Ref. [395].

In alignment with these global efforts, our cross-national consortium initially delved into manufacturing and enhancing the design of hydroxyapatite RF-MS coatings applied to Ti-based substrates. Ensuring the integrity of the substrate/coating interface is paramount as it directly influences the performance and reliability of any implant-type coating. Notably, as HA coatings sputtered at room-temperature are initially amorphous upon deposition, their durability and medical suitability rely on subsequent crystallization [389], necessitating post-deposition heat treatments [390]. However, such treatments, often performed at temperatures exceeding 500 °C, pose a risk of coating cracking due to the considerable coefficient of thermal expansion (CTE) mismatch between the metallic substrate and the ceramic coating. To address this concern, a novel coating approach was proposed to smooth the abrupt HA/Ti interface. This involved introducing a ∼70 nm HA_x_Ti_1–x_ (x = 0–1) buffer layer with a compositional gradient, achieved by co-sputtering from separate HA and Ti targets while slowly moving the rotating substrate holder from the Ti target towards the HA target (Fig. 19a) [391]. This innovative design effectively addressed the Ti – HA interface discontinuities by incorporating the HA_x_Ti_1–x_ functionally graded buffer layer (Fig. 19b and c). This enabled the complete and excellent crystallization of the top biofunctional ∼600 nm thick HA coating at 550 °C, while providing an outstanding adherence (pull-off) strength of the HA/HA_x_Ti_1–x_ coating assembly, exceeding 85 MPa [391].

In a further endeavour, this conceptual coating design was expanded to intentionally incorporate B-type carbonated (∼4.4 wt % doping) hydroxyapatite (CHA) RF-MS coatings [392]. B-type carbonation, comprising approximately 3–8 wt. % depending on the individual's age, is the preferred substitution in human bone and is known to exhibit enhanced bioactivity and osteoconductivity [393,394]. Comprehensive *in vitro* testing demonstrated that not only this CHA sputtered coating concept supported the viability and proliferation of human osteoprogenitor cells, but provided an optimal substrate for the adhesion of bone cells and the expression of key markers such as alkaline phosphatase, collagen I, bone sialoprotein, osteonectin, and osteocalcin [392]. Furthermore, experiments on cell morphology and tissue mineralization indicated that the CHA coatings promoted the osteogenesis process [392].

The solution involving highly adherent B-type carbonated hydroxyapatite sputtered implant coatings has been patented and successfully utilized for bio-functionalizing titanium meshes for cranial reconstructions (Fig. 19d and d') [395]. These coatings consisted of dense and compact CHA layers composed of polyhedral grains ranging in size from 100 to 300 nm (Fig. 19e). Patients were monitored postoperatively at 3 and 6 months. Osteointegration was assessed through tomodensitometry analyses conducted on the margin (M), intermediary (I), and center (C) regions of the CHA-coated Ti meshes (Fig. 19f). No changes were observed on the Hounsfield unit (HU) scale at 3 months postoperatively in any patients, regardless of whether they received Ti or CHA-coated Ti mesh implants. Cancellous and cortical bone typically yield HU values in the ranges of 300–400 and > 500, respectively [396,397]. At the 6-month mark, density changes suggestive of the initiation of the osteoconduction phenomenon were observed in all patients with Ti meshes biofunctionalized with CHA. Tissue density changes were confined to the mesh's edge, where it contacted the bone, in cases of cranial reconstructions with simple Ti implants. In contrast, in cases utilizing biofunctionalized Ti meshes with CHA, changes on the Hounsfield tissue density scale were noted in both the M (*ca.* 550–850 HUs) and I (*ca.* 310–570 HUs) regions of the implant (Fig. 19f) [395]. This suggests an intensified and progressive osseointegration process induced by the bioactive CHA layer, underscoring its exceptional osseointegration capacity compared to the non-functionalized Ti meshes commonly used in cranial implantology.

Silica-based bioactive glasses (SBGs) have the highest bioactivity index among inorganic materials. However, despite their remarkable biological performance, progress on reliable SBG coatings has been rather marginal due to bonding strength issues and susceptibility to biomechanical stress failure. This has made them slow to transition into real-world implant applications. Recent advancements in SBG compositions, which allow for lowering their coefficients of thermal expansion (CTEs) while maintaining biological properties, have reignited global interest in their use for implant coatings.

CICECO-hub scientists have made significant contributions to these advancements, as mentioned in this overview work. They have not only focused on developing new SBG formulations by reducing or eliminating alkalis [256,399,400] but have also worked on implementing these innovative compositions into durable and biologically responsive RF-MS coatings for real dental implants [196]. Despite the remarkable advantages of RF-MS technology mentioned earlier, there have been drawbacks that needed to be addressed to achieve mechanically and biologically dependable, reproducible, and inexpensive implant coatings. These challenges include (*i*) replicating the composition and structure of the source materials; (*ii*) increasing deposition rates; (*iii*) ensuring cost-effective manufacturing; and (*iv*) achieving adequate step coverage.

**Fig. 20:**
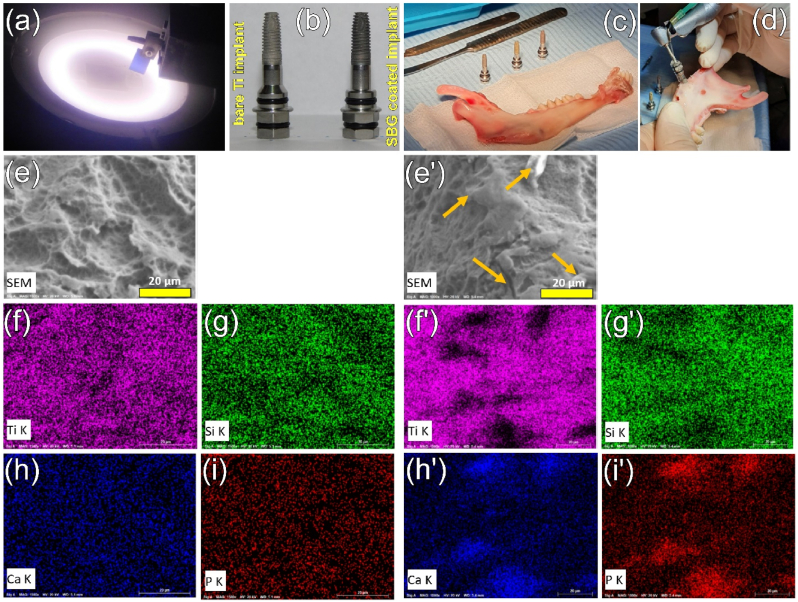
(a) Snapshot captured during the RF-MS coating process of a dental screw with Ga&Cu SBG. (b) Comparative images showing bare and Ga&Cu SBG-functionalized Alpha-Bio® DFI dental implants. (c,d) Representative images obtained during the implantation-extraction procedure of the RF-MS biofunctionalized dental screws. Elemental EDXS mappings performed on SEM micrographs presented in (e,e') collected on the surface of the Ga&Cu SBG type coating deposited on the Alpha-Bio® DFI dental implants, (e–i) before and (e'–i') after implantation. Results subsidiary to research published within Ref. [248].

(i) Early criticisms of RF-MS revolved around its inability to accurately replicating complex material compositions like SBGs, due to the preferential sputtering of the lighter species from the target surface. However, our systematic studies have shown that this obstacle can be overcome. Higher sputtering pressure is preferred for accurately tailoring the replication of source material composition and structure [255,378,401,402]. Additionally, a prolonged target pre-sputtering step (approximately 45–60 min) under the same conditions used for coating deposition is crucial for improving the quality of atomic transfer from the target to the substrate by balancing the ratios of light and heavy elements on the target surface, prior to the actual deposition process starts [255,402,403].
(ii + iii) The low deposition rate of RF-MS, typically around ∼2–3 nm/min, has been doubled by using the economic mildly “cold” pressed targets and employing a lower driving radio-frequency of 1.78 MHz [246,401]. The adoption of powder targets, promoted by the CICECO-hub scientists, offers distinct advantages over compact targets. Powder targets allow for higher target powers without risking damage, and they ensure reproducible coatings by providing a fresh target surface for each deposition session, eliminating the need to modify the surface composition of compact targets.
(iii) Since SBG compositions are constantly evolving, with new biofunctional traits added by incorporating therapeutic ions (*e.g.*, B, Zn, Sr, Cu, Ga [251,256,259,399]), one cannot speak of a universal RF-MS processing regime. Each target composition requires specific process adjustments, but the key influencing factors have been identified [378]. Moreover, the abundance of RF-MS variables allows for precise customization of film properties using a single target composition. This enables adaptation of both mechanical and biological performances, rendering the process economically attractive.
(iv) Coverage of step and 3D featured substrates, even with complex geometries like endosseous implants, is no longer a challenge for RF-MS coating processes. As demonstrated, in Ref. [196], the simple solution of translating/rotating the substrate efficiently overcomes most shortcomings (Fig. 20a and b).

To complement the encouraging outcomes from conventional static mechanical tests [195,248], the CICECO-hub scientists opted to further conduct a “cold implantation” of SBG-coated dental screws in a pig/sheep mandibular bone to obtain a more precise evaluation of their performance (Fig. 20c and d). This method exposed the screws to the intricate mechanical stresses experienced during implantation by screwing. Notably, the bonding strength between the SBG and the Ti-based dental screws proved resilient, with no observed delamination or cracks following implantation and tension-free extraction. This is exemplified here for dental screws coated with Ga&Cu-containing SBG using the refined recipe from Ref. [248] *via* scanning electron microscopy (SEM) and energy dispersive X-ray spectroscopy (EDXS) coupled analyses collected before (Fig. 20e–i) and after (Fig. 20e'–i') implantation.

In addition to engineering the apatite-forming ability [244,246,249,255], RF-MS SBG layers have demonstrated excellent cytocompatibility across a wide range of cell cultures. These include mouse fibroblast NIH/3T3 [195,248], human fibroblast Hs27 [404], human umbilical vein endothelial HUVEC [402], human osteosarcoma MG63 [405] and Saos-2 [404,406], human primary osteoblast hOB [194], human dental pulp stem [196], and human mesenchymal stem [255] cell lines. This demonstrates their readiness for further *in vivo* testing on animal models.

All of these aspects and more were eloquently reviewed in a recent comprehensive study, encompassing the three-decade-long global efforts in the field of sputtered bioactive glass coatings [378].

### 4.2 Expanding the innovation of materials beyond bone regeneration applications

Recent years have witnessed the extended applications of bioactive materials especially in the field of medical imaging, thus resulting in the development of variety of techniques and modalities for diagnosing and monitoring diseases. Magnetic resonance imaging (MRI), computed tomography (CT), and optical imaging have become invaluable tools in clinical practice, providing non-invasive imaging of tissues and organs with high spatial resolution and excellent soft tissue contrast. In order to enhance the visibility and contrast of orthopaedic implants, the use of contrast agents along with bioactive materials is crucial, which demands the periodic observation to track their performance over time. In this context, the authors have explored the doping of lanthanides with bioactive materials owing to its similarities with Ca^2+^ as contrast agents for monitoring the regeneration mechanism without the need of invasive surgical procedures to inspect the condition of the implant [104,105,[107], [108], [109],[407], [408], [409], [410], [411], [412], [413]].

Gadolinium (Gd^3+^) doped *β*-Ca_3_(PO_4_)_2_ samples were synthesized by precipitation method with four different mol% of Gd^3+^ [104]. The maximum number of unpaired electrons resulted in the paramagnetic behaviour of all samples with the upsurge in magnetic moment from 0.133 to 0.429 emu/g with respect to the increase in Gd^3+^concentration. The relaxivity values obtained were relatively higher than the relaxivities values of commercially available contrast agents. A similar result has been obtained for Gd^3+^ doped zirconia toughened alumina (ZTA) samples [409]. Dysprosium (Dy^3+^) on the other hand, exhibits both optical, MRI and CT imaging capabilities owing to its 4*f-f* transitions, large K-edge value of 53.8 keV and high X-ray mass absorption coefficient [107]. Thus, the potential of Dy^3+^ as a contrast agent has been explored by doping Dy^3+^ in *β*-Ca_3_(PO_4_)_2_. Overall, the system exhibits luminescence behaviour under the excitation at 350 nm with the emission centres at 480 nm (blue) and 572 nm (yellow) and negative contrast ability with transverse relaxivity value r_2_ of Dy^3+^ doped *β*-Ca_3_(PO_4_)_2_ was 3.43 mM^−1^s^−1^, which is comparable to the values of other Dy compounds reported in the literature, and exhibited maximum CT contrast value of 145 HU.

Ytterbium (Yb^3+^) substitution in the *β*-Ca_3_(PO_4_)_2_ and ZTA offered near infrared luminescence and CT contrast imaging that is aided from its minimal shielding of 4*f* electrons, high *K*-edge value (61 keV) and large atomic number (*M* = 70) [109]. Yb^3+^ doped samples exhibited NIR emission at 983, 997 and 1024 nm under the excitation of 980 nm and produced nearly 450 HU, which is comparable to the commercially available Omnipaque. Subsequently, co-substitutions of Gd^3+^ and Dy^3+^ in *β*-Ca_3_(PO_4_)_2_ have been explored and the energy transfer process from Gd^3+^ to Dy^3+^ was evidenced by photoluminescence studies [108]. The paramagnetic nature of the samples has been identified from the hysteresis loop and the longitudinal and transverse relaxation rates (1/*T*_1_ and 1/*T*_2_), as a function of the Dy^3+/^Gd^3+^ concentration displayed a correlated linear relationship.

In a similar way, Gd^3+^ and Dy^3+^ substituted ZTA yielded results comparable to ones induced by simultaneous substitution of Gd^3+^ and Dy^3+^ in *β*-Ca_3_(PO_4_)_2_ [410]. Co-substitution of Gd^3+^/Dy^3+^/Yb^3^ in *β*-Ca_3_(PO_4_)_2_ revealed up conversion luminescence in the visible region under the excitation at NIR region due to the energy transfer from Yb^3+^ to Dy^3+^ [105]. The obtained CT contrast ability and transverse and longitudinal relaxivity values were comparable to that of commercially available contrast agents. The luminescence characteristics of terbium (Tb^3+^) substitutions in ZTA has been detailed in another investigation [411]. Under the excitation at 285 nm, two green emission bands were observed in 480–510 nm and 530–560 nm region. Europium (Eu^3+^) substitutions in ZTA exhibited emission at 613 nm and 593 nm under 393 nm excitation [412]. Here, the upsurge in the concentration of Eu^3+^ has led to a decline in the emission intensity due to the concentration quenching effect. The collective substitutions of rare-earth (Yb^3+^, Dy^3+^, Tb^3+^, Gd^3+^, Eu^3+^, Nd^3+^) in Y_2_O_3_, ZrO_2_, and Y_2_O_3_-Ln_2_O_3_ explored in another investigation [407,408,413] revealed up conversion and down conversion emissions in the visible and NIR regions under excitation at 793 and 350 nm, respectively.

## 5 Impact on clinical practice

The progresses driven by CICECO-hub scientists on the synthesis and processing of advanced CaPs created opportunities for translating the results from the bench to the market. This has been accomplished for CaP powders by the spin-off “Agoramat - Produção de Materiais Cerâmicos, Lda” that also undertook the first validation steps for bone grafts, which were later continued by the “CERAMED – ALTAKITIN” group to get the regulatory clearances for Class III Medical Devices. The same target was accomplished by Medbone® – Medical Devices Ltd, as detailed in SI. Both companies are commercializing medical devices for the national and international markets, for clinical applications in orthopaedic, dentistry, maxillofacial and veterinary implant surgeries. But as far as we know, no clinical studies using these materials have been conducted for the two likely reasons: (*i*) they are seen by the authorities as being equivalent to the alike ones pre-existing in the market; (*ii*) the high incurred costs are discouraging.

A similar situation happened for the AFBGs for the most demanding applications in healthcare, bone regeneration and tissue engineering. The CE Marking in the European Union/FDA-approval was accomplished by “OssMed® Regeneration Technology, S.A.“, belonging to the Institute of Implantology group. Bone graft products based on AFBGs are being implanted in dentistry by the group, and commercialized under the Ceragraft® trade mark (https://www.ossmed.eu/products), for the same applications referred above for CaP-based products, as explained in section 4, which addressed the continuous breakthroughs.

Examples of Case Studies involving experts from the areas of biological *in vitro* characterization of biomaterials and of *in vivo* testing in different animal models are given in section 2.7, specifically for injectable bone cements. Besides the easiness of handling the cement pastes based on ion-doped CaPs (Fig. 5), their superior *in vitro* and *in vivo* performances in comparison to its commercial counterpart, Norian SRS®, were clearly demonstrated when implanted in a pig animal model (Fig. 6) [167].

Six 6-month-old male Wistar rats were used by our collaborators from the Faculty of Dentary Medicine of University of Porto, Portugal, in the frame of a Master thesis [414] to compare the *in vivo* performances of our two Zn- and ZnSr-substituted bone cements with that of the control, another commercial counterpart, chronOS™ Inject. To eliminate biological variability, two bone defects were made in each rat to receive one of our cements, and the commercial control. The histological analysis revealed a better organized collagen matrix around the cements from University of Aveiro, than in the chronOS™ Inject case, with the collagen fibres oriented circumferentially, consistent with the shape of the Haversian bone, in comparison to the control. Larger amounts of new bone tissue with a greater and more advanced matrix organization, with the presence of Haversian bone, were also found. The Zn- or ZnSr-doped cements from the University of Aveiro performed similarly, with both being slightly superior to chronOS™ Inject.

Further, combining the ion-doping strategy with the addition of retardant sugars to the reacting liquid, dramatically enhanced the interactions between hardened cements and MG63 cells, as shown in Fig. 7 [168].

**Fig. 21:**
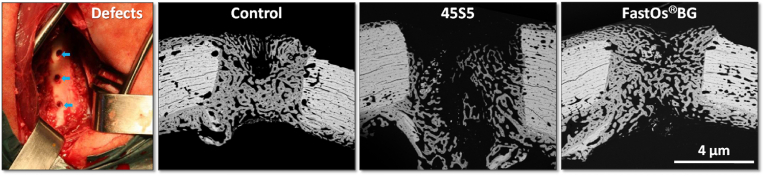
SEM images of femoral bone with drilled defects, and of the histological samples: 1st defect: empty (control); 2nd defect: 45S5; (Reference), 3rd defect: FastOs®BG. Results subsidiary to research published within Ref. [257].

The overall properties of AFBGs have been already well summarized [223], and their enhanced *in vitro* performances reported [220,253] and highlighted in section 3.3.4. So, a move forward was made aimed at assessing the *in vivo* bone grafting performance of FastOs®-BG in comparison to that of 45S5 Bioglass® using an ovine model [257]. The evaluation was made through implantation of both materials for one month, in defects drilled in. Histological and scanning electron microscopy assessments of retrieved bone samples evidence a relatively fast resorption of 45S5 Bioglass®, slowing the regeneration of the defect filled with it, in comparison not only with FastOs®-BG, but also with the empty defect, as shown in Fig. 21.

**Fig. 22:**
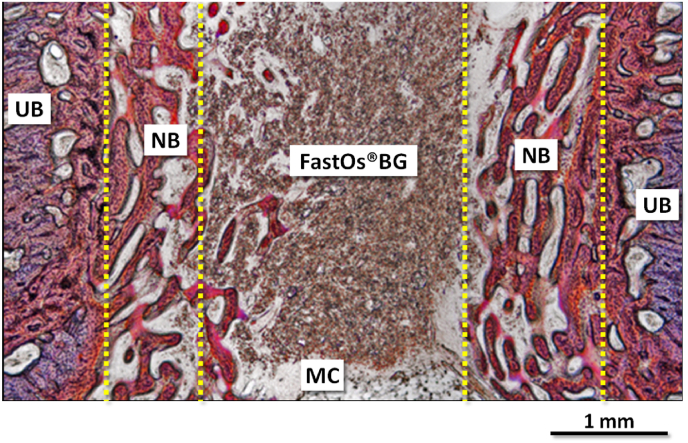
Histological SEM image of a defect filled with FastOs®BG after 1 month of implantation (Solochrome cyanine R). NB = new bone; UB = un-operated cortical bone; BG=FastOs®BG particles; and MC = medullary cavity. Adapted with permission from Ref. [257].

The histological and SEM analyses of FastOs®BG implant samples revealed newly formed bone with a trabecular pattern in a random orientation, resembling woven bone as detailed in Fig. 22.

Healthy new bone embedded with osteocytes of normal morphology, and stemming from the walls of the defects is apparent. No signs of foreign body response, like the presence of a fibrous capsule, could be identified in direct contact with the implanted glasses. The superior features observed for FastOs®BG confirmed its great potential for bone grafting applications to regenerate large bone defects. However, further long-term studies with critical size defects and clinical trials are required to draw firmer conclusions.

**Fig. 23:**
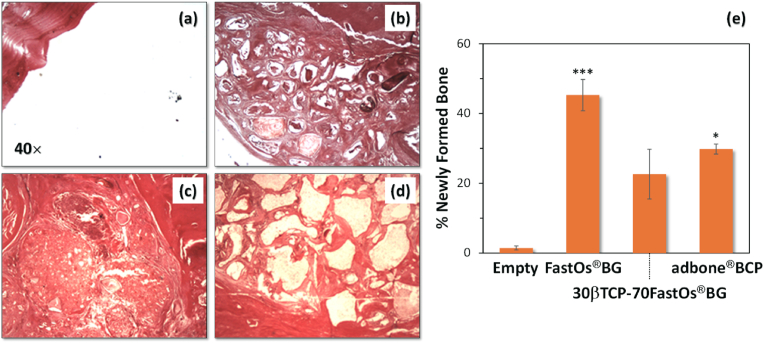
Histological images (H&E staining) of bone defects 9 weeks after treatment and after excision: (a) empty; filled with: (b) FastOs®BG, (c) 30β-TCP–70FastOs®BG, and (d) adbone®BCP; (e) the mean and standard error of at least 5 independent experiments. Significant differences relative to empty group are identified with the use of * that represents p < 0.05 and *** that represents p < 0.001. Adapted with permission from Ref. [415].

Aiming at clarifying if the addition of resorbable β-tricalcium phosphate (β-TCP) to FastOs®BG could bring any further *in vivo* performance benefits, a study was carried out by comparing a new synthetic bone graft (30β-TCP–70FastOs®BG), with those of FastOs®BG alone and of adbone®BCP, a biphasic calcium phosphate, consisting of 75 % HA and 25 % β-TCP (Medbone® – Medical Devices, Portugal) [415]. In this study 10 thirteen-week old Wistar rats were used. Two defects with 4 mm diameter were performed in the calvaria and filled with the bone graft materials. The animals were sacrificed after 9 weeks of implantation and the calvaria was excised. Empty bone defects were used as negative control. The percentages of new bone formed (von Kossa staining) were always higher in the treated groups (FastOs®BG, 30β-TCP-70FastOs®BG and adbone®BCP) than in empty group. Representative histological images of at least 5 independent experiments, together with the percentage of newly formed bone on bone defects 9 weeks after treatment and after excision are shown in Fig. 23.

There were differences with statistical significance between empty and FastOs®BG groups and between empty and adbone®BCP groups. But the differences observed between empty and 30β-TCP–70FastOs®BG groups were less remarkable. The results demonstrated the superior bone regeneration ability of FastOs®BG alone, which was not further enhanced by adding β-TCP in the composition, confirming its already proven regenerative potential.

**Fig. 24:**
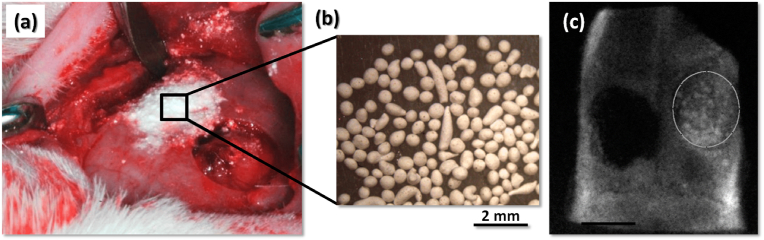
(a) Surgical image of the defects created. One defect was empty (negative control group); the other was filled with composite granules shown in (b), (experimental group); (c) evaluation of the radiographic bone density 9 weeks after treatment and after excision of bone defects. Adapted with permission from Ref. [416].

Considering the clearly perceived benefits of ion-doping in the case of bone cements, a new study was designed aiming at evaluating the effects of co-doping β-TCP with a fixed amount of Mn (0.5 mol%) and different additions of Sr + Zn to obtain the following powder groups: non-doped TCP0; TCP1 (5Sr, 1Zn, 0.5Mn mol%); TCP2 (10Sr, 2Zn, 0.5Mn mol%) [416]. The non-doped and co-doped β-TCP powders were mixed with FastOs®BG in the same 3/7 ratio used in the previous study [415] and used to produce porous synthetic granular composite bone filling grafts (Fig. 24).

The porous granules were implanted in ∼4 mm diameter bone defects drilled in the calvaria of Wistar rats. The animals were sacrificed after 9 weeks of implantation and the calvaria was excised. Non-manipulated bone was used as positive control, while empty defects were used as a negative control group.

**Fig. 25:**
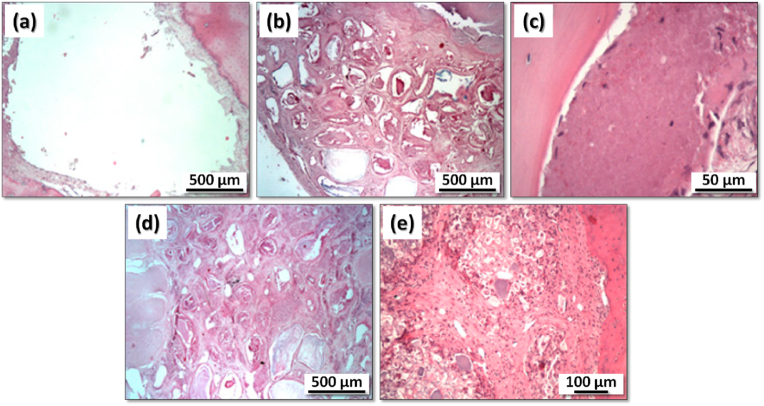
Histological images (H&E staining) of bone defects 9 weeks after treatment: (a) empty defect; and defects filled with: (b) FastOs®BG; (c) 3TCP0/7BG; (d) 3TCP1/7BG; (e) 3TCP2/7BG. The Figure shows representative images of at least 5 independent experiments. Adapted with permission from Ref. [416].

The histological representative images (Hematoxylin and Eosin, H&E staining) of the study groups 9 weeks after the treatment and excision are shown in Fig. 25. The images of empty bone defects reveal a slight formation of regenerated cancellous bone in the edge of the defect (*a*), while in the FastOs®BG group (*b*) small islands of osteoid and bone tissue within a capsule are clearly noticed.

**Fig. 26:**
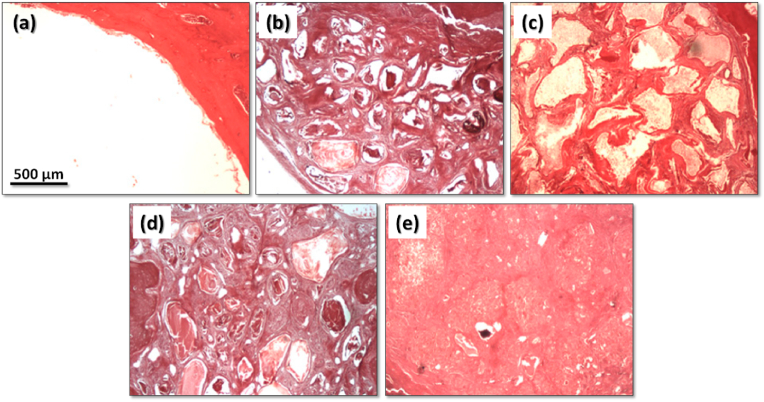
Histological images (VK staining) of bone defects 9 weeks after treatment and after excision: (a) empty defect; and defects filled with: (b) FastOs®BG; (c) 3TCP0/7BG; (d) 3TCP1/7BG; (e) 3TCP2/7BG. The Figure shows representative images of at least 5 independent experiments. Adapted with permission from Ref. [416].

**Fig. 27:**
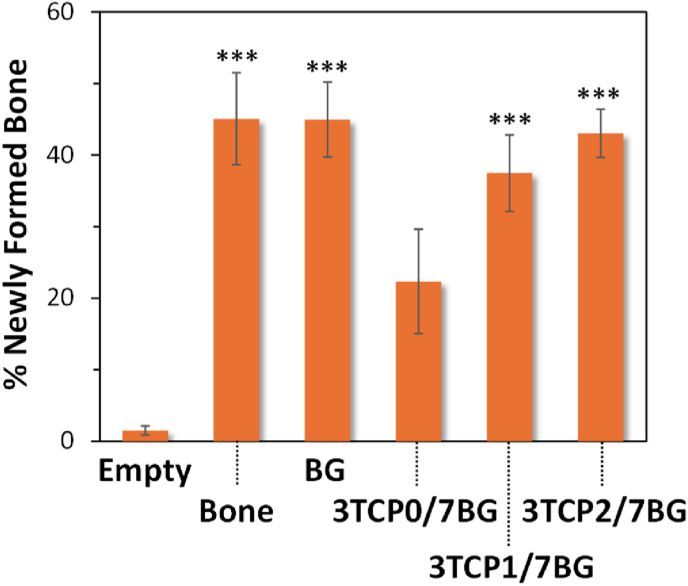
Percentages of newly formed bone in bone defects 9 weeks after treatment and after excision. The Figure represents the mean ± standard deviation of at least 5 independent experiments. Significant differences relative to empty group are identified with the use of *** that represents p < 0.001. Adapted with permission from Ref. [416].

The von Kossa (VK) staining (Fig. 26) revealed an enhanced new bone formation with increasing doping levels, supporting the therapeutic effects exerted by the doping elements. On the other hand, the percentage of newly formed bone (Fig. 27) was similar when the defects were filled with autologous bone, FastOs®BG, or 3TCP2/7BG, which indicates that the latter two are excellent candidates for replacement of autologous bone as bone regeneration material. This finding confirms that doping with suitable doses of therapeutic ions is a good strategy towards transposing the bone graft materials to biomedical applications in humans.

These results are very encouraging towards further testing the most performing materials in future clinical trials. On the basis of the results presented in this study, it appears that doping the β-TCP component in the synthetic bone graft composites may be effective in inducing bone regeneration. This finding confirms that doping the bone graft materials with suitable doses of therapeutic ions is a good strategy towards transposing them to biomedical applications in humans.

## 6 Concluding remarks

This manuscript provides an overview of the most relevant contributes to the state of the art in the field of osteointegrative inorganic materials based on bioceramics, bioactive glasses and composites, especially along the last two decades. The significant breakthroughs stemming from the research team led by J.M.F. Ferreira at CICECO-Aveiro Institute of Materials, together with prestigious networks of national and international expert collaborations in complementary areas are intentionally emphasized. They deserve to be systematized for the first time to the benefit and awareness of the willing readership. The materials developed proved to be suitable for applications in bone repair and tissue engineering in the framework of versatile multi-dimensional approaches, in the form of nanoparticles, micron-sized powders, thin films and coatings for endo-osseous implants, tailor-made bone graft substitutes (porous scaffolds) and dense bulk components.

From the accumulated body of knowledge and information gathered and reported in this review article (and references therein), it is clear that the structure and the desired final properties and functionalities of synthetic bone grafts based on calcium phosphates can be greatly improved, sometimes by small, but smart intended compositional variations. Indeed, the deliberated incorporation in the inorganic components of specific single or combined therapeutic ions that can be released *in vivo* after implantation, brings extraordinary benefits relative to undoped compositions, enhancing cell adhesion, osteointegration and vascularization, or conferring bactericidal effects to prevent infections.

The advances made in bioactive glasses revealed the superiority of well-balanced alkali-free compositions. Their most salient features include absence of cytotoxic effects, biocompatibility, fast biomineralization rate *in vitro* with a rapid formation of an HCA layer, osteogenic capacity, lack of genotoxic effects, good feasibility for scaffold fabrication by traditional and additive manufacturing techniques, ability to release therapeutic and anti-infection ions, and good matching of the coefficients of thermal expansion with metallic substrates for implant coating applications. Achieving synthetic biomaterials comprising all these relevant and essential requirements is the result of a complex and step by step task guided by innovative and smart research approaches.

The progresses made in the synthesis and processing of inorganic bone graft materials are intended for applications in advanced therapies, including the treatment/reinforcing/repair of bone defects caused by osteoporosis, osteonecrosis, osteoarthritis, osteosarcoma, and traumatic accidents. The high-performance synthetic bone graft materials hold great promise for replacing autologous bone grafts, allografts and xenografts, while avoiding all their associated drawbacks. The overall aim was to develop multifunctionality, while offering remedy solutions to face the ever-growing frequency of healthcare challenges derived from the worldwide increase of life expectancy.

Undoubtedly, this is not a final chapter in the journey of a scientist and mentor for many generations, but rather an opportunity to systemize progress and gain clarity for the future. Despite the achievements and merits attained thus far in the synthesis and processing of inorganic bone graft materials, the commitment of our transnational team of CICECO-hub scientists remains steadfast. As we spread across the globe, contributing to the development of a new generation of bioceramics researchers, our dedication to advancing knowledge at the forefront of science remains unwavering. We are committed to continuing our pursuit of discovery with the same passion and dedication, striving to uncover new insights and push the boundaries of understanding. It is through this collaborative effort, free from pride and egotism, that real opportunities for improving healthcare worldwide become tangible.

## Ethics approval and consent to participate

The manuscript entitled “*Two decades of continuous progresses and breakthroughs in the field of bioactive ceramics and glasses from CICECO-hub scientists*”, co-authored by H.R. Fernandes, S. Kannan, M. Alam, G.E. Stan, A.C. Popa, R. Buczyński, P. Gołębiewski, and J.M.F. Ferreira, submitted for evaluation to Bioactive Materials, is a literature review work, and thus no *in vivo* evaluations on animal model or clinical trials were performed in this scope.

Thereby, our work does not fall into the incidence of ethical approvals and patient consents.

## CRediT authorship contribution statement

**H.R. Fernandes:** Writing – original draft, Data curation. **S. Kannan:** Writing – review & editing, Writing – original draft, Data curation. **M. Alam:** Writing – original draft. **G.E. Stan:** Writing – review & editing, Writing – original draft, Data curation. **A.C. Popa:** Writing – original draft. **R. Buczyński:** Writing – review & editing. **P. Gołębiewski:** Writing – review & editing. **J.M.F. Ferreira:** Writing – review & editing, Supervision, Funding acquisition, Conceptualization.

## Declaration of competing interest

The authors declare that they have no known competing/conflicting financial interests or personal relationships that could have appeared to influence the work reported in this paper.
