# Supplementary material for: Two decades of continuous progresses and breakthroughs in the field of bioactive ceramics and glasses driven by CICECO-hub scientists
Source: Bioact Mater. 2024 Jun 8;40:104–47. doi: 10.1016/j.bioactmat.2024.05.041 (PMC11630650; doi:10.1016/j.bioactmat.2024.05.041)

**Supplementary Information to:**

**Two decades of continuous progresses and breakthroughs in the field of bioactive ceramics and glasses driven by CICECO-hub scientists**

H.R. Fernandes^a^, S. Kannan^b^, M. Alam^b^, G.E. Stan^c^, A.C. Popa^c^, R. Buczyński^d^, P. Gołębiewski^d^, J.M.F. Ferreira^a,d*^

*^a^ Department of Materials and Ceramic Engineering, CICECO-Aveiro Institute of Materials, University of Aveiro, Santiago University Campus, 3810-193 Aveiro, Portugal*

*^b^ Centre for Nanoscience and Technology, Pondicherry University, 605014 Puducherry, India*

*^c^ National Institute of Materials Physics (NIMP), 077125 Magurele, Romania*

*^d^* *Łukasiewicz Research Network – Institute of Microelectronics and Photonics, 02–668 Warsaw, Poland*

*Corresponding author: [jmf@ua.pt](mailto:jmf@ua.pt); jose.ferreira@imif.lukasiewicz.gov.pl

***Brief description of CICECO***

The associate laboratory CICECO – Aveiro Institute of Materials, formerly CICECO – Centre for Research in Ceramics and Composite Materials, was created in March 2002 at the University of Aveiro, Portugal, with the mission of developing the scientific and technological knowledge necessary for the innovative production and transformation of ceramics and organic-inorganic hybrids and materials for sustainable development. Initially, it included researchers from Chemistry, Physics, and Ceramics and Glass Engineering (DECV), later (2012) renamed to “Department of Materials and Ceramics Engineering (DEMaC)”. The Dean, having no one with enough status to implement the organization tasks, took back the founder of DECV from IST in Lisbon, where he “took refuge” in protest against unfair academic policies at UA. The “persuading arguments” were not publicly disclosed. After the “nest” has been made, the Dean used the cuckoo strategy and laid his “egg” there...

CICECO is currently the largest Portuguese institute in the field of materials science and engineering, with a staff of almost 500 persons from the initial departments, along with the School of Design, Management and Production Technologies Northern Aveiro from the University of Aveiro. In the last national research assessment (2017/18) performed by the Portuguese Foundation for Science and Technology, CICECO became the national best-rated Materials Science and Engineering Institute. After two decades of productive effort CICECO has become one of the premier research institutions in Portugal and in Europe. More detailed information can be found in the link: <https://www.ciceco.ua.pt/?menu=481&language=pt&tabela=geral>

***Preliminary achievements***

At the first annual meeting (Jornadas do CICECO – 2003), the team led by Prof. Ferreira disclosed examples of potential implants, reproducing the shape of specific bones, or mimicking their dual structure as shown in Fig. SI–1. The Dean staring wide-eyed looking at the Poster, inquired about what would be necessary to materialize such promising ideas? Unfortunately, he swiftly turned his back to Prof. Ferreira when hearing about the need of institutional support to research activities and help creating conditions for an eventual spin-off.

***Fig. SI–1.*** *Hydroxyapatite-based ceramics mimicking the porous trabecular and dense cortical bone structures (a) fabricated by: (1 – slip casting + polymeric sponge method; 2,4 – polymeric sponge method; 3 – slip casting + foaming); (b) dense maxillofacial prostheses for facial reconstruction fabricated by the starch consolidation, dried and sintered at 1300 °C).*

The starting HA powders used for producing the potential examples of implants shown in Fig. SI-1 were from Plasma Biotal Ltd. ([www.plasma-biotal.com](http://www.plasma-biotal.com)), as the team only started the synthesis of its own CaP powders in late 2003. During a visit of Prof. Ferreira to this company, the person in charge of the R&D activities showed him a great variety of attempted developments, including a series of jaw bones, unsuccessfully fabricated by slip casting. The motivation behind was to satisfy a special request from maxillofacial surgeons working at a hospital in London.

The responsible for the R&D activities was then gently informed about the non-feasibility of slip casting to accomplish such target, and a deal was proposed to overcome the situation: in exchange of 3 kg cost-free HA powder, Plasma Biotal would receive a few jaw bone implants fabricated by a new direct consolidation technique (Starch Consolidation – SC) patented by Prof. Ferreira.

Starch granules were initially intended just as environmentally friendly pore formers to fabricate porous bioceramics. But soon, the team realized that starch could act as consolidator agent upon heating starch-containing ceramic suspensions within the temperature range of about 50 – 90 °C. The opening up of polymeric chains of amylose and amylopectin and the concomitant absorption of interstitial water, led to the formation of strong green bodies that could be machined, cut, perforated, *etc*, as shown in Fig. SI-2.


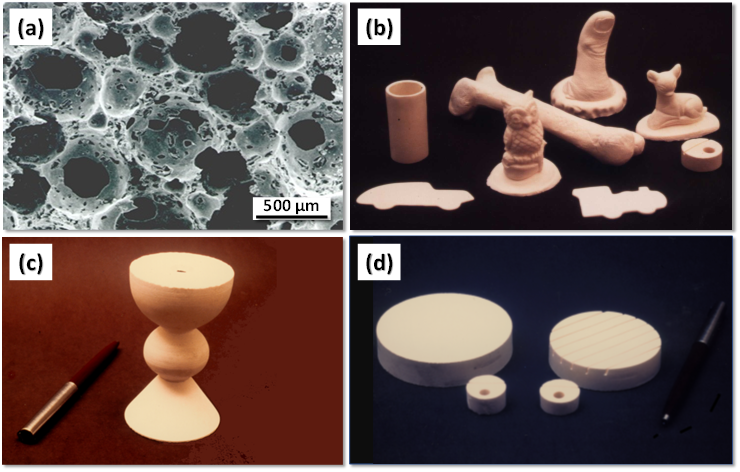


***Fig. SI–2****. Starch consolidation – suitable to produce from almost full dense to very porous materials with complex shapes: (a) Porous scaffold; (b) Examples of complex shaped alumina ceramics obtained by SC; (c) A green massive alumina cylinder after being further lathe shaped in a machine shop, as a demonstration of the high green strength; (d) Disk shaped samples with machined grooves and holes in the green state. The larger sintered disk was intended as support for heat treating glass lamella coated by sol-gel. Adapted with permission from [350].*

Starch Consolidation was a great breakthrough! But the Rectory had not yet implemented any strategy concerning patenting. Therefore, Prof. Ferreira had to cover the incurred costs of patent registration by his own pocket.

***Lack of institutional support and plagiarism***

The preparation for the first time of extremely high-concentrated HA suspensions (up to 60 vol% solids) and their suitable rheological control were other important achievements [352]. The results were firstly disclosed in a Poster presentation at Materiais 2003 – II International Materials Symposium, April 14*–*16, Campus da Caparica, Portugal. Some other results gathered in the frame of running collaborative project (*Project FCT SAPIENS, Ref.: POCTI/1999/CTM/35470*, *“Processing and High Temperature Mechanical Characterisation of Cordierite-Based Ceramic Foams (CORDFOAM)”*, 2001−2003), aiming at the fabrication of cordierite foams by the polymeric sponge method, intended for gas burner applications, were also presented in the same conference [F.A.C. Oliveira, D.M. Dias, J.M.F. Ferreira, S. Olhero, D. Dias, Fabrication of cellular cordierite foams, Mater. Sci. Forum 455–456 (2004) 177–181. doi: 10.4028/www.scientific.net/MSF.455-456.177.]. In both cases, the CICECO team was the responsible for the processing tasks. The institution of the first author was the partner responsible for developing a prototype machine to squeeze the excess slurry from impregnated sponges, stared wide-eyed looking at the HA Poster, congratulating and praising the PhD student of Prof. Ferreira for the important achievement. He didn't move away without first quizzing from her all the relevant information. He could then easily realise that the same procedural approach (including processing additives) was followed for both materials, HA and cordierite, the essential processing details for the last one had been clearly reported in the deliverables from CICECO. Armed with this information, he quickly and recruited a Master student and offer her the secure topic of fabricating porous CaP scaffolds by the polymeric sponge method, using the knowledge and recipe from CICECO for HA, and the prototype machine for squeezing the excess slurry from impregnated sponges. This allowed them successfully achieving the target within a few months. In November 5, 2003 (World Materials Day), the author of the Master thesis was awarded the prize from the European Federation of Materials Societies for her Master Thesis “*Manufacture of Hydroxyapatite Foam for Medical Applications*”. The prize was publicly delivered to the recipient in December 2004, during the Bioceramics conference in Porto, Portugal. Prof. Ferreira felt deeply shocked by the unethical omission of the due credits to the source of knowledge, but he restrained himself so as not to ruin the party, conveying privately his feelings to the supervisor.

Initially, they attempted hand over and disclose the know-how to different potential partners doing research in biomaterials. Some of them later shameless appeared making presentation in conferences as being the owners of the results. The know-how was then informally transferred to CERAMED, including the author of the Master thesis and the prototype machine for squeezing the excess slurry from impregnated sponges. Soon, the author of the Master thesis realised that CERAMED would not give anything in return to them and secretly decided to patent the know-how, thus hindering CERAMED to unfairly exploit it commercially. Later, in 2008, she founded her own company Medbone® – Biomaterials. According to the information in its site (<https://www.medbone.eu/en/>), the manufactured synthetic bone grafts are being commercialized and used worldwide in more than 90 countries, in orthopaedic, dental and veterinary surgeries.

***Creation of a spin-off from Prof. Ferreira group***

Challenged by the promising results presented at the first annual meeting (Jornadas do CICECO – 2003) by Prof. Ferreira, the Dean seem truly committed to make something happen in Chemistry department to put it at the protagonist cutting-edge in accomplishing the CICECO mission. He focused all his energies in warming there the innovative environment, including coaching entrepreneurship courses ministered by national and international specialists for some selected candidates, who were given the opportunity to test the solidity and potential business viability of their technological ideas. The winning idea would receive a 25,000 € prize and an approved business plan. Team members of Prof. Ferreira had no room there. Therefore, 3 of his young researchers working with CaP-based powders and scaffolds created, in September 2004, a pioneering spin-off in this area in Portugal, “*Agoramat – Produção de Materiais Cerâmicos, Lda*”. The aim was to ally research, development and production of powders and finished products for biomedical applications. Soon after, the shareholder structure was enlarged to include Prof. Ferreira, and two medicine doctors, a Haematologist from “Biosckin – Molecular and Cell Therapies, S.A.” and an Orthopaedist from “Artur Salgado, S.A.”. This last company used to commercialize a variety of CaP-based bone grafts and metallic implants, used in the orthopaedic surgical operations made by its orthopaedists, and was willing to join the production team to replace the imports, and help defining some tailor-made features.

In 2005, *Agoramat* was short listed among 307 proposals, by a partnership led by IAPMEI with the strategic objective of boosting innovative and technology-based entrepreneurship in Portugal, and was recipient of the Empreenda 2005 prize. In the same year, *Agoramat* also received the 1^st^ prize (6 months-free hosting) in the 2^nd^ Competition for the Creation of Innovative Companies with a Technological Base - “Associação da Incubadora do Beira Atlântico Parque (AIBAP)”, Mira, Portugal. Unfortunately, this last one revealed to be a poisoned gift: the main building of AIBAP was still under construction, and *Agoramat* was hosted in a pre-existing cowshed, clumsily and inadequately adapted to the purposes. As a result, after one year, the electrical power was not yet enough to put the production equipment working. Thus, *Agoramat* decided to abandon AIBAP, rent a new space and create suitable conditions there for the production of biomaterials. A bank loan had to be taken to face the added incurred costs.

Being the first and single hosted company then far, and the incubator did not agree with the move away of *Agoramat* and closed the doors to prevented the removal of equipment. They could be released only several months later, enforced by a court decision. All these setbacks delayed the achievement of the objectives, and consumed an excessive portion of the scarce available financial resources. This reduced the *Agoramat*’s breath to reach the most advanced testing and validation, feasibility studies, and regulatory clearances for Class III Medical Devices.

Even though, *Agoramat* could be viable just by selling its high-quality FORCOAT HA powder for plasma spraying to a partner company, *CERAMED – Cerâmicos para aplicações médicas* (<https://pitchbook.com/profiles/company/61172-47#overview>). But *CERAMED*, was more interested in absorbing *Agoramat*'s knowledge and technology than in ensuring its viability, and intentionally, only purchase small and insufficient amounts. Several years later, *ALTAKITIN* ([http://www.pofc.qren.pt/media/noticias/entity/altakitin](http://www.pofc.qren.pt/media/noticias/entity/altakitin~)) a branch company of *CERAMED – Cerâmicos para aplicações médicas*, acquired the actives of *Agoramat* and kept two of its staff members to endogenize the know-how, while *CERAMED – Cerâmicos para aplicações* changed its name to *CERAMED Revestimentos*. Recently, *Bioceramed* upsurge, as a spinoff of *CERAMED Revestimentos*, being allegedly a partner of *Hydrumedical* (*[Hydrumedical - Developing the Next Generation of Medical Technologies](https://hydrumedical.pt/news/)*), Avepark - Parque de Ciência e Tecnologia, Guimarães , Portugal, for CaP-based bone grafts and technologies heritage from *Agoramat*.

***Other entrepreneurship initiatives by the Dean***

The Dean was very eager to make something to happen in the department to which he belonged. Unsurprisingly, the winning business idea in the frame of the first entrepreneurship course coached by national and international experts was from Chemistry Department. Strangely, from Food Chemistry, not belonging to the CICECO research lines. But this was not an impediment to receive all the CICECO and institutional enthusiastic support. Besides the 25,000 € prize and an approved business plan, the institutional support also dragged at least other three equal 25,000 € prizes from different banks willing to be inside the party.

In 2005, the Director of CICECO proudly announced through an e-mail message to all members, the formation of the first CICECO spinoff “*FoodMatrix*” that he was also a part of. Soon after, the joy and pride turn disappointment upon being informed that the first CICECO spin-off was *Agoramat*. The *FoodMatrix* activities never started and, at the end, they had nothing to show to the Portuguese Foundation for Science and Technology (FCT). Later, *Agoramat* was waived to the funding institution, as an important CICECO achievement, while its promoters were honoured with internal bullying.

***Animal Farm like distribution of resources***

FCT provided founds to contract about 36 new post-doc investigators to reinforce the CICECO research lines. Twelve (12) of them were assigned to the Directors’ group; while others directly with members of the extended steering direction (*i.e.*, coordinators of research lines or research groups), without following any rational criteria. Moreover, following an internal rule, two thirds of the total pocket money were distributed among the CICECO members according to the scientific productivity, and the remaining third was distributed equally. In accordance with those rules, and since the beginning, Prof. Ferreira used to be systematically ranked in the first position, with gradual increasing differences to the second one, the Director, as years passed. This output was somewhat misleading, as the parcels sum-up from the numerous Director’s group members was significantly higher. Even though, the apparent output of annual evaluation was regarded with fat eyes. Therefore, after seven years, CICECO amended the main commandment of the internal rule to: “*Everything remains the same, but no member will be allowed to receive more than 3-times de average*”. By the way, along all the years preceding this change, only Prof. Ferreira had received, in the most recent ones, more than triple the average, a topic has been brilliantly versed by George Orwell in the anti-utopian satire - Animal Farm – published in 1945 ...

***The man at the helm without a compass***

Aiming to fill the shortage of strategic ideas, the Rector elected in 2010, and his closer staff, visited the departments asking for contributions. When they visited DECV, about 3-months after of being empowered in the function, the starting of the conversation was boring and aimless. Then, Prof. Ferreira proposed to think at a better integration of the multidisciplinary research areas dealing with biomaterials, to prove their biological performance, while considering also competences from business and management. He also suggested to explore and enlarge collaborations with veterinary clinics for *in vivo* tests, and with hospitals for clinical tests, as there was perceived willingness from potential partners, but waiting for formal institutional understandings.

The example of Apatech (an English start-up that followed all the strategic roadmap steps, in terms of research and the translation of the results from the bench towards the market), was pointed out as an interesting Case Study. After about 10 years of its existence, Apatech was acquired by a USA multinational by more than 330 million USD. The overall idea, was considered exciting and very interesting! Based on it, the Rector gathered people from different departments and challenged them to prepare a QREN project proposal in the area of regenerative medicine, under the coordination of the CICECO Director. The project ended up being financed with more than 1 million €. Unfortunately, the mentor of the idea was just ignored in all this process. He only became aware about it in 2012, when the PI announced a few post-doc grants and he went there to talk with him about a potential candidate to one of the post-doc grants. This opportunity of doing something worthy was essentially lost: a significant expenditure of resources for very little.

***The punishment of the hardest workers***

Along several years (2009–2012), Prof. Ferreira was accumulating part of his pocket money to purchase a Robocasting machine for additive manufacturing. In the middle of 2012, he requested the Director of CICECO and the Rector, to allow purchasing the equipment. Unfortunately, no action was taken towards this. Instead, by the end of 2012, the University of Aveiro used his accumulated pocket money for something else. When questioned about the reasoning behind, they gave no explanation but shameful lies and threats. The money was never returned, and the Robocasting machine was only purchased 3 years later, delaying the accomplishment of several MSc and PhD theses that included additive manufacturing in their work programs. Because of this misappropriation, in the first half of 2013, after paying the costs of the *in vivo* experiments carried out for Alkali-Free Bioactive Glass in a sheep animal model [256], the team’s budget became exhausted, with considerable damages and delays for the running research activities. Very disappointing and discouraging being Boxer, the workhorse, in such a vivid Animal Farm...

**Figure captions**

***Fig. SI–1.*** *Hydroxyapatite-based ceramics mimicking the porous trabecular and dense cortical bone structures (a) fabricated by: (1 – slip casting + polymeric sponge method; 2,4 – polymeric sponge method; 3 – slip casting + foaming); (b) dense maxillofacial prostheses for facial reconstruction fabricated by the starch consolidation, dried and sintered at 1300 °C).*

***Fig. SI–2****. Starch consolidation – suitable to produce from almost full dense to very porous materials with complex shapes: (a) Porous scaffold; (b) Examples of complex shaped alumina ceramics obtained by SC; (c) A green massive alumina cylinder after being further lathe shaped in a machine shop, as a demonstration of the high green strength; (d) Disk shaped samples with machined grooves and holes in the green state. The larger sintered disk was intended as support for heat treating glass lamella coated by sol-gel. Adapted with permission from [350].*

**Figure SI-1**

**Figure SI-2**


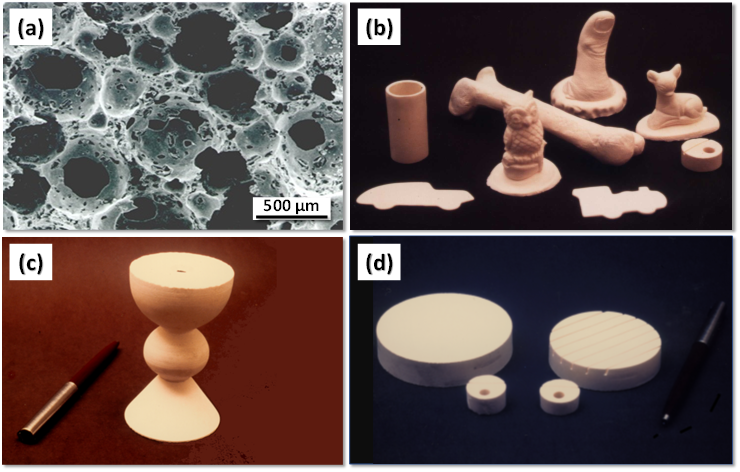

Supplement: Multimedia component 1 [file mmc1.docx]
